# Automating structure–activity analysis for electrochemical nitrogen reduction catalyst design through multi-agent collaborations

**DOI:** 10.1093/nsr/nwaf372

**Published:** 2025-09-04

**Authors:** Xu Hu, Suya Chen, Letian Chen, Huijuan Wang, Xu Zhang, Zhen Zhou

**Affiliations:** School of Materials Science and Engineering, Institute of New Energy Material Chemistry, Renewable Energy Conversion and Storage Center (RECAST), Key Laboratory of Advanced Energy Materials Chemistry (Ministry of Education), Nankai University, Tianjin 300350, China; School of Materials Science and Engineering, Institute of New Energy Material Chemistry, Renewable Energy Conversion and Storage Center (RECAST), Key Laboratory of Advanced Energy Materials Chemistry (Ministry of Education), Nankai University, Tianjin 300350, China; School of Materials Science and Engineering, Institute of New Energy Material Chemistry, Renewable Energy Conversion and Storage Center (RECAST), Key Laboratory of Advanced Energy Materials Chemistry (Ministry of Education), Nankai University, Tianjin 300350, China; School of Materials Science and Engineering, Institute of New Energy Material Chemistry, Renewable Energy Conversion and Storage Center (RECAST), Key Laboratory of Advanced Energy Materials Chemistry (Ministry of Education), Nankai University, Tianjin 300350, China; Interdisciplinary Research Center for Sustainable Energy Science and Engineering (IRC4SE2), School of Chemical Engineering, Zhengzhou University, Zhengzhou 450001, China; Longmen Laboratory, Luoyang 471023, China; School of Materials Science and Engineering, Institute of New Energy Material Chemistry, Renewable Energy Conversion and Storage Center (RECAST), Key Laboratory of Advanced Energy Materials Chemistry (Ministry of Education), Nankai University, Tianjin 300350, China; Interdisciplinary Research Center for Sustainable Energy Science and Engineering (IRC4SE2), School of Chemical Engineering, Zhengzhou University, Zhengzhou 450001, China

**Keywords:** large language models, multi-agent collaboration, nitrogen fixation, rational catalyst design, structure–activity relationships

## Abstract

The electrochemical nitrogen reduction reaction (eNRR) offers sustainable ammonia production, yet elucidating structure–activity relationships (SARs) is challenging. We introduce eNRRCrew, a novel multi-agent framework integrating large language models (LLMs), machine learning and automated tools to advance eNRR research. By analyzing 2321 papers, eNRRCrew constructed a comprehensive database of electrocatalyst properties, conditions and performance. The framework employs a random forest classifier for eNRR yield prediction, with model interpretability analysis revealing key factors like space group number and elemental electronegativity difference. Additionally, clustering analysis identifies distinct Faradaic efficiency patterns. eNRRCrew's five LLM agents enable natural language interaction for novel catalyst recommendation, performance prediction, data analysis and literature insights. This approach surpasses traditional methods in extracting SARs and guiding rational catalyst design, offering a scalable platform for various electrocatalysis domains and a new paradigm for LLM-driven scientific discovery.

## Teaser

eNRRCrew, a novel multi-agent large language model framework, automates the complex design of catalysts for sustainable ammonia production, thereby accelerating AI-driven scientific discovery.

## INTRODUCTION

The electrochemical nitrogen reduction reaction (eNRR) offers a promising sustainable alternative to the energy-intensive Haber–Bosch process for ammonia (NH_3_) production, which currently consumes about 1%–2% of global energy and generates substantial CO_2_ emissions [1,2]. Despite significant research efforts to develop efficient electrocatalysts for eNRR, the field faces several critical challenges [3,4], including low Faradaic efficiency (FE), limited NH_3_ yield, and an incomplete understanding of the structure–activity relationships [5]. Specifically, the effects of the electrocatalyst, reaction conditions such as pH, applied electrode potential and electrolyte on eNRR performance are not fully understood. These challenges are further complicated by the rapidly expanding literature and the complex interplay of multiple factors affecting catalyst performance [6].

Traditional approaches to analyzing structure–activity relationships in eNRR have relied heavily on experimental trial-and-error methods and isolated case studies, which are both time-consuming and limited in their ability to identify broader patterns across diverse electrocatalytic systems [7]. Though computational methods such as density functional theory (DFT) have provided valuable insights into reaction mechanisms [8–11], they often struggle to capture the intricate complexities of the realistic electrocatalytic environment and experimental conditions. Furthermore, the vast amount of published research in eNRR contains valuable information that remains largely untapped due to the challenges in systematically analyzing and correlating diverse data sources. Natural language processing (NLP) techniques have emerged as powerful tools for scientific literature mining and knowledge extraction. These approaches have demonstrated success in various scientific domains, enabling automated extraction of chemical information, materials properties and synthesis procedures from publications [12]. However, traditional NLP techniques, which rely on rule-based approaches or require extensive pre-training on large sets of high-quality specialized texts, often encounter limitations when dealing with specialized scientific content. These challenges include difficulty in accurately representing complex chemical structures, experimental parameters and performance metrics, leading to poor scalability, low accuracy and high labor cost.

Recent advances in artificial intelligence (AI), particularly in large language models (LLMs), such as ChatGPT, Llama and DeepSeek, offer new opportunities to address these challenges [13,14]. These tools have demonstrated remarkable capability in natural language understanding and pattern recognition across various scientific domains [15–17]. However, their application to complex electrocatalytic systems like eNRR has been limited by challenges in handling specialized scientific knowledge and the need for robust data extraction and analysis frameworks. In addition, an inherent limitation of LLMs is their restricted capability to reason and understand complex scenarios. These models often struggle with intricate reasoning tasks due to their reliance on patterns in the training data rather than genuine comprehension, known as hallucination [18]. This limitation becomes particularly evident in specialized scientific research where a deeper understanding of nuanced and context-specific information is necessary, like the eNRR field. LLM agents [19] serve as intelligent systems that bridge the gap between LLMs and practical applications, making significant impacts in various fields like human behavior simulation [20], chemistry [21–24], bioinformatics [25] and paper searching [26]. By leveraging techniques such as function calling to interact with surrounding environments, these agents can execute complex tasks, providing researchers with tools to navigate and manipulate vast datasets efficiently and automatically.

Based on the context outlined above, we present eNRRCrew, a multi-agent collaboration framework that integrates LLMs, knowledge graphs, machine-learning algorithms and automated data analysis tools to advance eNRR research. This system combines ChemPrompt-enhanced LLMs for structured data extraction [15], sophisticated machine-learning models for performance metrics prediction, and specialized LLM agents for interactive analysis. By analyzing 2321 paper abstracts, we have developed a comprehensive database of electrocatalyst properties, reaction conditions and performance metrics, enabling systematic investigation of structure–activity relationships in eNRR. A core capability of this framework is its power to not only analyze existing catalyst data but also to actively recommend novel electrocatalyst candidates and forecast their potential performance. The aim of this integrated approach is to provide researchers with powerful tools for rational catalyst design and optimization, potentially accelerating the development of practical eNRR systems for sustainable NH_3_ production.

## RESULTS AND DISCUSSION

**Figure 1.**
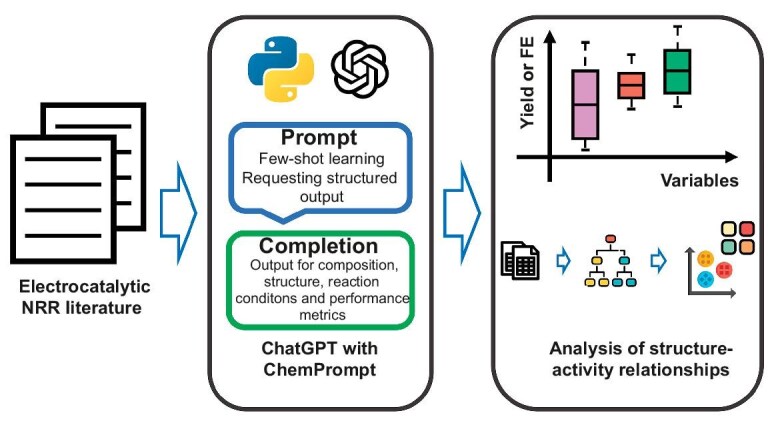
Schematic workflow for predicting and analyzing structure–activity relationships in eNRR literature using LLMs with ChemPrompt. The process involves few-shot learning and requesting structured output for generating outputs related to composition, structure, reaction conditions and performance metrics, followed by statistical analysis and machine learning. This figure has been designed using resources from Flaticon.com.

High-quality data extraction through text mining is the first crucial step in analyzing structure–activity relationships. However, traditional approaches rely heavily on rule-based methods like regular expressions, which suffer from poor generalizability and low accuracy. Here, we introduce LLMs to the text-mining process to achieve efficient and accurate data extraction. The workflow depicted in Fig. 1 outlines a systematic approach for predicting and analyzing structure–activity relationships in eNRR literature using LLMs with ChemPrompt. The process begins with extracting relevant data from the eNRR abstract corpus. The data are then input into a prompting framework that utilizes both few-shot learning [27] and requesting structured output techniques. These prompting methods enable LLMs, specifically GPT-4o integrated with ChemPrompt, to generate outputs related to electrocatalyst composition, structural features, reaction conditions and performance metrics, such as yield and FE. The generated outputs are then analyzed to establish relationships between electrocatalyst characteristics, reaction conditions and performance metrics using statistical analysis and machine-learning techniques. This approach allows researchers to identify optimal electrocatalysts and reaction conditions. The overarching goal is to enhance the understanding of structure–activity relationships in eNRR, facilitating more informed and effective catalyst design in future research.

### Text mining with LLMs

#### Description of the literature corpus

**Figure 2.**
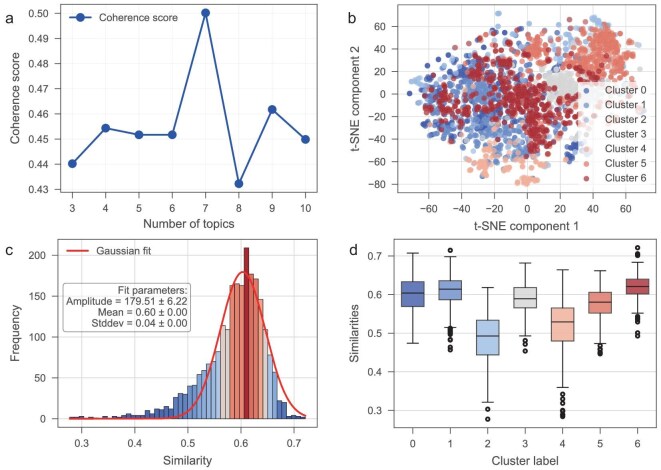
Analysis of the literature corpus on eNRR. (a) Coherence score as a function of the number of topics. (b) t-SNE plot visualizes paper clustering based on topic distribution, with different clusters (0 to 6) representing various research focuses in eNRR. (c) Histogram of similarity scores between the typical eNRR abstract and the abstracts in the corpus. (d) Box plot categorizing similarity scores by cluster labels.

In this study, we systematically analyzed a literature corpus comprising 2321 paper abstracts retrieved from the Web of Science database (see METHODS for details). These papers were carefully selected with a targeted search query designed to capture a wide array of research within the field of eNRR. To further analyze the corpus, we applied topic modeling techniques with Latent Dirichlet Allocation (LDA) to identify underlying themes within the literature, as shown in Fig. 2a. The results reveal an optimal number of seven topics, achieving a peak coherence score of 0.50. The top 25 representative words for each topic, which provide detailed insights into the thematic composition, are provided in Table S1. In addition to topic modeling, we employed t-distributed stochastic neighbor embedding (t-SNE) to visualize the clustering of papers based on topic distribution, illustrated in Fig. 2b. The resulting t-SNE plot revealed different clusters; each cluster highlights a specific focus, ranging from efficiency optimization, synthesis processes, materials properties, molybdenum-based catalysts, experimental methods and theoretical studies to general performance enhancements. For a comprehensive analysis, please refer to the detailed explanation of Table S1. Understanding these clusters can help guide future research directions and identify key areas for further investigation and development in eNRR technology.

Cluster analyses reveal that the corpus includes papers involving DFT calculations. However, since our primary objective is to analyze the factors influencing eNRR yield—a metric that cannot be directly obtained through DFT calculations, these papers are not central to our study. Therefore, we manually constructed a typical experimental eNRR abstract template. This template included essential details such as electrocatalyst composition, structure, reaction conditions and performance metrics like FE and yield. A subsequent similarity analysis, as shown in Fig. 2c (see METHODS for details), reveals the distribution of similarity scores ranging from approximately 0.3 to 0.7, with a prominent peak centered at a mean value of 0.60. This indicates that a substantial portion of the literature exhibits moderate to high alignment with our template, providing a solid foundation for extracting meaningful structure–activity relationships in eNRR research. The box plot presented in Fig. 2d categorizes the similarity scores by cluster labels derived from previous topic modeling and clustering analyses. Clusters 0, 1 and 6 demonstrate higher median values compared with the others, indicating a concentration of research focused on catalyst performance and optimization strategies. These results suggest that the collected abstracts are comprehensive and diverse, serving as an excellent corpus base for LLMs to extract structure–activity relationships in eNRR.

#### Automated structure–activity relationship extraction workflow

Following the three ChemPrompt engineering principles [15], which involve minimizing hallucination by grounding responses in the source text, providing detailed instructions and requesting a structured output format, we utilize carefully crafted prompts to guide ChatGPT in generating structured summaries about eNRR by requiring the information to be organized in a table. While formats like JSON or YAML are excellent for hierarchical data [28], we selected a tabular output for its direct compatibility with machine-learning workflows. This approach allows the extracted information to be seamlessly mapped into a feature matrix, eliminating the need for additional parsing and flattening steps that are often required for nested formats. The high accuracy achieved with this method (average F1 score of 0.96, as discussed below) validates the effectiveness of combining few-shot prompting with a structured tabular output for reliable data extraction. The prompts are designed to elicit comprehensive responses from the corpus that include essential parameters such as the name of the electrocatalyst with corresponding elemental and morphology information, FE, NH_3_ yield, applied electrode potential, electrolyte information, synthesis methods, stability tests and N-15 labeling. To ensure that the information collected is exclusively pertinent to eNRR, we also guide ChatGPT to gather information about the type of reactions, reactants and products, which is conducive to subsequent filtering and analysis of the collected data. Moreover, the prompt integrates a series of judgment criteria that assess various morphology features of the catalysts based on the literature review [12,29], including dimensionality and specific material characteristics, such as two-dimensional (2D) catalysts, nanoparticles, single-atom catalysts and heterostructures, which are highly used in the eNRR experiments to optimize the electrocatalytic performance by structure engineering. A few-shot prompt strategy was employed to assist ChatGPT in accurately identifying and summarizing relevant terms within the corpus by incorporating contextual information and examples. Specifically, abstracts that include information about various electrocatalysts, reaction conditions or photocatalytic NRR, as well as those lacking details on the structure–activity relationship in eNRR, were incorporated into the prompt. This approach helps mitigate hallucinations within ChatGPT and enhances the accuracy and reliability of the responses. Once the summarized tables are generated, a dedicated function then converts the summarized text into a structured table format, enhancing clarity and supporting deeper analysis. The text mining workflow is more powerful, effective and concise compared with traditional NLP methods, providing an automated and efficient solution for processing large volumes of electrocatalysis literature data, making it more versatile and universally applicable. The detailed prompt and script are shown in Sections 9 and 10 in the Supplementary data.

To rigorously validate the accuracy of information extraction using LLMs, we conducted comprehensive performance evaluations and error analyses. Specifically, we performed a comparative study between our current GPT-4o model and the recently released DeepSeek-v3 and DeepSeek-R1 models. A random sample of 50 abstracts was selected from our corpus for manual parameter extraction, which served as our ground truth dataset for validation against the LLM-extracted results. The performance assessment encompassed 14 key NH_3_ synthesis parameters, with evaluation metrics including precision, recall and F1 scores, as shown in Figs S1 and S2. Our framework, based on the GPT-4o model, demonstrated exceptional performance with average precision and recall, and F1 scores of 0.97, 0.95 and 0.96, respectively (Fig. S3). These robust metrics substantiate the reliability of our methodology and establish a solid foundation for subsequent machine-learning model development. While the DeepSeek-v3 model exhibited strong instruction-following capabilities, it occasionally encountered ‘lost in the middle’ phenomena [30], resulting in parameter extraction failures. Consequently, its recall and F1 scores were marginally lower than those achieved by GPT-4o. For the DeepSeek-R1 model, its tendency to generate extraneous conversational text disrupted the automated pipeline, and its complex reasoning process occasionally led to the mismatching of performance parameters from the same abstract, ultimately resulting in lower accuracy and significantly slower processing times for this structured extraction task.

### Machine-learning model development

#### Data preprocessing and feature engineering

A total of 1837 eNRR-related data entries were identified where the reaction type is electrochemical, the reactant is N_2_ and the product is NH_3_. Data entries with missing values for FE and yield are excluded. A detailed characterization of the elemental distribution within the dataset is provided in the Supplementary data (Fig. S4). Using the extracted information about the electrolytes and applying chemical knowledge, we constructed pH-related descriptors, and a detailed script is shown in Section 11 in the Supplementary data. We employed Magpie [31] to develop descriptors associated with the elemental composition of electrocatalysts. Structural information is collected based on predefined judgment criteria. However, some criteria, such as Judge_MOF and Judge_COF have insufficient data with fewer than five entries. To address this issue, we consolidated all judgment criteria and employed ChatGPT to classify and automatically construct catalyst structure descriptors with natural language. The relevant prompt and code are included in Section 12 in the Supplementary data. After data preprocessing and feature engineering, the dataset comprises 159 descriptor variables, as listed in Table S2, and 1117 entries, which we utilized for the subsequent statistical analysis and development of machine-learning models (see METHODS for details).

#### Yield prediction

Using the curated dataset, we observed that the extracted yield primarily consists of two unit types: μg h^−1^ mg_cat_^−1^ for the unit mass of the electrocatalyst and μg h^−1^ cm^−2^ for the unit area of the electrocatalyst. We developed a machine-learning classification model to predict the yield, categorizing yields equal to or above the median as high yield. The distribution of eNRR yield in the dataset is shown in Fig. S5. Contrary to an ideal normal distribution, it reveals that both yield metrics exhibit a non-normal, right-skewed character. The yield in unit mass (median 26.15 μg h^−1^ mg_cat_^−1^) is heavily concentrated at lower values with a long tail extending towards higher yield, while the yield in unit area (median: 12.26 μg h^−1^ cm^−2^) displays an even more pronounced skew. Both values are significantly below the minimum yield threshold for practical eNRR, set at 100 nmol s^−1^ cm^−2^, assuming an electrocatalyst loading of about 1 mg cm^−2^ [32,33]. This indicates that there is still a vast space for exploration in the design of electrocatalysts and the optimization of reaction conditions, and underscores the importance of data-driven approaches in the pursuit of high eNRR yield. Based on the obtained dataset, we conducted a statistical analysis of descriptors related to eNRR yield. For descriptors related to electrolyte, pH type and electrocatalyst morphology, different categories do not show significant differences in yield, as demonstrated in Figs S6–S8 and S10–S12. Additionally, it is evident that there is no significant correlation between yield and various elemental property descriptors, as the absolute values of the correlation coefficients for unit in area and unit in mass are both below 0.26, as shown in Figs S9 and S13. This indicates that the yield is influenced by a complex interplay of multiple factors, and no single descriptor can solely determine yield levels. Given this complexity, the use of machine learning becomes essential, which can handle multifaceted data and uncover intricate patterns that statistical methods might miss. Furthermore, we elaborate in Fig. S14 on the concept of ‘reactivity cliff’ [34,35] as a key theoretical basis for our choice of a classification model over regression. To empirically substantiate this decision, we also provide a detailed statistical analysis in Figs S16 and S17 of our unsuccessful attempts to build a predictive regression model using ROBERT [36]. We considered several machine-learning models, including random forest classifier, support vector classifier, eXtreme Gradient Boosting (XGBoost) classifier and artificial neural networks. After performing recursive feature elimination, a total of 152 features were retained. The corresponding correlation coefficient matrix can be found in Fig. S15. Eighty percent of the dataset was allocated for training, while the remaining 20% was set aside for tests, and the performance of the classification model was then assessed with various metrics, including the confusion matrix, the receiver operating characteristic (ROC) curve, and the area under curve (AUC). Using Optuna [37] to find the optimal hyperparameters, we discovered that the random forest classifier performed the best, as demonstrated in Table S3.

**Figure 3.**
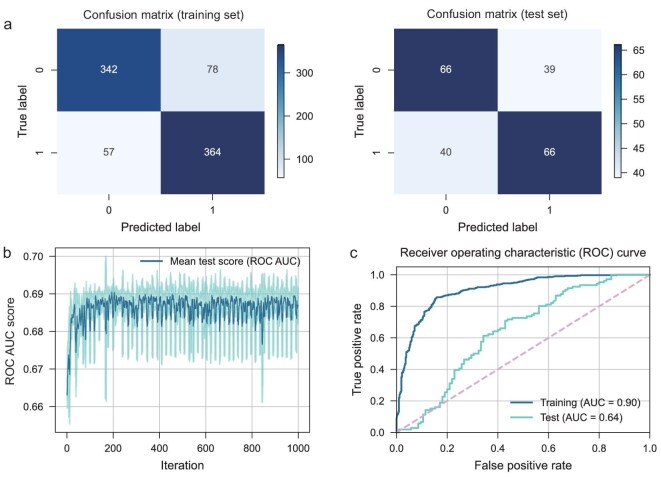
Performance evaluation and feature importance analysis of the random forest classifier. (a) Confusion matrix for the random forest classifier on the training set (left) and test set (right). (b) Mean test scores (ROC AUC) over iterations during hyperparameter tuning with Optuna. (c) ROC curve, showing the true-positive rate against the false-positive rate, with AUC values indicating model effectiveness.

The confusion matrices in Fig. 3a reveal that the classifier achieved a prediction F1 score of 0.84 for the training set and 0.63 for the test set, demonstrating its effectiveness in distinguishing between high and low yields. As shown in Fig. 3b, the mean test scores (ROC AUC) over iterations during hyperparameter tuning with Optuna show that after 340 iterations, the scores stabilize without further improvement, indicating that Optuna has successfully identified the optimal hyperparameters. The optimal hyperparameters can be found in Table S4. Additionally, the AUC values of 0.90 for the training set and 0.64 for the test set in Fig. 3c further suggest its predictive performance. This level of performance is comparable to values reported in structure–activity prediction studies based on experimental data in the electrocatalytic reactions [11,38], for example, a classification model predicting CO_2_ reduction reaction (CO_2_RR) indicators achieved similar results on over 20 test samples [38]. Considering that the yields of eNRR are generally low and far from meeting practical application standards, and that there is a lack of data with truly ‘high’ yields, we believe that the model's performance will significantly improve as the data quality is enhanced. To better understand the error origin, we conducted an error analysis on the misclassifications made by our classification model, as shown in Figs S18 and S19. Notably, the current accuracy provides valuable insights into the structure–activity relationship as discussed below. To further assess the predictive capability of the random forest model, we also implemented the k-nearest neighbors (KNN) algorithm for eNRR yield prediction. As shown in Fig. S20, the results indicate that when k = 1 (which is particularly relevant as it aligns with chemical intuition) [39], the random forest model significantly outperforms the KNN baseline model across several metrics, including macro recall, accuracy, precision, F1 score and ROC AUC. This observed superiority suggests that the random forest model exhibits stronger generalization ability, underlining the importance of utilizing advanced machine-learning techniques in this context. Also, the localized comparison is crucial for understanding catalyst performance and reinforces the necessity of using models like random forest models that can capture complex relationships more effectively.

To get the implication of structure–activity relationships, we used Shapley additive explanations (SHAP) analysis to quantify the contribution of each feature to the model's predictions, as shown in Fig. S21. A small ‘MagpieData mean SpaceGroupNumber’ value shows a significant contribution to increasing the predicted eNRR yield. Elements with small space group numbers typically exhibit simpler symmetry and fewer symmetry operations, contributing to more isotropic physical properties and easier combinations with other components, leading to enhanced stability and performance [40]. ‘Electrolyte without concentration (Na_2_SO_4_)’ and ‘pH (weak acid)’ also show high SHAP feature importance, which may be associated with the suppression of the hydrogen reduction reaction [41]. Also, a greater difference in electronegativity among various elements in the electrocatalyst is associated with a higher eNRR yield. This correlation may be due to the enhanced charge transfer [42], the modulation of the d-band center of the central metal [43], or the generation of Lewis acid sites that promote N_2_ activation and inhibit H adsorption [44]. The statistical robustness of these feature importances was validated through a bootstrap analysis, and a detailed discussion is provided in Fig. S22.

#### FE clustering

**Figure 4.**
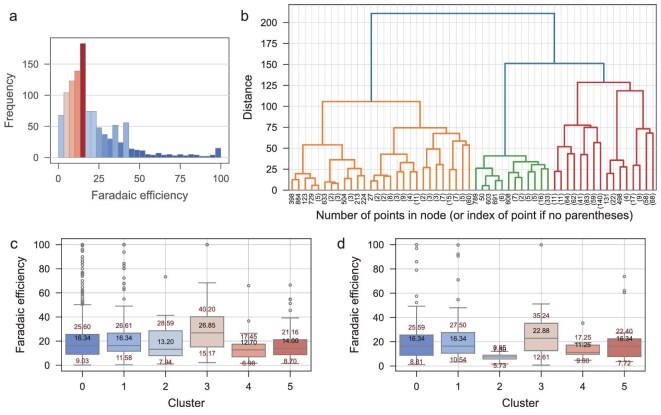
FE prediction and cluster analysis with machine-learning models. (a) Histograms illustrating the distribution of FE data. (b) The dendrogram was generated from the hierarchical clustering of descriptors for FE using linkage. (c) Cluster results from agglomerative clustering on the training set, with (d) showing the results of a Nearest Centroid classifier trained on the training set to assign the test set to the nearest cluster.

FE is another essential eNRR performance metric relevant to energy consumption, sustainability and the environmental impact of NH_3_ production [45]. The distribution of FE in the dataset is illustrated in Fig. 4a, and the values exhibit a notable concentration of around 20%, which is far below the practical performance target of 90% FE [46]. It underscores the necessity for data-driven strategies to enhance the FE in future research efforts, the same as for the eNRR yield. The results presented in Fig. S23 indicate that the performance of the random forest regression model in predicting FE is poor, as evidenced by the low R^2^ values of 0.24 for the training set and 0.12 for the test set, which is comparable to that of the recently published machine-learning models for predicting CO_2_RR production rates [11]. Mean test scores [negative mean squared error (MSE)] over iterations during hyperparameter tuning using Optuna are shown in Fig. S24. This illustrates that the regression model struggles to accurately capture the underlying relationships between the descriptors and the FE, which demonstrates that the factors influencing FE are more complex, involving side reactions such as the hydrogen evolution reaction.

The regression model's poor predictive performance suggests that the dataset contains underlying heterogeneity or complex substructures that a single model cannot adequately capture. Given the limitations of the regression approach, we explored alternative analytical methods to gain deeper insights into the factors affecting FE. Clustering approaches can reveal patterns and groupings within the data that may not be immediately apparent [47], allowing for a more nuanced understanding of how different factors contribute to FE. By identifying clusters with distinct characteristics, researchers can pinpoint specific conditions or features that lead to improved performance, thereby informing future experimental designs and catalyst development. In light of these findings, we proceeded to train a clustering model using agglomerative clustering techniques, as shown in Fig. 4b. Based on the dendrogram, we selected six categories, and the clustering results shown in Fig. 4c and d demonstrate improved performance, particularly in distinguishing between different clusters within the dataset. Notably, Cluster 3 exhibits significantly higher median values for FE compared with other clusters, with its 25th and 75th percentiles also surpassing those of the remaining clusters. This indicates that samples within Cluster 3 are associated with more favorable outcomes, suggesting that the features characterizing this cluster may be particularly advantageous for higher FE. As shown in Fig. S26, properties related to the elemental composition of the catalysts, such as the number of valence electrons in different orbitals and atomic mass, play a significant role in determining FE. This finding is consistent with previous studies that identified the electron number of constituent elements as an integral component of the universal descriptor for eNRR [48]. Detailed mechanistic discussion connecting these elemental descriptors to the underlying electrocatalytic principles that govern eNRR selectivity is provided in the note for Fig. S26.

### eNRRCrew, a multi-agent collaboration framework

**Figure 5.**
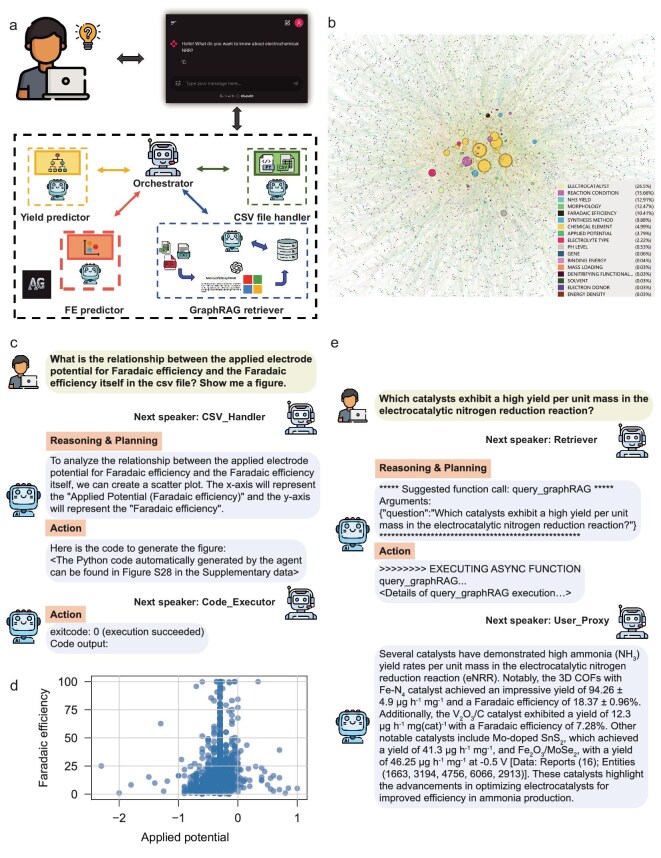
A comprehensive illustration of eNRRCrew. (a) Schematic image of eNRRCrew. This figure has been designed using resources from Flaticon.com. (b) The LLM-generated knowledge graph of the eNRR corpus was built using GPT-4o-mini. The size of each node represents the number of edges associated with it, while the color indicates the category to which the node belongs. The visualization was conducted by Gephi. (c) Dialogue showing the eNRRCrew providing Python code to analyze the relationship between applied electrode potential and FE. (d) The scatter plot generated from the eNRRCrew automatically illustrates the relationship between applied potential and FE. (e) Dialogue showing the eNRRCrew providing the information about which catalysts exhibit a high eNRR yield per unit mass.

Based on the corpus that we obtained from the text-mining workflow, the curated database and the machine-learning models for predicting eNRR yield and FE, we have developed a multi-agent collaboration framework called eNRRCrew, as depicted in Fig. 5a (see METHODS for details of eNRRCrew). This crew consists of five agents, each based on LLMs, specifically using GPT-4o and GPT-4o-mini in this work. The five agents are: an orchestrator, which allocates tasks to different agents based on user input; a yield predictor and an FE predictor, which uses pre-trained machine-learning models in the former section to predict eNRR yield and FE; a GraphRAG [49] retriever, which enhances responses based on a knowledge graph (Fig. 5b) constructed from eNRR abstracts and recommends new electrocatalytic systems not included in existing databases; and a CSV file handler, which writes and executes code to interact with CSV files obtained from text-mining workflow in response to user queries. The orchestrator allocates tasks through a dynamic, context-aware process driven by an LLM rather than by fixed rules or keyword matching. At each turn, the orchestrator's underlying LLM evaluates the entire conversation history along with the predefined roles and capabilities of all available agents. Based on this holistic analysis, it reasons which agent's expertise is most relevant to the current state of the task and selects the most suitable agent to speak next. This model-based approach enables flexible and intelligent task delegation. Based on the open-source framework AutoGen [50] for building AI agent systems, all five agents in eNRRCrew possess reasoning and planning capabilities [19]. Apart from the orchestrator, each agent can perform tool calling to accomplish tasks that users request.

Within this framework, users can interact with the crew through natural language. For example, the crew can predict eNRR yield and FE based on user-submitted information about electrocatalysts and reaction conditions such as applied electrode potential, pH and electrolyte types, as demonstrated in Fig. S29. Users can also chat with the crew to interact with data files like CSVs, gaining insights into quantitative trends of eNRR research. Additionally, users can engage in discussion with the crew to understand eNRR research landscapes. Unlike typical chatbots, the responses provided by the crew are all evidence-based, and users can also access the references cited by the agents for further research.

Next, we will present a few examples to demonstrate how eNRRCrew can assist researchers in understanding the current state of eNRR research and facilitate the rational design of catalysts. As shown in Fig. 5c, the user wanted to explore whether there is a relationship between FE and the corresponding applied electrode potential. In response to this query, the orchestrator in eNRRCrew selected the next speaker as the CSV file handler based on its understanding of the user's question. The CSV file handler, after considering the user's inquiry, proposed a plan to resolve the issue by creating a scatter plot of the two variables and generating the corresponding Python code. This code was executed by the code executor, automatically producing the plot shown in Fig. 5d. In our setup, if there is a code execution error, the crew allows the code executor to return the error message to the CSV file handler, which can then regenerate the code until the task is successfully completed. This capability enhances the efficiency of the research workflow by minimizing users’ intervention, enabling users to focus on interpreting results rather than troubleshooting technical issues. Additionally, it demonstrates the adaptability of eNRRCrew in addressing complex queries in a dynamic research environment.

Chatbots powered by LLMs represent a transformative advancement in AI, with growing applications across various domains due to their ability to generate human-like responses and understand context [51]. However, these systems face significant challenges, particularly in the scientific domain, where accuracy, evidence-based response and comprehensive data understanding are critical. Traditional retrieval-augmented generation (RAG) methods [52], which typically generate answers based on the similarity between user queries and text fragments, are a solution to address the above issues, but often fall short when required to handle complex questions that demand a deeper understanding of large datasets. To address these limitations, eNRRCrew incorporates a GraphRAG retriever that utilizes the LLM to construct a knowledge graph capable of integrating information across multiple text blocks, as shown in Fig. 5b. This enables the system to answer users’ queries based on a comprehensive understanding of large datasets. For example, as shown in Fig. 5e, when a user asks which catalysts exhibit a high yield per unit mass in the eNRR, the orchestrator assigns the query to the GraphRAG retriever, which retrieves pertinent data from a knowledge graph to formulate a detailed response. The answer emphasizes the high NH_3_ yield rates of various catalysts, such as the 3D COFs with Fe–N_4_, supported by specific data references. Compared with the answer generated by GPT-4o-mini, as shown in Fig. S27, the response generated by eNRRCrew is more targeted, scientifically rigorous and data-driven. Additionally, users can refine their queries by utilizing local search and global search options to choose between more precise or broader information. Furthermore, by selecting the desired content type, users can customize the length of the response based on their specific requirements.

**Figure 6.**
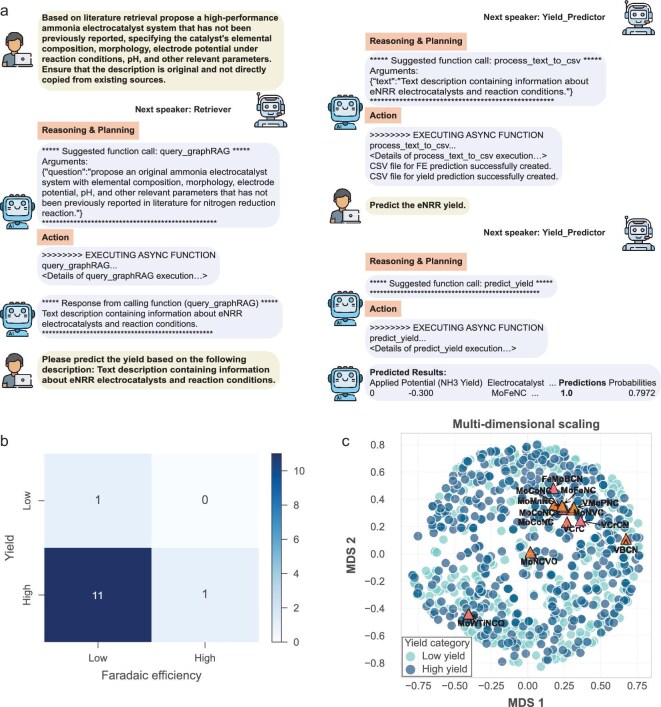
eNRRCrew's capability in proposing and evaluating novel electrocatalysts. (a) Dialogue illustrating eNRRCrew's automated workflow for recommending novel catalyst systems and predicting their performance based on natural language input. (b) Summary of predicted performance (NH_3_ yield vs FE) for 13 catalyst systems proposed by eNRRCrew. (c) MDS plot visualizing the relationship between eNRRCrew's 13 recommended catalysts (triangles) and existing database catalysts (circles), based on their eNRR yield.

Beyond information retrieval and analysis, eNRRCrew is engineered to proactively contribute to catalyst discovery by recommending and predicting the performance of novel electrocatalyst systems. This capability leverages the structured knowledge within its knowledge graph database, which contains detailed information about existing catalysts, reaction conditions, performance metrics and the intricate relationships between these entities. As depicted in Fig. 6a, users can engage in dialogue with eNRRCrew to request recommendations for novel catalyst systems. The crew automatically executes relevant tools to transform user-provided text information into CSV files usable by the Yield Predictor. The crew then executes this function asynchronously, automating the processes of data preprocessing, feature engineering and predictive model execution, enabling end-to-end catalyst recommendation and performance prediction through natural language. To evaluate the efficacy of this recommendation feature, we conducted 10 independent task executions. The results, summarized in Fig. 6b, show that these 10 runs yielded 13 unique catalyst system recommendations, and detailed recommended electrocatalysts and reaction conditions are shown in Section 14 in the Supplementary data. Of these, the crew anticipated that 11 systems would exhibit high NH_3_ yield but low FE. One system was predicted to have both low yield and low FE, while specifically, one system was identified as a candidate for achieving both high yield and high FE, which is the Mo–W dimer on Ti_2_NO_2_ MXene. To directly probe the viability of this novel candidate, we performed *ab initio* molecular dynamics (AIMD) simulations. Our results confirmed its high thermodynamic stability, providing crucial computational validation for eNRRCrew's top prediction. A comprehensive analysis, including detailed stability results and a plausible synthesis pathway, is provided in Section 15 of the Supplementary data. Figure 6c further illustrates the potential of these recommendations through a multi-dimensional scaling (MDS) plot. This plot visualizes the clustering of the 13 newly recommended catalyst systems (represented by triangles) in relation to the existing catalyst systems within our curated database (represented by circles), based on their eNRR yield (categorized as high or low yield). Notably, the recommended systems predicted to have high yield predominantly cluster with the known high-yield catalysts from the database (darker circles). This co-clustering provides compelling evidence for the validity and effectiveness of our trained performance prediction model in identifying promising new catalyst candidates, as well as the rationale for using eNRRCrew for novel catalyst recommendations. Encouragingly, one of the catalysts recommended by eNRRCrew, MoFeNC [single-atom molybdenum (Mo) and iron (Fe) atoms co-anchored on a nitrogen-doped porous carbon matrix], has recently been experimentally validated by our group, demonstrating promising eNRR activity [53]. This validation underscores the potential of eNRRCrew not only to organize existing knowledge but also to generate novel, experimentally verifiable hypotheses, thereby accelerating the discovery cycle for advanced eNRR electrocatalysts.

eNRRCrew demonstrates practical utility through its versatile prediction capabilities for both eNRR yield and FE. When tested against six recent publications from Google Scholar featuring novel electrocatalysts, the system successfully processed catalyst composition, properties and reaction parameters to generate reliable predictions. The yield prediction achieved an F1 score of 0.67, while the FE prediction effectively differentiated between high-performing catalysts (median FE: 22.5%) and lower-performing ones (median FE: 16.14%). These results validate eNRRCrew's effectiveness as a practical tool for catalyst evaluation and optimization in real-world research scenarios. Further details are shown in Figs S30 and S31. By bridging the gap between extensive data and actionable insights, eNRRCrew addresses the limitations of traditional methods, making it a valuable tool for advancing research in fields like electrocatalysis and beyond. As illustrated in Section 16 in the Supplementary data, we developed a system called OERCrew designed for studying the structure–activity relationships in the oxygen evolution reaction (OER) [54], to demonstrate the scalability and inherent modularity of the proposed framework.

### Limitations and future outlook

A limitation of our current framework is its reliance on abstracts for data extraction. While this enables high-throughput analysis, abstracts may omit granular experimental details. However, the common practice in the eNRR field of reporting key performance metrics in abstracts, combined with our model's successful predictions, validates the utility of this approach for building effective foundational models. Our scaling analysis, as shown in Fig. S32, further confirms that though our current dataset is effective, model performance consistently improves with more data, highlighting a clear path for future enhancement. Looking forward, several frontiers are crucial for advancing data-driven catalyst design. Extending our pipeline to full-text and multimodal data (e.g. figures and tables) will capture more nuanced features [55]. Furthermore, integrating quantitative stability metrics is essential, though this is currently challenged by a field-wide scarcity of standardized, machine-readable data and a publication bias against negative results. Overcoming these limitations will require tighter integration of data-driven modeling with high-throughput experiments and community-wide adoption of rigorous data reporting standards [56]. As the field matures and these data challenges are met, transitioning to regression-based models will be crucial. Such models will enable the fine-grained prediction of catalyst performance, which is essential for optimizing state-of-the-art materials rather than simply identifying promising candidates. Ultimately, we envisage an autonomous discovery platform that uses active learning to iteratively refine its models, accelerating the design of next-generation electrocatalysts. This study represents a foundational step toward this ambitious goal.

## CONCLUSIONS

In this work, we introduced eNRRCrew, a multi-agent collaboration framework leveraging LLMs and machine learning to address challenges in eNRR research. The framework empowers researchers to extract, analyze and predict structure–activity relationships with unprecedented efficiency. Notably, eNRRCrew excels in recommending novel electrocatalytic systems and forecasting their performance, facilitated by a seamless natural language interface and automated dataset construction using techniques like ChemPrompt and few-shot prompting. Random forest classifiers effectively predicted eNRR yield, with SHAP analysis identifying critical descriptors such as space group number and electronegativity differences, offering vital insights for electrocatalyst design. Clustering techniques further elucidated factors influencing FE. eNRRCrew signifies an advancement in applying multi-agent LLM frameworks to scientific research. It provides a versatile, scalable platform for accelerating eNRR studies and guiding rational catalyst design, with its methodologies holding promise for broader applications across diverse electrocatalysis domains and other scientific fields.

## METHODS

### Literature corpus collection and analysis

We constructed a comprehensive literature corpus within the field of eNRR by retrieving abstracts from the Web of Science database. A total of 2321 paper abstracts were collected with the targeted search query of ‘electrochemical’ OR ‘electroreduction’ OR ‘electrocatalytic’ OR ‘electro’ (Topic) AND ‘N_2_ reduction’ OR ‘nitrogen reduction’ OR ‘N_2_ fix*’ OR ‘nitrogen fix*’ OR ‘NRR’ (Topic). To identify underlying themes within the literature corpus, we applied LDA with the Gensim library in Python. We employed t-SNE using the scikit-learn library in Python to visualize the clustering of papers based on the topic distribution from the LDA model. The text-embedding-3-small model from OpenAI was used as an embedding model to represent the concepts within the literature corpus and a typical experimental eNRR abstract template, and a cosine function was used to evaluate the similarities.

### Machine-learning model construction

The final dataset used for model construction contained 1117 entries after data cleaning and preprocessing. We allocated 20% of the data to serve as an independent test set, while the remaining 80% constituted our training set. Magpie [31] was employed to develop descriptors associated with the elemental composition of electrocatalysts for eNRR. Structural descriptors of electrocatalysts and reaction condition-related descriptors like pH and electrolyte type were encoded using one-hot encoding. To build a robust and generalizable predictive model, we implemented a rigorous machine-learning protocol. This protocol involved several key steps to mitigate overfitting and ensure the selection of a high-performing model. First, we employed recursive feature elimination with cross-validation (RFECV) to select the most informative subset of features. Next, we considered several machine-learning models, including random forest classifier, support vector classifier, XGBoost classifier and random forest regressor using the scikit-learn library, and artificial neural networks from the TensorFlow library in Python. For each candidate model, we utilized Optuna [37], an automatic hyperparameter optimization framework, to find the optimal hyperparameters for each model. This optimization was performed within a cross-validation loop to prevent overfitting to the training data. A KNN algorithm from the scikit-learn library was used as the baseline model. Following this comprehensive evaluation, the optimized random forest classifier was selected as the best-performing model. To interpret its predictions, we employed SHAP. For the FE dataset, where regression proved ineffective, we applied agglomerative clustering techniques. Specifically, we used hierarchical clustering with Ward's linkage method and selected six clusters based on the dendrogram. For the interpretation of the FE prediction model, we conducted a feature importance analysis based on the standard deviation of feature means across different clusters.

### Details of eNRRCrew

eNRRCrew is a multi-agent collaboration framework for eNRR research, primarily built upon AutoGen [50]. Users interact with the system through a user interface provided by Chainlit. Each agent within eNRRCrew is driven by LLMs. Specifically, the orchestrator is based on GPT-4o, while the other agents executing specific tasks are driven by GPT-4o-mini. In addition to the agents described in the main text, there is also a code execution agent. The code execution agent can interact with the CSV file handler, yield predictor and FE predictor to complete complex tasks by executing code in a Docker image. The yield predictor and FE predictor, based on pretrained models in the former section, can automatically perform data preprocessing, feature engineering and model prediction. The GraphRAG retriever provides evidence-based responses by invoking GraphRAG queries as functions, and is also capable of recommending novel catalysts. The knowledge graph, which underpins the GraphRAG retriever agent, is constructed from structured tabular data with a multi-step, automated pipeline. This process begins with the ingestion of text from the source data, which is then chunked and processed by an LLM to perform entity and relationship extraction based on a predefined schema relevant to eNRR. The pipeline includes sophisticated mechanisms for entity disambiguation, description summarization and efficient incremental updates to ensure the graph remains current and coherent. A detailed description of this methodology, including the graph schema, data storage architecture and entity management strategies, is provided in Section 13 of the Supplementary data.

## Supplementary Material

nwaf372_Supplemental_File

## Data Availability

The eNRRCrew library is available on GitHub (https://github.com/nkuhuxu/eNRRCrew). In addition to the source code, the complete dataset of structure–activity relationships extracted from the 2321 analyzed papers is also provided in this repository. An online demo of eNRRCrew is available at https://enrrcrew.streamlit.app/ (Note: The application may take a moment to load as it wakes from a sleep state).
