# Supplementary material for: Automating structure–activity analysis for electrochemical nitrogen reduction catalyst design through multi-agent collaborations
Source: Natl Sci Rev. 2025 Sep 4;12(11):nwaf372. doi: 10.1093/nsr/nwaf372 (PMC12586994; doi:10.1093/nsr/nwaf372)
Supplement: nwaf372_Supplemental_File [file nwaf372_supplemental_file.pdf]

Supplementary data of

Automating structure-activity analysis for electrochemical nitrogen reduction catalyst design through multi-agent collaborations

Xu Hu [1], Suya Chen [1], Letian Chen [1], Huijuan Wang[1], Xu Zhang[2,3]*, Zhen Zhou[1,2]*

[1] School of Materials Science and Engineering, Institute of New Energy Material Chemistry, Renewable Energy Conversion and Storage Center (ReCast), Key Laboratory of Advanced Energy Materials Chemistry (Ministry of Education), Nankai University, Tianjin 300350, China

[2] Interdisciplinary Research Center for Sustainable Energy Science and Engineering (IRC4SE^2), School of Chemical Engineering, Zhengzhou University, Zhengzhou 450001, Henan, China

[3] Longmen Laboratory, Luoyang 471023, Henan, China

* Corresponding author. E-mail: zzuzhangxu@zzu.edu.cn; zhouzhen@nankai.edu.cn

1. Benchmarking LLMs on eNRR Parameter Extraction

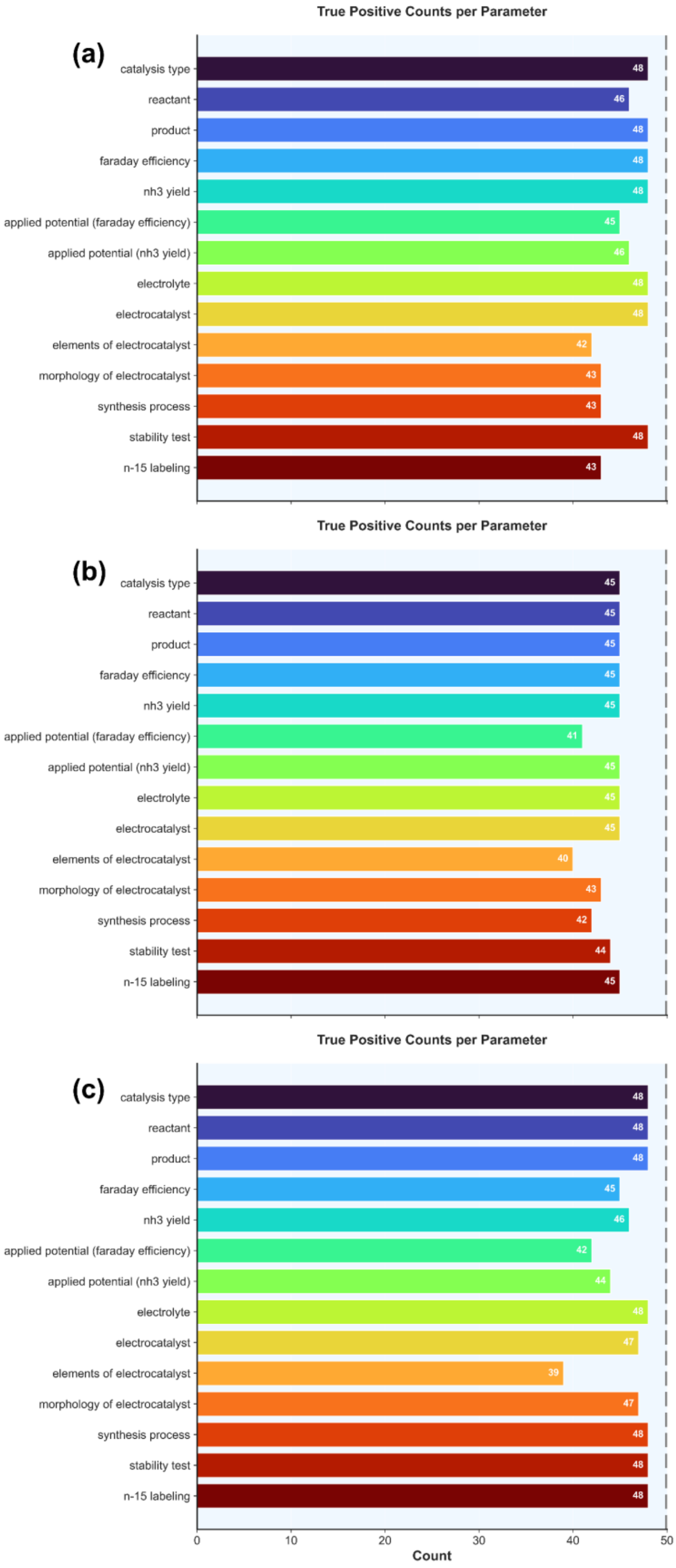

Figure S1. Distribution of true positive counts for each of the 14 key ammonia synthesis

parameters based on randomly selected 50 abstracts for (a) GPT-4o, (b) DeepSeek-v3, and (c) DeepSeek-R1. For the three models, notwithstanding slight variations, the extracted counts exhibit a high degree of concordance, underscoring the models' competence in accurately identifying the specified parameters. TP, correct identification of parameters.

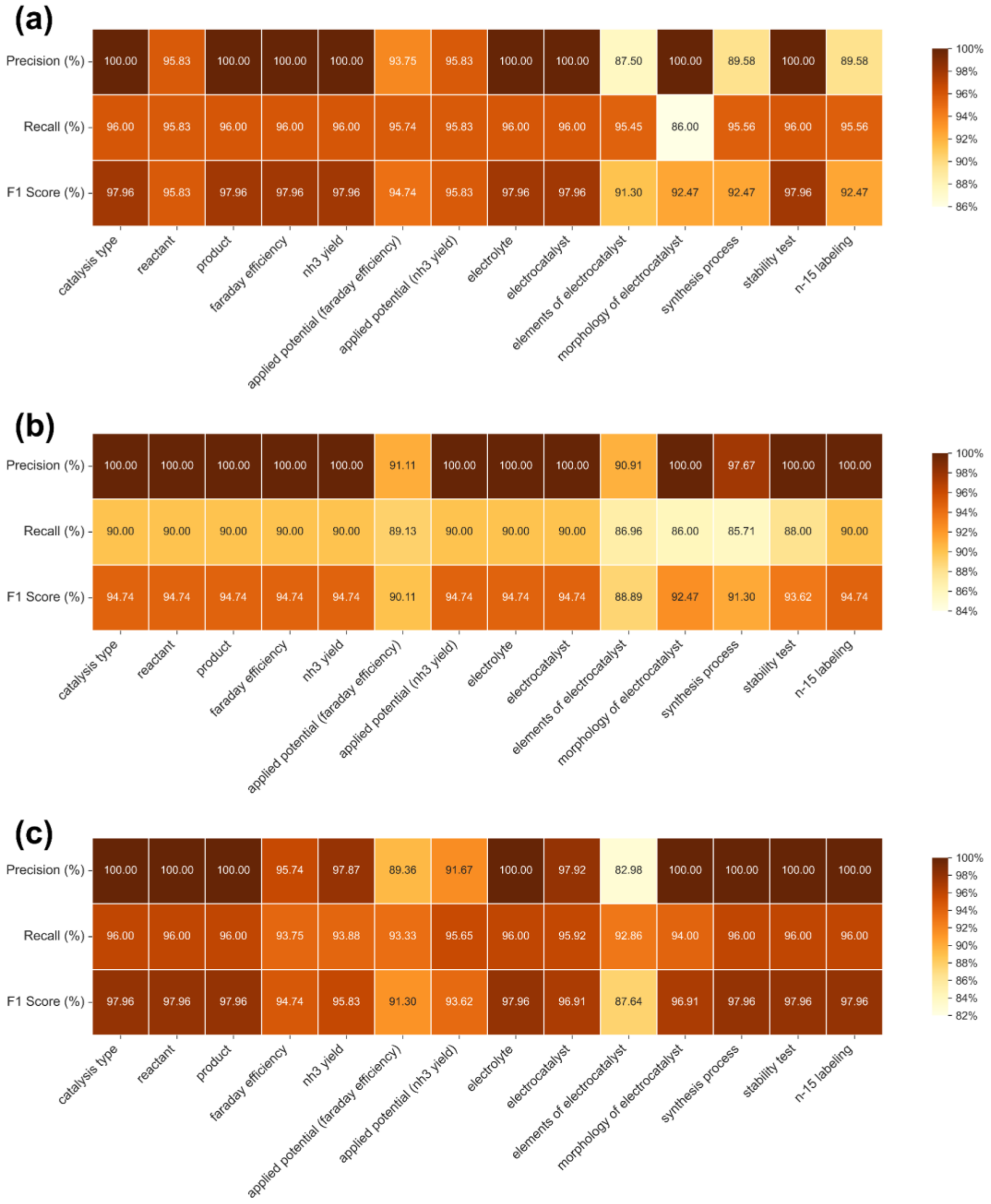

Figure S2. A heat map presenting the granular percentage values of precision, recall, and F1 scores for each synthesis parameter for (a) GPT-4o, (b) DeepSeek-v3, and (c) DeepSeek-R1. TP, correct identification of parameters; FP, incorrect identification of parameters or extraction of irrelevant information; FN, failure to extract relevant parameters.

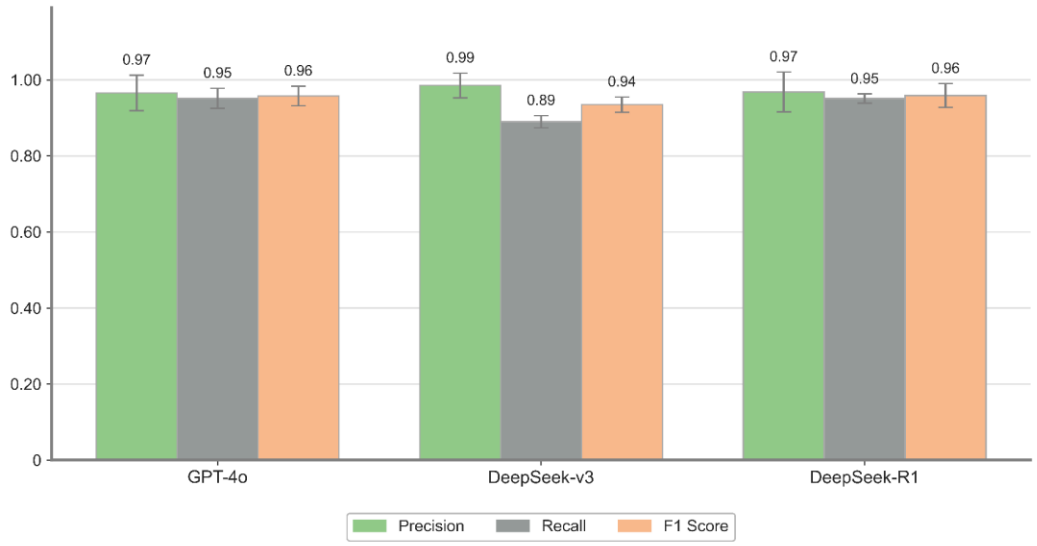

Figure S3. Composite mean precision, recall, and F1 scores for (a) GPT-4o, (b) DeepSeek-v3, and (c) DeepSeek-R1. The gray error bars depicted in the visualization represent the standard deviations of the respective metrics. TP, correct identification of parameters; FP, incorrect identification of parameters or extraction of irrelevant information; FN, failure to extract relevant parameters.

2. Analysis of Elemental Composition in the eNRR Catalyst Dataset

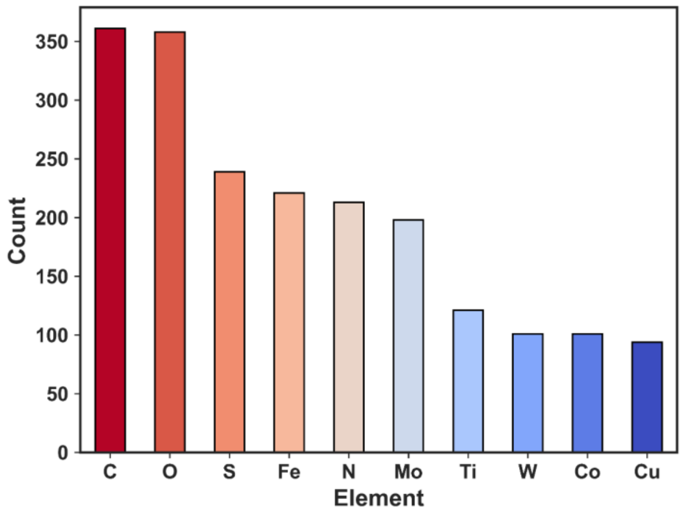

Figure S4. Frequency distribution of top 10 most common individual elements in the collected eNRR catalyst dataset. The analysis is based on 1,837 data entries extracted from the literature. The y-axis indicates the count of publications in which the element was reported as part of the catalyst composition.

To provide a comprehensive overview of the element space covered by our dataset, we analyzed the elemental composition of the catalysts reported in the 1,837 literature entries. Figure S4 shows the frequency of the top 10 most reported elements. carbon (C) and oxygen (O) are the most prevalent elements. This is largely attributable to the widespread use of carbon-based materials (e.g., carbon cloth, paper and nanotubes) as conductive supports and the common occurrence of catalysts in their oxide or hydroxide forms. Among the catalytically active metallic elements, iron (Fe) and molybdenum (Mo) are the two most frequently studied metal elements, appearing in 221 and 198 entries, respectively. This finding is noteworthy as it mirrors the composition of biological nitrogenase enzymes, which utilize Fe- and Mo-based cofactors for nitrogen fixation in nature. This suggests that the research community has historically drawn significant inspiration from these biological systems.

3. Development and Analysis of Machine Learning Models for eNRR Yield Prediction

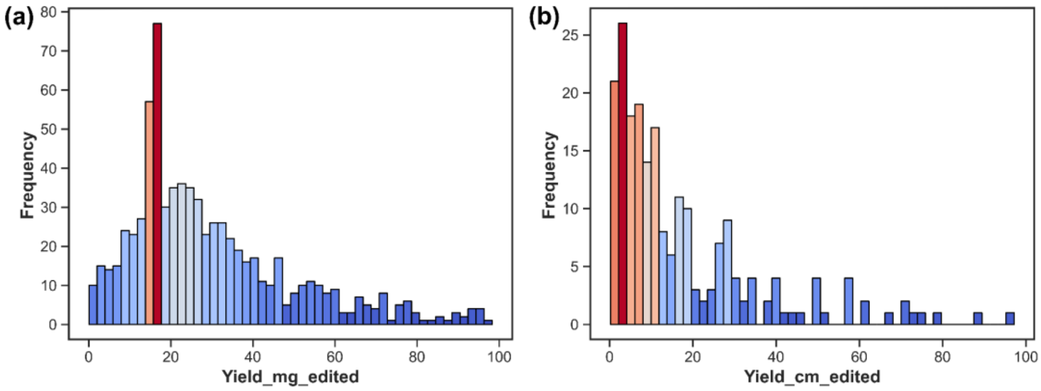

Figure S5. Histograms illustrating the distribution of eNRR yield data. (a) Frequency distribution of yield measured in μg h^{-1} mg_{cat}^{-1} (unit mass of the electrocatalyst). (b) Frequency distribution of yield measured in μg h^{-1} cm^{-2} (unit area of the electrocatalyst). The color gradient indicates the density of occurrences across different yield values.

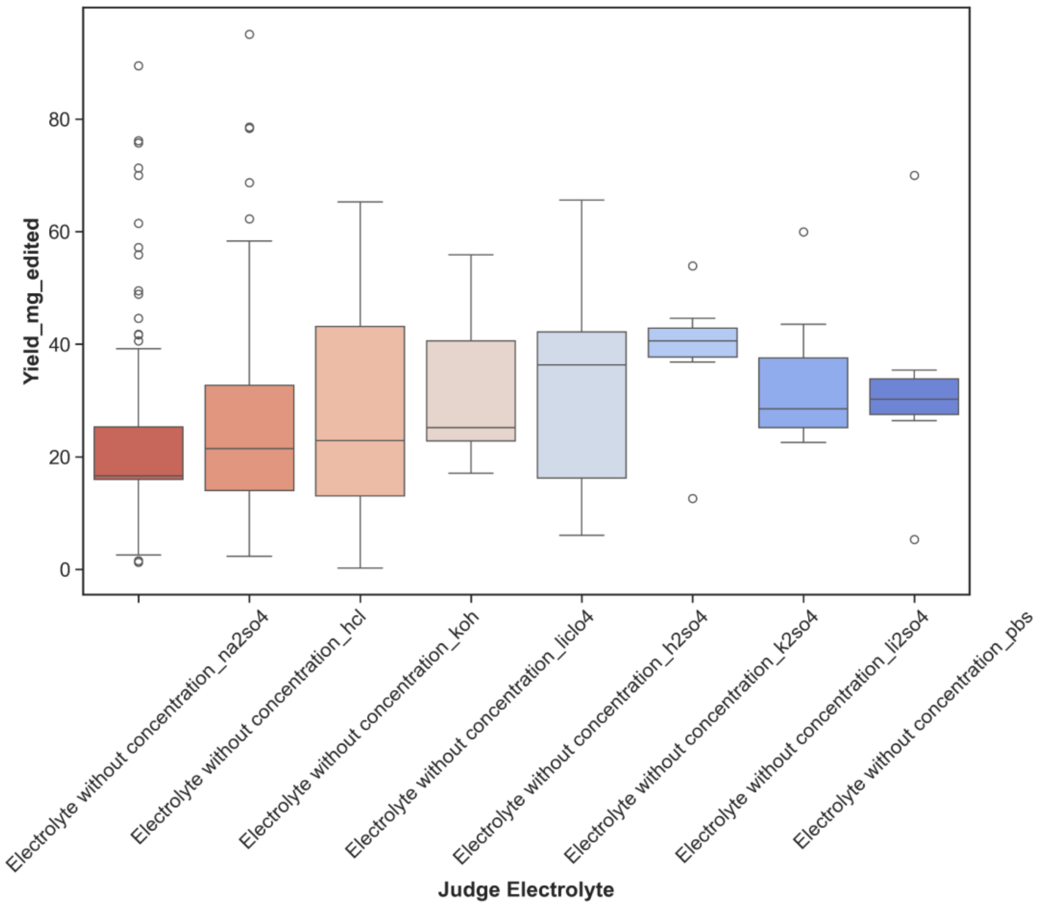

Figure S6. Yield in unit mass distribution by Judge Electrolyte properties. The color gradient indicates the frequency of occurrences across different judge criteria.

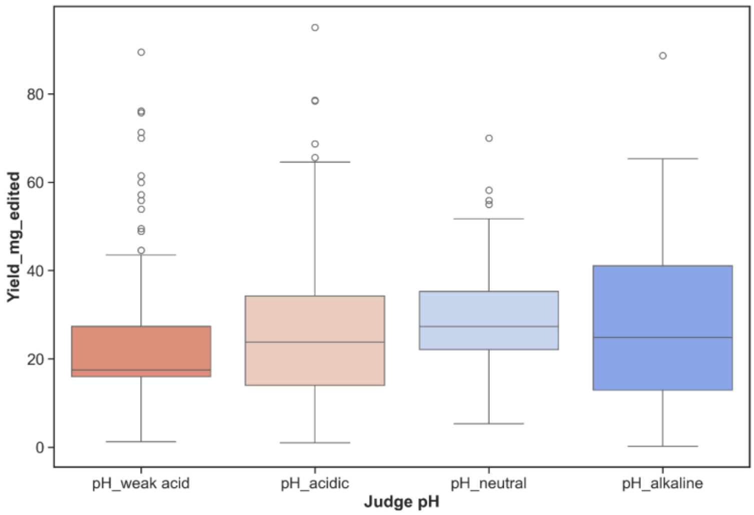

Figure S7. Yield in unit mass distribution by Judge pH properties. The color gradient indicates the frequency of occurrences across different judge criteria.

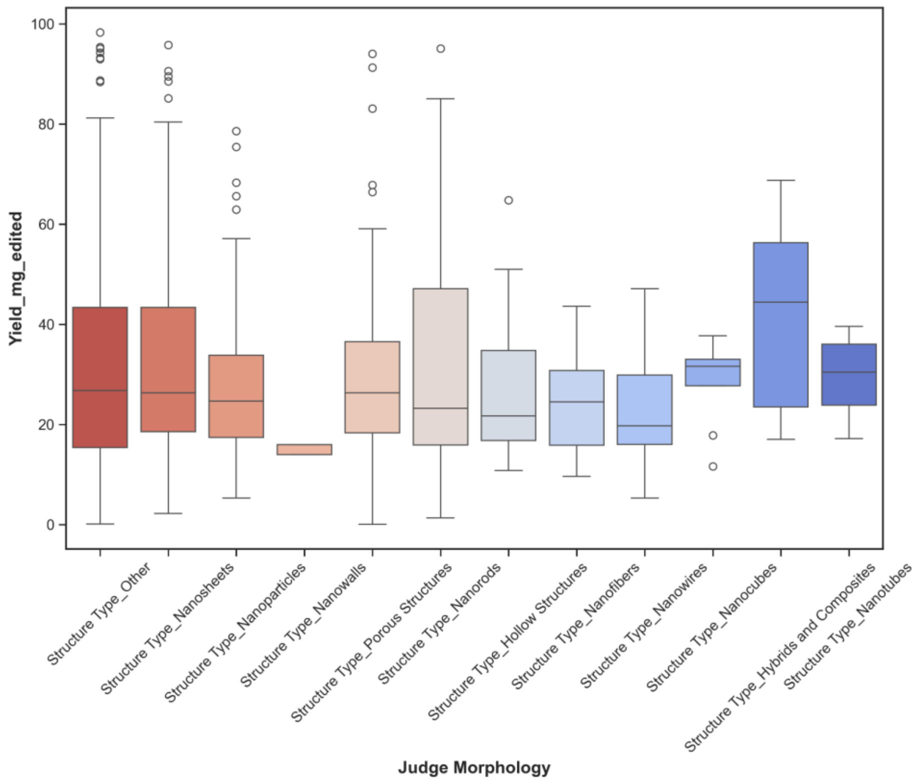

Figure S8. Yield in unit mass distribution by Judge Morphology categories. The color gradient indicates the frequency of occurrences across different judge criteria.

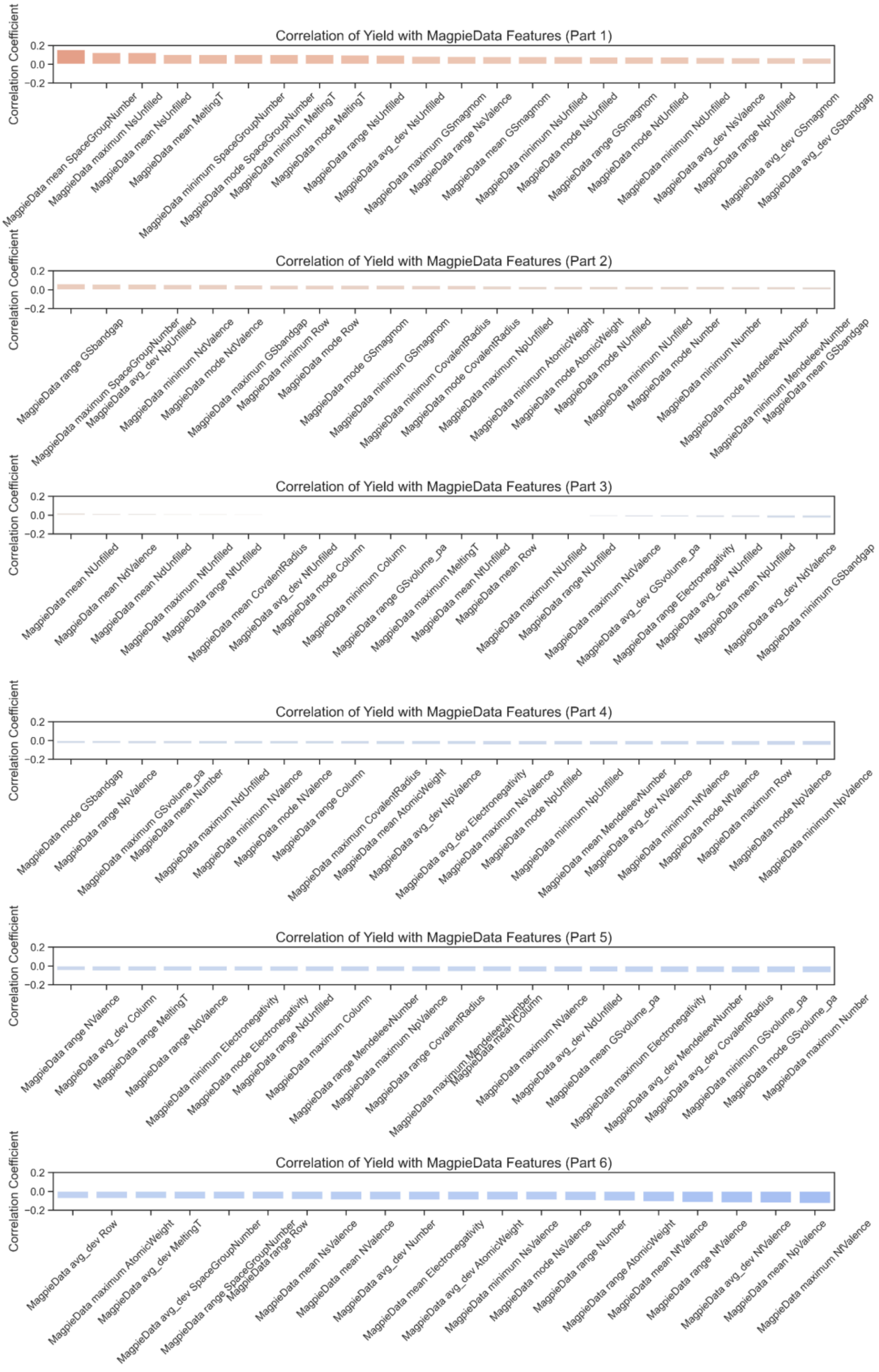

Figure S9. Yield in unit mass distribution by Judge element properties. The color gradient indicates the correlation values.

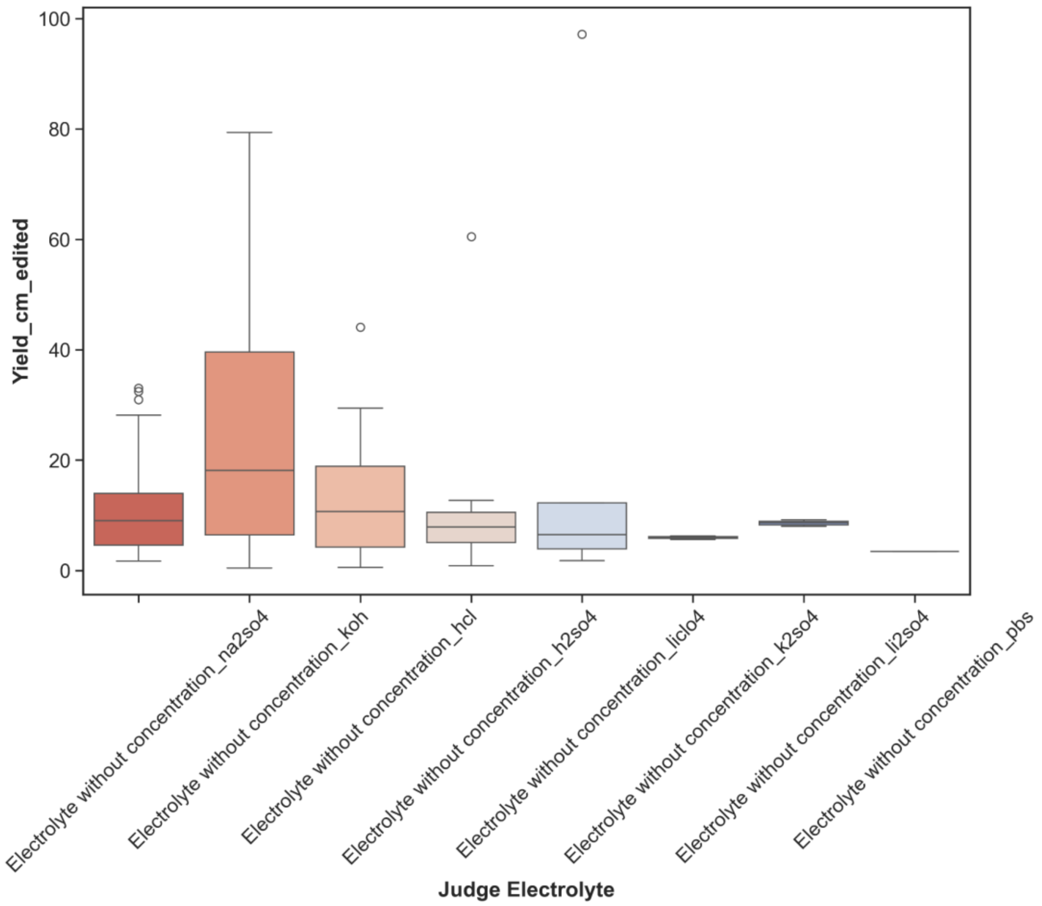

Figure S10. Yield in unit area distribution by Judge Electrolyte properties. The color gradient indicates the frequency of occurrences across different judge criteria.

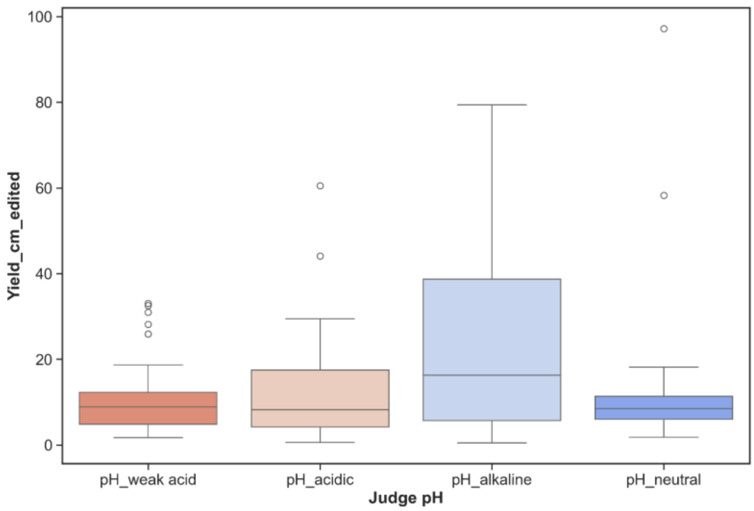

Figure S11. Yield in unit area distribution by Judge pH properties. The color gradient indicates the frequency of occurrences across different judge criteria.

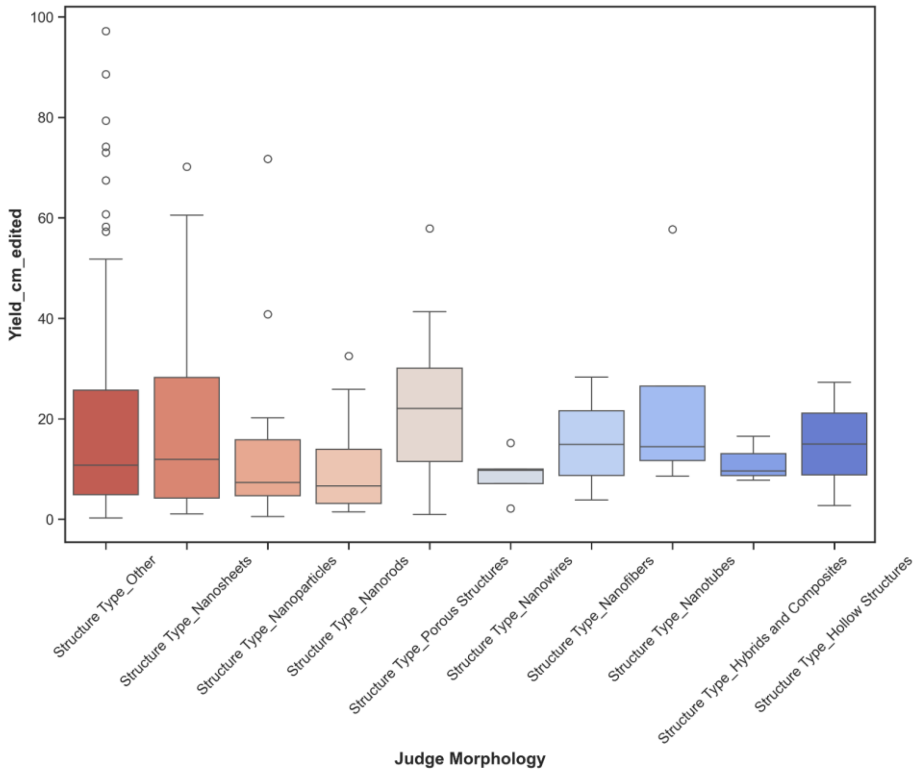

Figure S12. Yield in unit area distribution by Judge Morphology categories. The color gradient indicates the frequency of occurrences across different judge criteria.

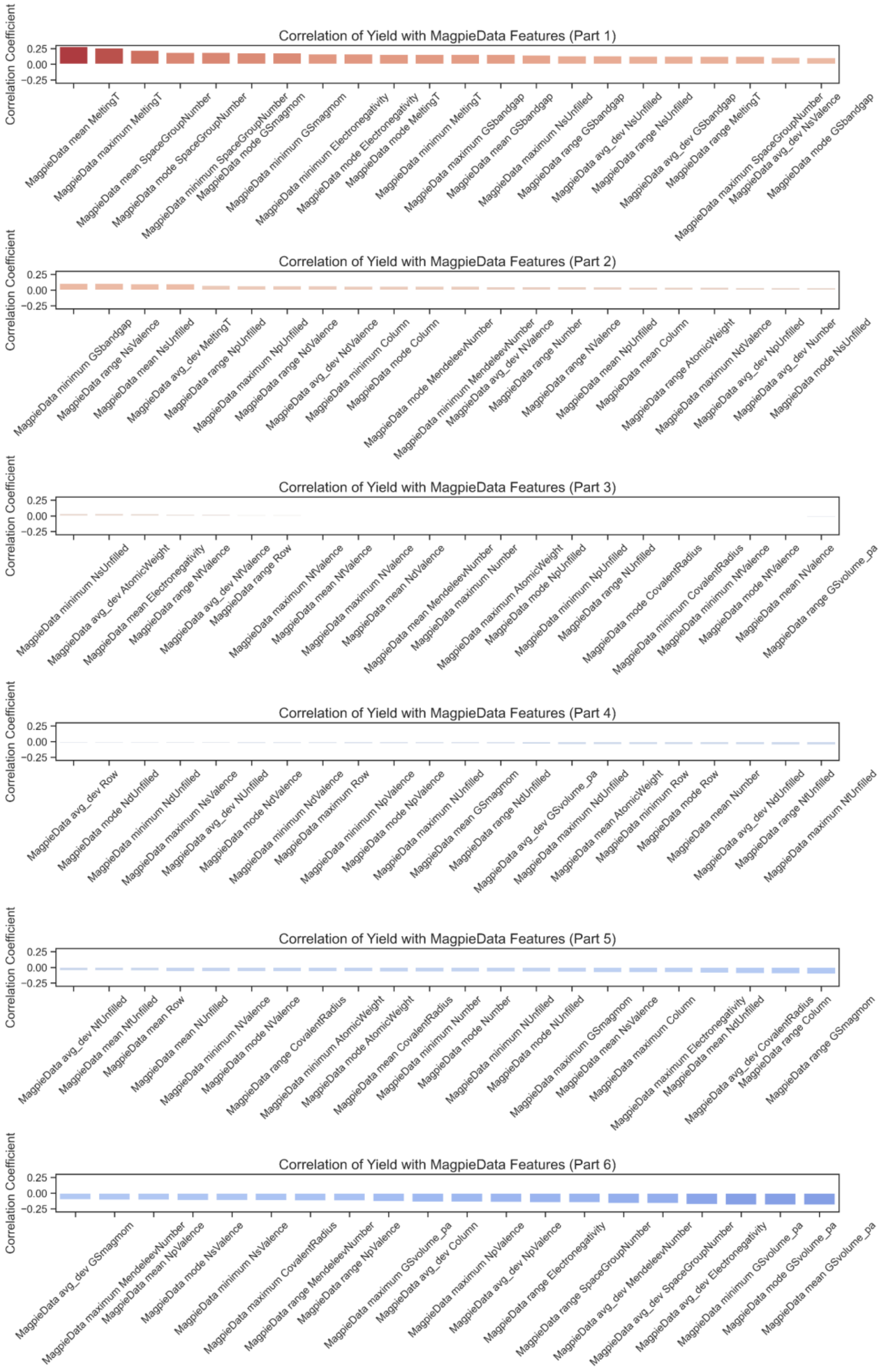

Figure S13. Yield in unit area distribution by Judge element properties. The color gradient indicates the correlation values.

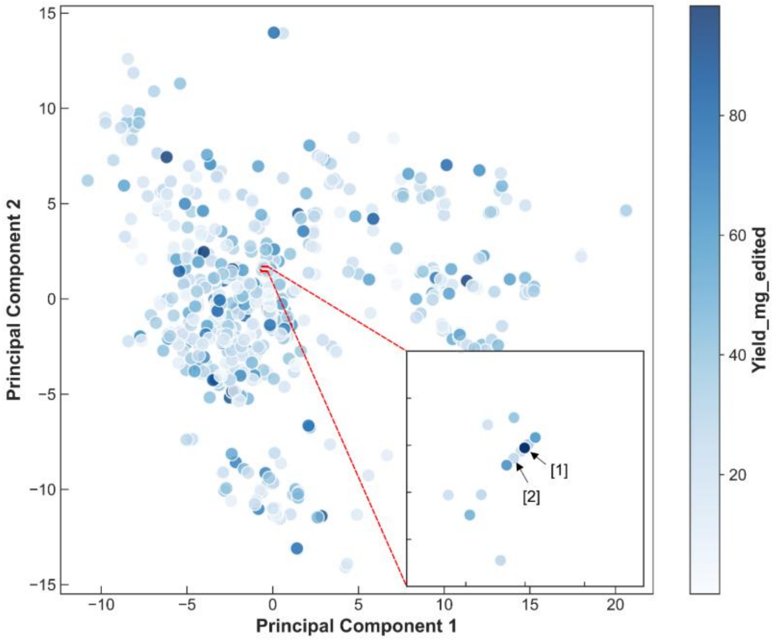

Figure S14. PCA plot visualizing a *reactivity cliff*. Data points are colored by eNRR yield (μg h^{-1} mg_{cat}^{-1}). The inset shows two catalysts with highly similar features but vastly different performance: [1] S-rich 1T-MoS_2 (yield: 98.30)[1] and [2] sulfur-vacancy defective MoS_2 (yield: 29.55)[2].

To visually demonstrate the impact of *reactivity cliff*[3, 4] on our dataset and to further justify our choice of a classification model for eNRR yield prediction, we performed a Principal Component Analysis (PCA) on the subset of data for which yields were reported in μg h^{-1} mg_{cat}^{-1} (Figure S14). The PCA plot maps the high-dimensional feature space onto a two-dimensional plane, allowing for the observation of similarity between data points. In the magnified inset of the figure, we highlight two data points, [1] and [2]. Their close proximity in the PCA space indicates that their overall feature vectors are highly similar. However, their catalytic performance is drastically different. Data Point [1] represents the S-rich 1T-MoS_2 catalyst, achieving a high yield of 98.30 μg h^{-1} mg_{cat}^{-1}. Data Point [2] represents the sulfur-vacancy defective MoS_2 catalyst, with a much lower yield of 29.55 μg h^{-1} mg_{cat}^{-1}. This is a classic example of a *reactivity cliff*: a subtle change in the catalyst's composition—specifically, a difference in sulfur content—leads to a discontinuous, "cliff-like" drop in yield of more than threefold. A traditional regression model would attempt to smoothly interpolate the yield in the space between points [1] and [2]. This would not only result in large prediction errors but would also mistakenly guide researchers to explore a smooth structure-activity relationship that does not exist in reality. In contrast, a classification model, which aims to learn a decision boundary, can more robustly partition these two points into high-yield and low-yield classes. This approach avoids making misleadingly precise predictions in these highly non-linear regions, thereby providing more reliable guidance for catalyst screening.

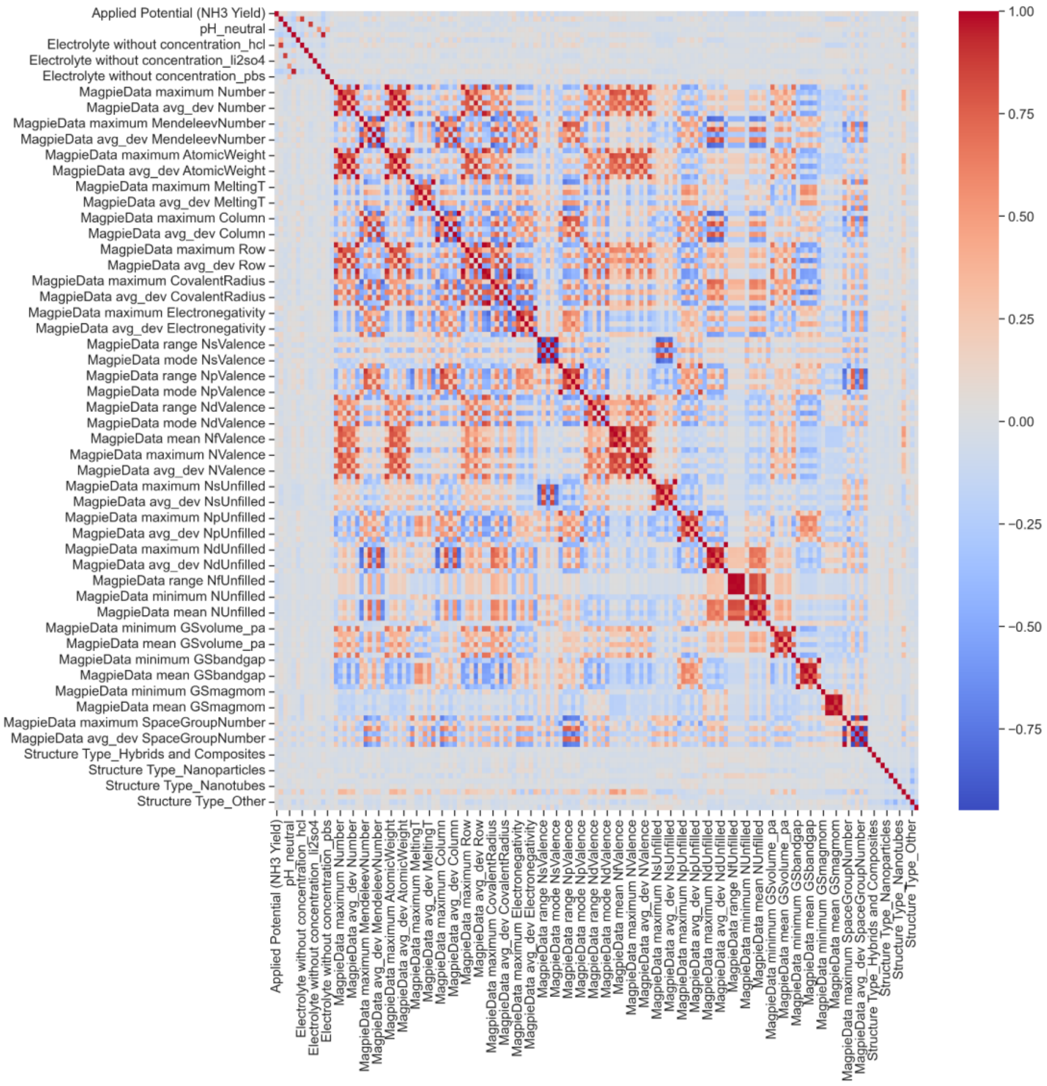

Figure S15. The correlation coefficient matrix of the features retained after recursive feature elimination for predicting eNRR yield.

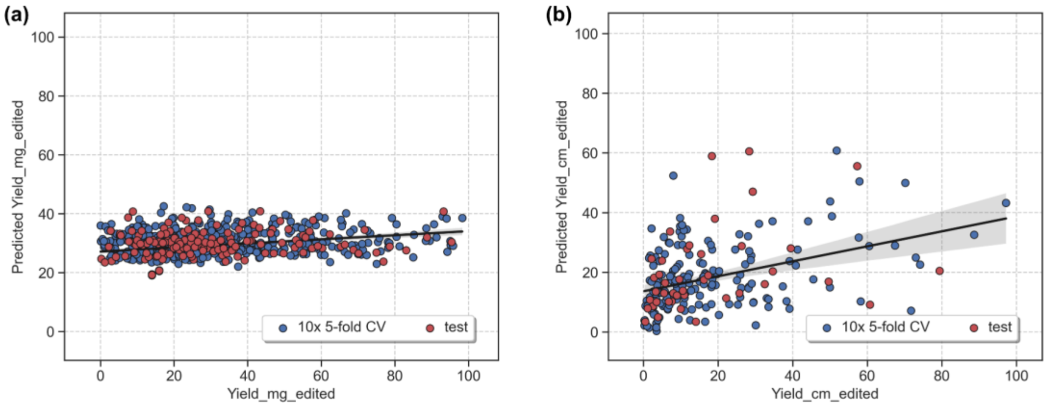

Figure S16. Prediction performance of machine learning models using features selected by permutation feature importance using ROBERT program. The scatter plots show predicted versus actual eNRR yield values for both the 10-times-repeated 5-fold cross-validation set (10×5-fold

CV, blue dots) and the test set (red dots). (a) Predictions for the data_mg_edited dataset using a Gradient Boosting model. The performance metrics were: 10×5-fold CV (R^2 = 0.07, MAE = 14.0, RMSE = 19.0) and test set (R^2 = 0.015, MAE = 15.0, RMSE = 20.0). (b) Predictions for the data_cm_edited dataset using a Gradient Boosting model. The performance metrics were: 10×5-fold CV (R^2 = 0.14, MAE = 13.0, RMSE = 18.0) and test set (R^2 = 0.11, MAE = 14.0, RMSE = 19.0).

To provide a robust empirical basis for our choice of a classification framework, we conducted a systematic investigation into the feasibility of directly predicting the continuous eNRR yield values. To ensure a thorough and unbiased assessment, we employed the ROBERT program (https://robert.readthedocs.io)[5], an automated machine learning framework that supports regression and classification tasks. This tool systematically evaluates various algorithms and hyperparameters to identify the best possible predictive model for a given dataset. We applied this methodology to both the mass-normalized (Yield_mg_edited, unit: μg h^{-1} mg_{cat}^{-1}) and area-normalized (Yield_cm_edited, unit: μg h^{-1} cm^{-2}) subsets of our data. The performance of the best regression models identified by ROBERT is visualized in Figure S16.

The results from this regression analysis were unequivocally poor, confirming our initial hypothesis about the difficulty of this task. For the Yield_mg_edited dataset, the optimal Gradient Boosting model achieved a coefficient of determination (R^2) near zero for both the cross-validation (0.07) and, critically, the test set (0.015). An R^2 value this low indicates that the model has virtually no predictive power and fails to explain any meaningful variance in the data. The scatter plot in Figure S16a visually confirms this; the model's predictions are clustered in a narrow horizontal band, showing almost no correlation with the actual experimental yields. For the Yield_cm_edited dataset, the Gradient Boosting model performed only marginally better, with a test set R^2 of 0.11. While the plot in Figure S16b shows a faint positive trend, an R^2 value around 0.1 still signifies a model that is unsuitable for predictive purposes.

These empirical results strongly support the arguments presented in our main manuscript. The poor performance is consistent with the challenges posed by the literature-mined dataset, including: highly skewed, non-normal data distribution, the presence of a sharp reactivity cliff as shown in Figure S14. Therefore, we chose a machine learning classification model to predict the yield.

Here are the commands for running the ROBERT on the respective datasets: For the Yield_mg_edited dataset: *python -m robert --csv_name "data_mg_yield.csv" --y "Yield_mg_edited" --names "Name" --ignore "['Name']" --corr_filter_x "False" --desc_thres "5" --model "['AdaB','GB','GP','MVL','NN','RF']" --error_type "r2" --shap_show "20" --pfi_show "20"*. For the Yield_cm_edited dataset: *python -m robert --csv_name "data_cm_yield.csv" --y "Yield_cm_edited" --names "Name" --ignore "['Name']" --corr_filter_x "False" --desc_thres "5" --model "['AdaB','GB','GP','MVL','NN','RF']" --error_type "r2" --shap_show "20" --pfi_show "20"*.

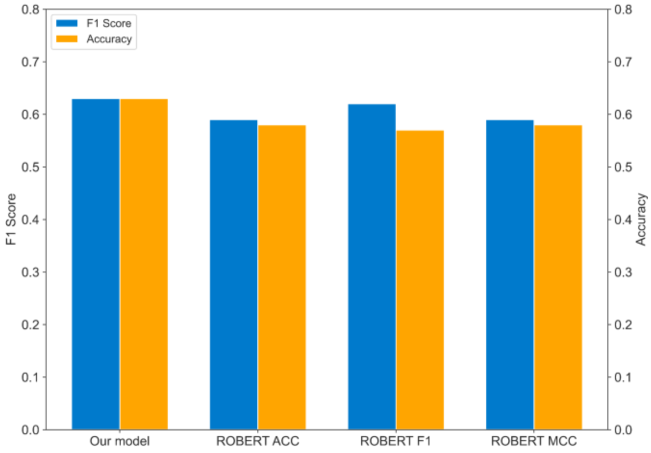

Figure S17. Comparative analysis of predictive models using F1 score and accuracy. This dual-axis bar chart illustrates the performance of our developed classification model against three configurations of the ROBERT model, which were optimized for different error types during hyperparameter optimization (Accuracy, F1 score, and Matthew's correlation coefficient (MCC)). F1 scores are represented by blue bars (left y-axis), while accuracy is shown with orange bars (right y-axis).

The performance of our manually developed model ("Our model") is compared against three models generated by ROBERT, each automatically optimized for a different objective function: Accuracy ("ROBERT ACC"), F1 Score ("ROBERT F1"), and Matthews Correlation Coefficient ("ROBERT MCC"). Bars show the F1 Score (blue, left y-axis) and Accuracy (orange, right y-axis) on the hold-out test set. The results demonstrate that our model's performance is highly competitive and slightly exceeds that of the automated benchmarks, validating the robustness of our modeling protocol and suggesting a performance ceiling inherent to the dataset.

Here are the commands for running the ROBERT on the different objective functions: For accuracy: *python -m robert --csv_name "processed_data.csv" --y "Yield_combined" --names "Name" --type "clas" –ignore "['Name']" --auto_type "False" --seed "49" --kfold "5" --repeat_kfolds "10" --corr_filter_y "True" –model "['AdaB','GB','GP','MVL','NN','RF']" --error_type "acc"*. For F1 score: *python -m robert --csv_name "processed_data.csv" --y "Yield_combined" --names "Name" --type "clas" –ignore "['Name']" --auto_type "False" --seed "49" --kfold "5" --repeat_kfolds "10" --corr_filter_y "True" –model "['AdaB','GB','GP','MVL','NN','RF']" --error_type "f1"*. For MCC: *python -m robert --csv_name "processed_data.csv" --y "Yield_combined" --names "Name" --type "clas" –ignore "['Name']" --auto_type "False" --seed "49" --kfold "5" --repeat_kfolds "10" --corr_filter_y "True" –model "['AdaB','GB','GP','MVL','NN','RF']"*.

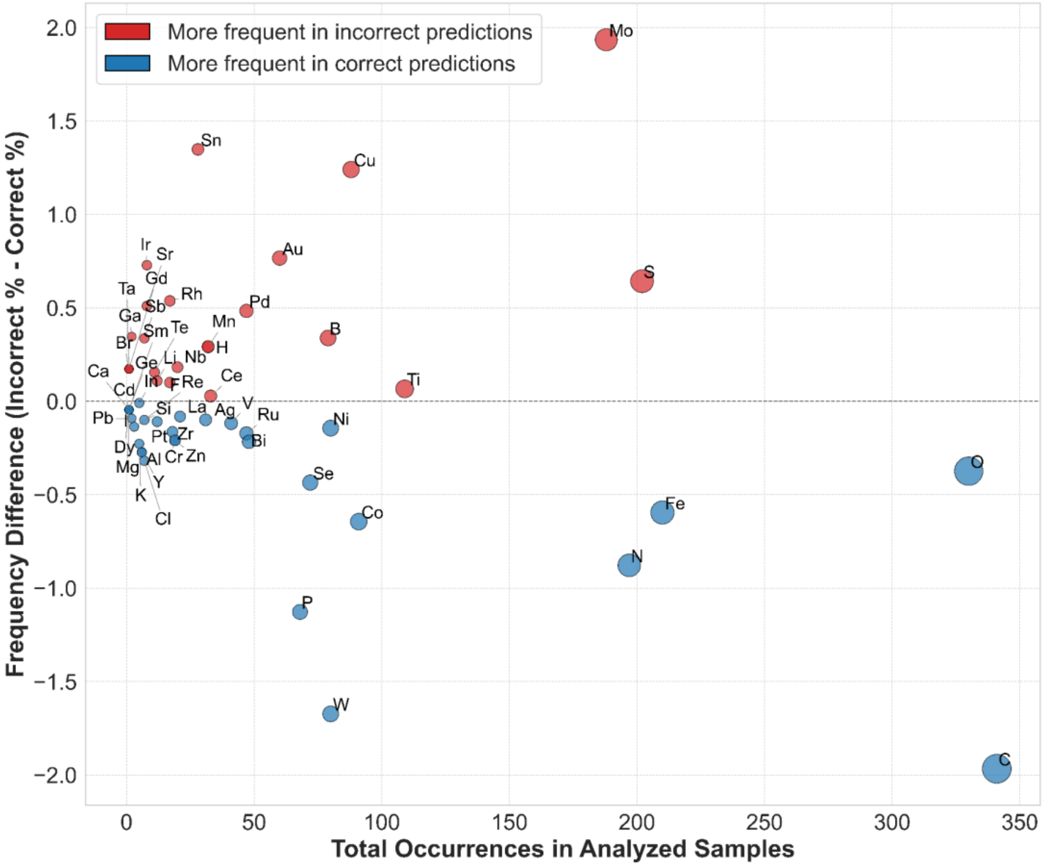

Figure S18. Analysis of elemental contribution to model prediction errors. Each point represents a chemical element. The x-axis shows the total number of occurrences of that element in the dataset (training and test sets combined). The y-axis shows the difference between the element's frequency in incorrectly predicted samples and its frequency in correctly predicted samples (Frequency Difference = Incorrect % - Correct %). A positive value (red points) indicates the element is more frequently associated with misclassifications, while a negative value (blue points) indicates it is more frequently associated with correct predictions. The size of the point is proportional to its total occurrence.

This plot provides a diagnostic map of the model's performance with respect to specific chemical elements, revealing both its strengths and weaknesses:

1. **Elements Associated with Correct Predictions (Blue, Y < 0)**:

   Common and Well-Predicted Elements (Bottom-Right Quadrant): Elements such as Carbon (C), Nitrogen (N), and Iron (Fe) are located in this region. Their high frequency of occurrence and strong association with correct predictions indicate that the model has effectively learned the structure-property relationships for these mainstream catalyst components.

   Less Common but Well-Predicted Elements (Bottom-Left Quadrant): Elements like Cobalt (Co), Phosphorus (P), and Tungsten (W) fall into this category. Despite being less common, the model predicts their impact on eNRR yield with relatively high accuracy. This suggests that these elements may have distinct and more easily learnable electronic or structural features that the model can capture even from limited data.

2. **Elements Associated with Incorrect Predictions (Red, Y > 0)**:
   Common but Poorly-Predicted Elements (Top-Right Quadrant): This quadrant is sparsely populated, but elements like Molybdenum (Mo) and Sulfur (S), which are very common in TMD catalysts, show a tendency to be associated with incorrect predictions. This is a critical finding, suggesting that while these elements are prevalent, their catalytic behavior is highly complex and context-dependent (e.g., sensitive to phase, defects, or morphology), which our current feature set may not fully resolve. This aligns with our *reactivity cliff* case study involving MoS_2 (shown in Figure S14).
   Less Common and Poorly-Predicted Elements (Top-Left Quadrant): This region contains a significant number of elements, including precious metals like Gold (Au), Palladium (Pd), and Iridium (Ir), as well as p-block elements like Tin (Sn). Their association with misclassifications is likely due to their relative scarcity in the dataset. The model has insufficient data to learn the nuanced roles these elements play in eNRR catalysis, leading to higher prediction uncertainty.

This analysis provides clear, actionable directions for future work. The model's primary weaknesses lie in handling catalysts containing less-represented elements (particularly precious metals) and in distinguishing the subtle contextual differences of common but complex elements like Mo and S. Future efforts should therefore focus on two key areas: (1) enriching the dataset with more high quality examples of under-represented but catalytically interesting elements and (2) developing more sophisticated features that can better describe the local chemical environment and phase information for common elements like Mo and S to resolve their complex behavior.

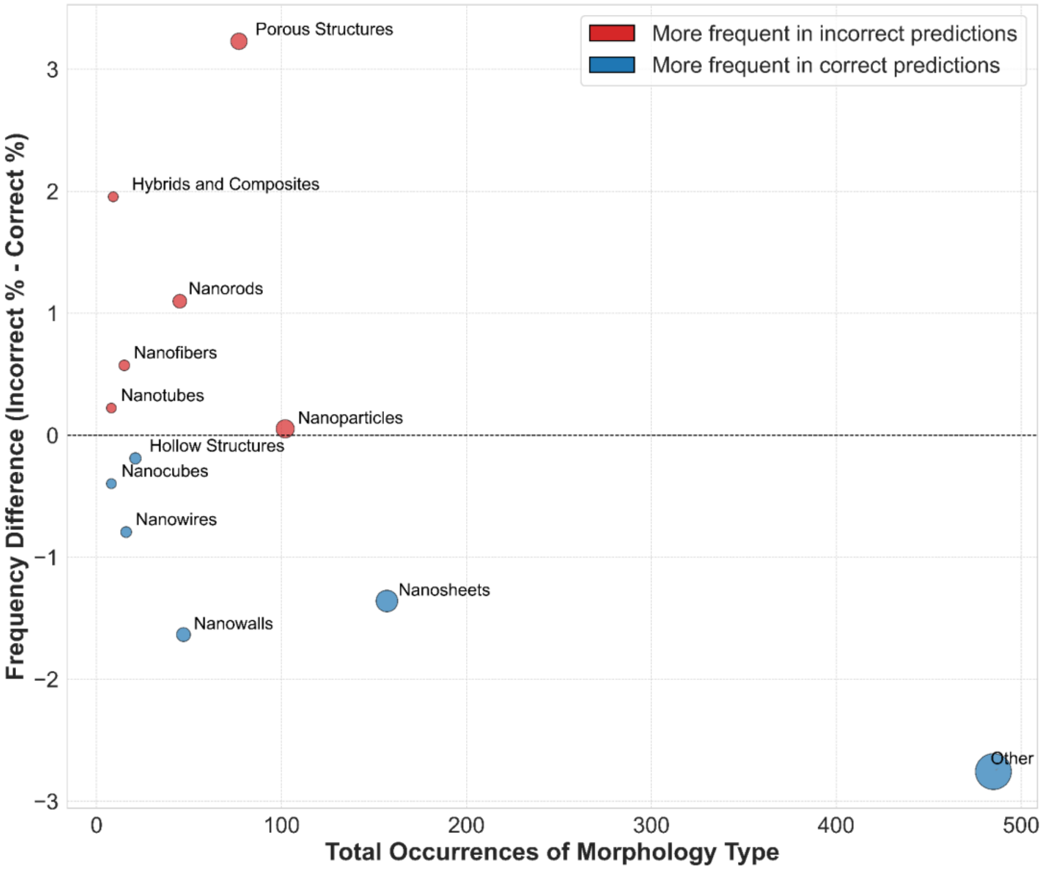

Figure S19. Analysis of catalyst morphology's contribution to model prediction errors. Each point represents a specific morphology type. The x-axis shows the total number of occurrences of that morphology in the dataset. The y-axis shows the difference between the morphology's frequency in incorrectly predicted samples and its frequency in correctly predicted samples (Frequency Difference = Incorrect % - Correct %). A positive value (red points) indicates the morphology is more frequently associated with misclassifications, while a negative value (blue points) indicates it is more frequently associated with correct predictions. The size of the point is proportional to its total occurrence.

This analysis reveals how well the model understands the role of different electrocatalyst morphologies, highlighting specific structural classes that are either well-modeled or challenging for the current feature set.

1. **Morphologies Associated with Correct Predictions (Blue, Y < 0)**:
   Common and Well-Predicted Morphologies (Bottom-Right Quadrant): This region is dominated by the "Other" category, which encompasses a large number of diverse, less-defined structures, and also includes nanosheets. The model's strong performance on these common morphologies suggests it has successfully identified generalizable features that correlate with performance for 2D structures and other frequently encountered forms.
   Less Common but Well-Predicted Morphologies (Bottom-Left Quadrant): Architectures like Nanowalls, Nanocubes, and Nanowires are found here. The model's ability to accurately predict outcomes for these specific, well-defined but less frequent 1D and 3D structures indicates it can effectively capture their distinct structure-activity

relationships.

2. **Morphologies Associated with Incorrect Predictions (Red, Y > 0)**:

   Common but Poorly-Predicted Morphologies (Top-Right Quadrant): Nanoparticles, despite being one of the most common morphologies, are situated near the zero-line, indicating a neutral-to-slightly-negative impact on prediction accuracy. This suggests that "nanoparticle" is a highly general term, and the model struggles to differentiate the performance of various nanoparticles without more specific descriptors (e.g., size distribution, faceting, support interactions).

   Less Common and Poorly-Predicted Morphologies (Top-Left Quadrant): This is the most revealing area. Morphologies like Porous Structures, Hybrids and Composites, Nanorods, and Nanofibers are strongly associated with prediction errors. This is a critical insight: these morphologies are often characterized by complex hierarchical structures, multi-component interfaces, and intricate mass transport properties. The model's difficulty here strongly suggests that our current feature set, while effective for simpler structures, does not adequately capture the complex physics at play in these advanced materials.

The morphological error analysis indicates that the model's primary limitations lie in describing structurally complex systems. While it performs well on well-defined 1D/2D nanostructures, it falters when faced with porous, hybrid, or composite materials. This highlights a crucial direction for future model development: the need to incorporate more sophisticated descriptors that can quantify parameters such as porosity, surface area, component interface area, and tortuosity. Capturing these features is essential for building next-generation models capable of accurately predicting the performance of advanced, hierarchically structured eNRR electrocatalysts.

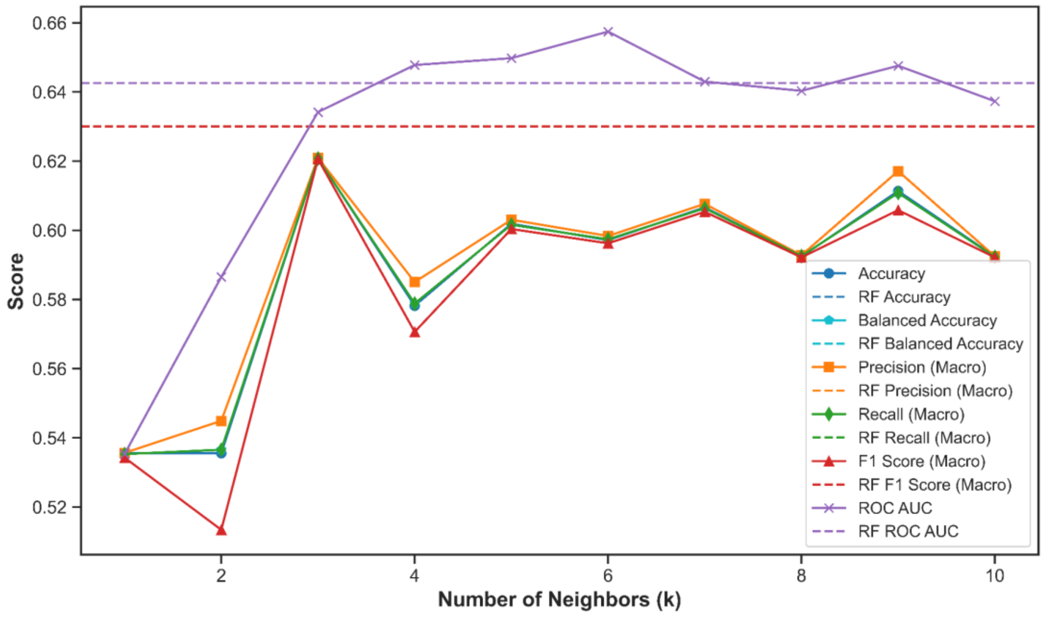

Figure S20. The performance metrics of the KNN model for eNRR yield as a function of the number of neighbors (k).

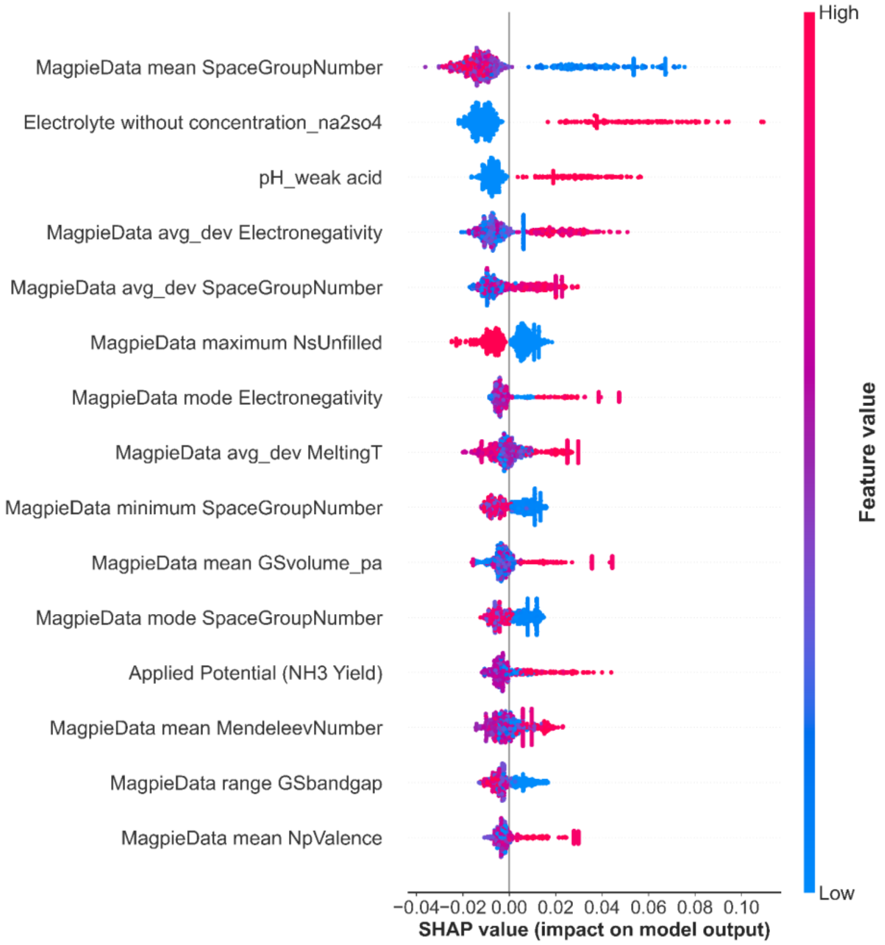

Figure S21. SHAP values illustrate the impact of various features on model output, with features ranked by their influence on yield predictions.

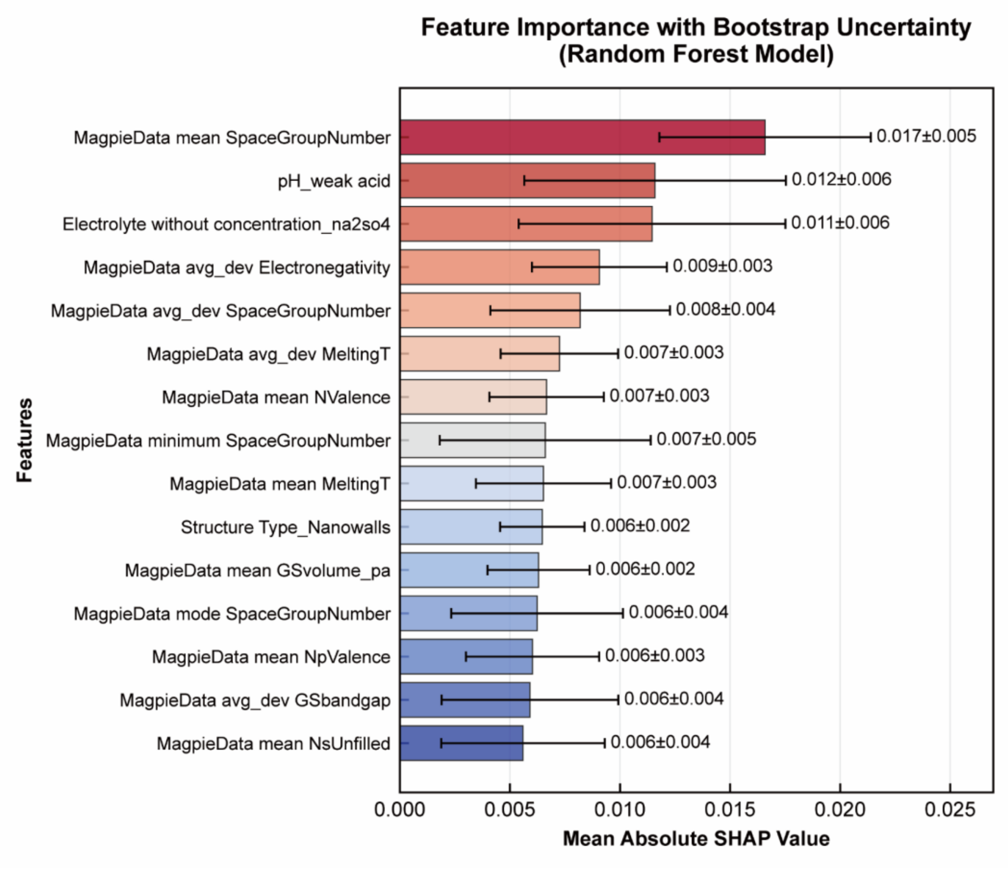

Figure S22. Feature importance with Bootstrap uncertainty for the eNRR yield classifier. The plot shows the mean absolute SHAP values for the most important features identified by the Random Forest model. Uncertainty was quantified by generating 100 bootstrap samples of the dataset. The bars represent the mean importance, and the error bars denote the standard deviation across the bootstrap runs.

This analysis was conducted to validate the stability of the feature importances identified by our model and to address the apparent discrepancy between low linear correlation and high feature importance for certain descriptors. The results in Figure S22 confirm that the top-ranked features, including MagpieData mean SpaceGroupNumber and MagpieData avg_dev Electronegativity, maintain their high importance with statistically significant confidence intervals. This robustly demonstrates that while a feature like avg_dev Electronegativity may have a low linear correlation with yield, its consistently high SHAP value reveals a critical non-linear relationship that was successfully captured by the Random Forest model. This finding underscores the necessity of employing non-linear models and advanced explainability methods to uncover the complex structure-property relationships that govern catalytic performance.

4. Regression Modeling of Faradaic Efficiency and Feature Importance Evaluation

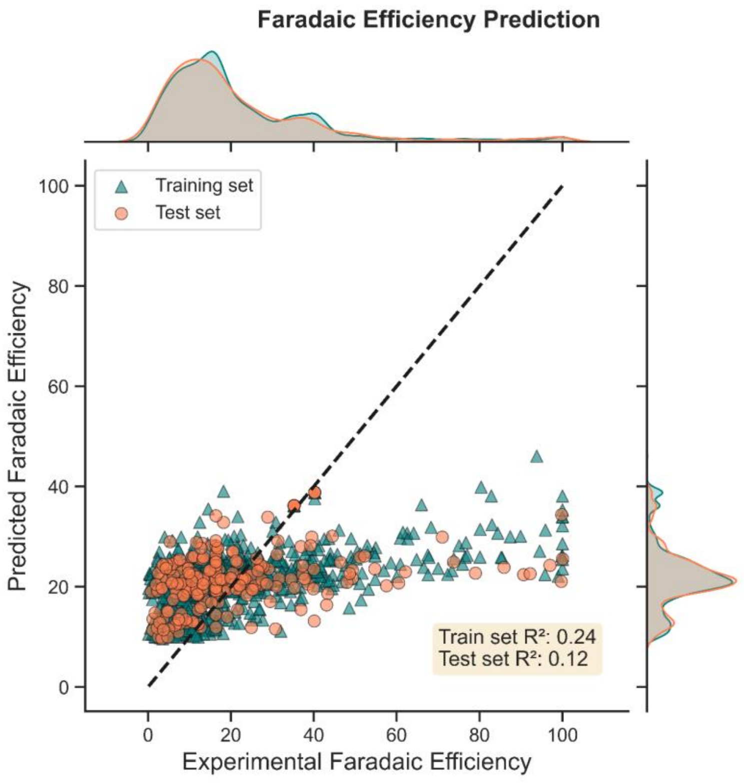

Figure S23. Prediction performance of the Random Forest model for Faradaic efficiency. The parity plot shows the predicted Faradaic efficiency versus the experimental values for both the training set (teal triangles) and the test set (orange circles). The dashed line represents perfect agreement (y=x).

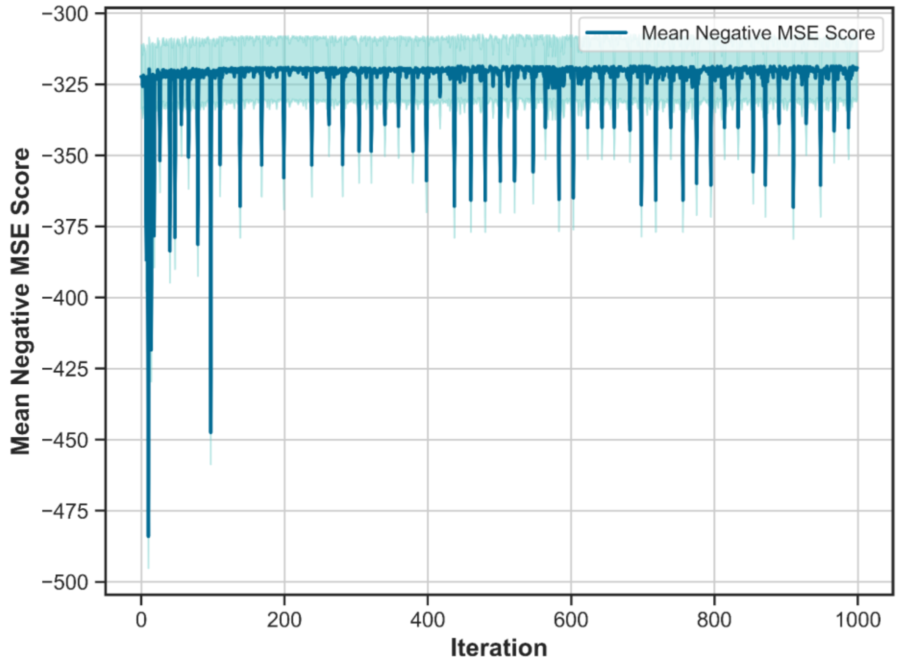

Figure S24. Mean test scores (negative MSE) over iterations during hyperparameter tuning of random forest regression for Faradaic efficiency using Optuna.

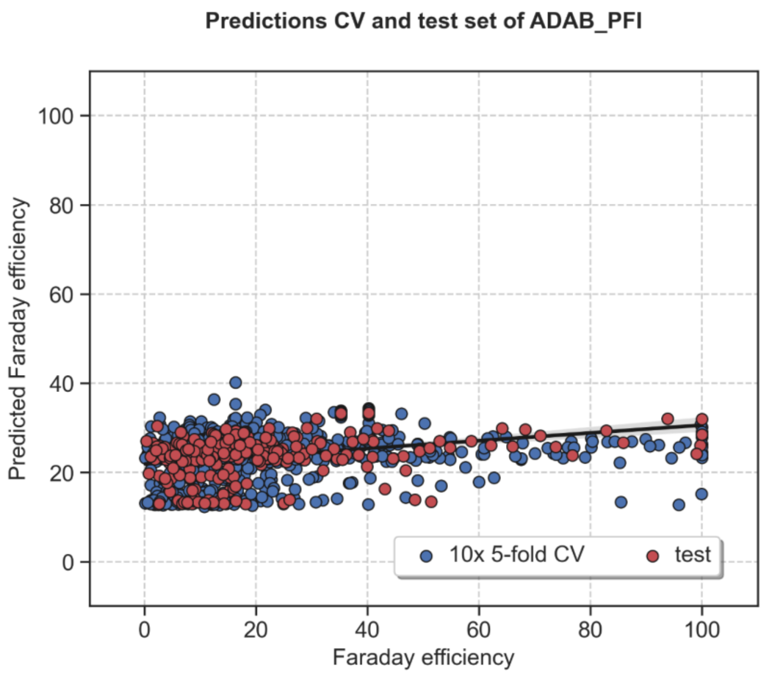

Figure S25. Prediction performance of the AdaBoost model using features selected by permutation feature importance using ROBERT program. The scatter plots show predicted versus actual eNRR Faradaic efficiency values for both the 10-times-repeated 5-fold cross-validation set (10×5-fold CV, blue dots) and the test set (red dots). The performance metrics were: 10×5-fold CV (R^2 = 0.07, MAE = 13.0, RMSE = 18.0) and test set (R^2 = 0.10, MAE = 14.0, RMSE = 19.0).

As shown in the figure above, the best-performing regression model identified by ROBERT—an AdaBoost regressor using features selected by permutation importance—failed to establish a meaningful predictive relationship. The model's performance on the hold-out test set was poor, achieving an R^2 of just 0.10, with an MAE of 14.0 and an RMSE of 19.0. The scatter plot visually corroborates these metrics, showing a cloud of points with virtually no correlation to the ideal diagonal line. The predictions are confined to a narrow band, indicating the model is unable to differentiate between low- and high-performing catalysts. This empirical result provides strong evidence that a direct regression approach for FE is not viable for this dataset.

Here is the command for running the ROBERT on the FE datasets: *python -m robert --csv_name "FE_processed_for_ml.csv" --y "Faradaic efficiency" --names "Name" --ignore "['Name']" --auto_type "False" --seed "49" --kfold "5" --repeat_kfolds "10" --corr_filter_x "False" –model "['AdaB','GB','GP','MVL','NN','RF']"*.

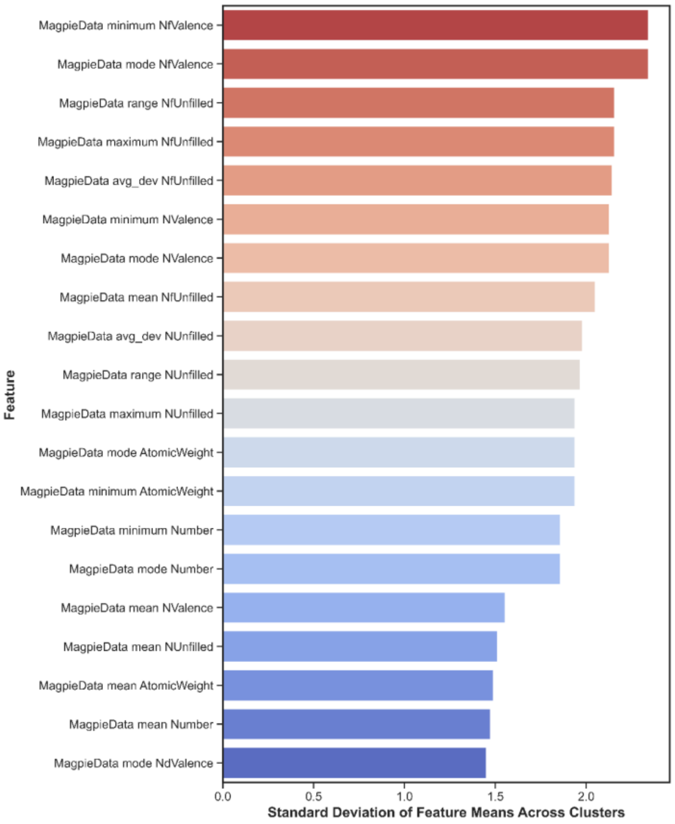

Figure S26. The standard deviation of feature means across clusters of Faradaic efficiency for the top 20 features.

Figure S26 reveals that descriptors derived from elemental properties, particularly the "valence electron number", are highly influential in determining Faradaic efficiency (FE). The physical significance of this statistical correlation lies in the fact that the valence electron count serves as a powerful proxy for the catalyst's fundamental electronic structure, which governs the critical competition between the eNRR and the hydrogen evolution reaction (HER). The high FE of catalysts in Cluster 3 can be rationalized through the following interconnected electronic principles.

Modulation of the d-band center (ε_d) for N_2 Activation: The number of valence electrons directly relates to the filling of the transition metal's d-band. According to the d-band center theory, this occupancy determines the energy of the ε_d relative to the Fermi level[6]. An optimally positioned ε_d is crucial for facilitating the "acceptance-donation" mechanism, enabling effective π-backdonation from the catalyst's d-orbitals into N_2's antibonding orbitals. This step is essential for activating the inert N≡N bond[7].

Simultaneous Suppression of HER: Critically for selectivity, the same electronic tuning that promotes eNRR also serves to inhibit HER. A d-band center that is shifted moderately lower in energy weakens the adsorption of hydrogen intermediates (*H). This increases the free energy of the initial protonation step, thereby kinetically suppressing the HER pathway and favoring eNRR.

Influence of Work Function and Dynamic Effects: The electronic structure also influences the work function (Φ). As reported in the literature, a lower work function can promote electron transfer from the catalyst surface to adsorbed N_2, further facilitating N_2 dissociation[8, 9]. Furthermore, a key dynamic effect arises from competitive adsorption: the strong adsorption of N_2

can effectively passivate the active sites, making them electronically or sterically less available for H^+ adsorption[10]. This self-regulating mechanism further enhances selectivity towards ammonia synthesis.

In summary, the high FE of catalysts in Cluster 3 is mechanistically rooted in an electronic structure that is finely tuned to simultaneously promote N_2 activation and inhibit hydrogen evolution. This provides a clear, electronically-driven guideline for designing next-generation, highly selective eNRR electrocatalysts, validating the data-driven insights from our clustering approach.

5. Case Study: Direct Querying of GPT-4o-mini on Catalyst Performance

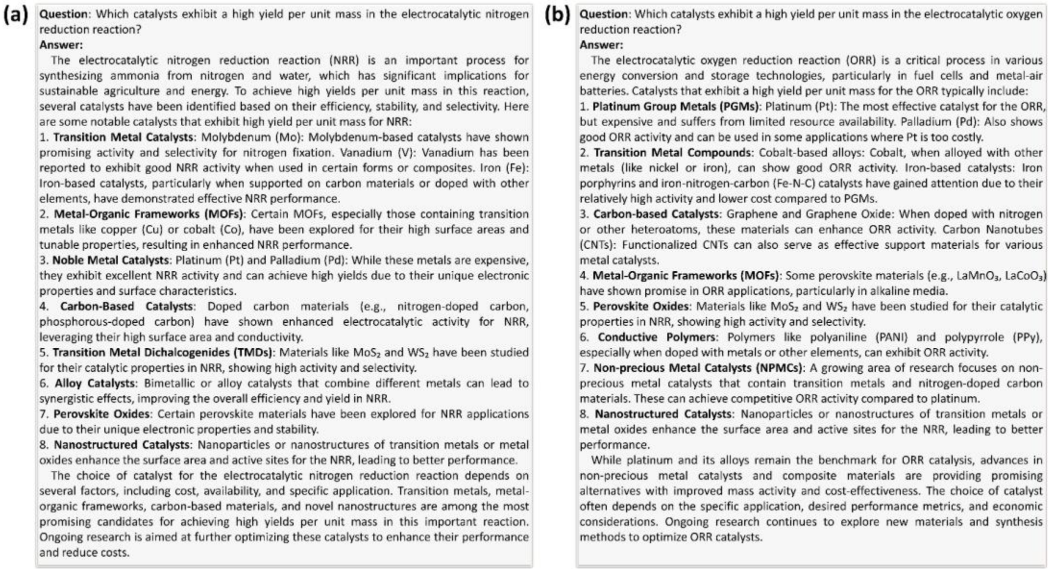

(a)

Question: Which catalysts exhibit a high yield per unit mass in the electrocatalytic nitrogen reduction reaction?

Answer:

The electrocatalytic nitrogen reduction reaction (NRR) is an important process for synthesizing ammonia from nitrogen and water, which has significant implications for sustainable agriculture and energy. To achieve high yields per unit mass in this reaction, several catalysts have been identified based on their efficiency, stability, and selectivity. Here are some notable catalysts that exhibit high yield per unit mass for NRR:

1. **Transition Metal Catalysts**: Molybdenum (Mo): Molybdenum-based catalysts have shown promising activity and selectivity for nitrogen fixation. Vanadium (V): Vanadium has been reported to exhibit good NRR activity when used in certain forms or composites. Iron (Fe): Iron-based catalysts, particularly when supported on carbon materials or doped with other elements, have demonstrated effective NRR performance.
2. **Metal-Organic Frameworks (MOFs)**: Certain MOFs, especially those containing transition metals like copper (Cu) or cobalt (Co), have been explored for their high surface areas and tunable properties, resulting in enhanced NRR performance.
3. **Noble Metal Catalysts**: Platinum (Pt) and Palladium (Pd): While these metals are expensive, they exhibit excellent NRR activity and can achieve high yields due to their unique electronic properties and surface characteristics.
4. **Carbon-Based Catalysts**: Doped carbon materials (e.g., nitrogen-doped carbon, phosphorous-doped carbon) have shown enhanced electrocatalytic activity for NRR, leveraging their high surface area and conductivity.
5. **Transition Metal Dichalcogenides (TMDs)**: Materials like MoS_2 and WS_2 have been studied for their catalytic properties in NRR, showing high activity and selectivity.
6. **Alloy Catalysts**: Bimetallic or alloy catalysts that combine different metals can lead to synergistic effects, improving the overall efficiency and yield in NRR.
7. **Perovskite Oxides**: Certain perovskite materials have been explored for NRR applications due to their unique electronic properties and stability.
8. **Nanostructured Catalysts**: Nanoparticles or nanostructures of transition metals or metal oxides enhance the surface area and active sites for the NRR, leading to better performance.

The choice of catalyst for the electrocatalytic nitrogen reduction reaction depends on several factors, including cost, availability, and specific application. Transition metals, metal-organic frameworks, carbon-based materials, and novel nanostructures are among the most promising candidates for achieving high yields per unit mass in this important reaction. Ongoing research is aimed at further optimizing these catalysts to enhance their performance and reduce costs.

(b)

Question: Which catalysts exhibit a high yield per unit mass in the electrocatalytic oxygen reduction reaction?

Answer:

The electrocatalytic oxygen reduction reaction (ORR) is a critical process in various energy conversion and storage technologies, particularly in fuel cells and metal-air batteries. Catalysts that exhibit a high yield per unit mass for the ORR typically include:

1. **Platinum Group Metals (PGMs)**: Platinum (Pt): The most effective catalyst for the ORR, but expensive and suffers from limited resource availability. Palladium (Pd): Also shows good ORR activity and can be used in some applications where Pt is too costly.
2. **Transition Metal Compounds**: Cobalt-based alloys: Cobalt, when alloyed with other metals (like nickel or iron), can show good ORR activity. Iron-based catalysts: Iron porphyrins and iron-nitrogen-carbon (Fe-N-C) catalysts have gained attention due to their relatively high activity and lower cost compared to PGMs.
3. **Carbon-based Catalysts**: Graphene and Graphene Oxide: When doped with nitrogen or other heteroatoms, these materials can enhance ORR activity. Carbon Nanotubes (CNTs): Functionalized CNTs can also serve as effective support materials for various metal catalysts.
4. **Metal-Organic Frameworks (MOFs)**: Some perovskite materials (e.g., $LaMnO_3$, $LaCoO_3$) have shown promise in ORR applications, particularly in alkaline media.
5. **Perovskite Oxides**: Materials like MoS_2 and WS_2 have been studied for their catalytic properties in NRR, showing high activity and selectivity.
6. **Conductive Polymers**: Polymers like polyaniline (PANI) and polypyrrole (PPy), especially when doped with metals or other elements, can exhibit ORR activity.
7. **Non-precious Metal Catalysts (NPMCs)**: A growing area of research focuses on non-precious metal catalysts that contain transition metals and nitrogen-doped carbon materials. These can achieve competitive ORR activity compared to platinum.
8. **Nanostructured Catalysts**: Nanoparticles or nanostructures of transition metals or metal oxides enhance the surface area and active sites for the NRR, leading to better performance.

While platinum and its alloys remain the benchmark for ORR catalysis, advances in non-precious metal catalysts and composite materials are providing promising alternatives with improved mass activity and cost-effectiveness. The choice of catalyst often depends on the specific application, desired performance metrics, and economic considerations. Ongoing research continues to explore new materials and synthesis methods to optimize ORR catalysts.

Figure S27. (a) The responses to “Which catalysts exhibit a high yield per unit mass in the electrocatalytic nitrogen reduction reaction?” posed directly to GPT-4o-mini. The answer was created on 2024/12/11. (b) The responses to “Which catalysts exhibit a high yield per unit mass in the electrocatalytic oxygen reduction reaction?” posed directly to GPT-4o-mini. The answer was created on 2024/12/11.

As demonstrated above, the response provided by GPT-4o-mini without GraphRAG merely lists common catalyst types without offering meaningful guidance. The answer lacks specific eNRR yield values and contains several factual inaccuracies. For instance, it incorrectly includes Pd and Pt as prominent NRR catalysts when these noble metals are actually more commonly used in oxygen reduction reactions.

6. Application and Validation of the eNRRCrew

```python
import pandas as pd
import matplotlib.pyplot as plt

# Load the CSV file
data =
pd.read_csv('data_include_morphology_electrocatalyst.csv')

# Extract the relevant columns
x = data['Applied Potential (Faraday Efficiency)']
y = data['Faraday efficiency']

# Create a scatter plot
plt.figure(figsize=(10, 6))
plt.scatter(x, y, alpha=0.5)
plt.title('Relationship between Applied Potential and
Faraday Efficiency')
plt.xlabel('Applied Potential (Faraday Efficiency)')
plt.ylabel('Faraday Efficiency')
plt.grid()
plt.show()
```

Figure S28. The Python code generated by CSV_Handler related to Figure 5c in the manuscript.

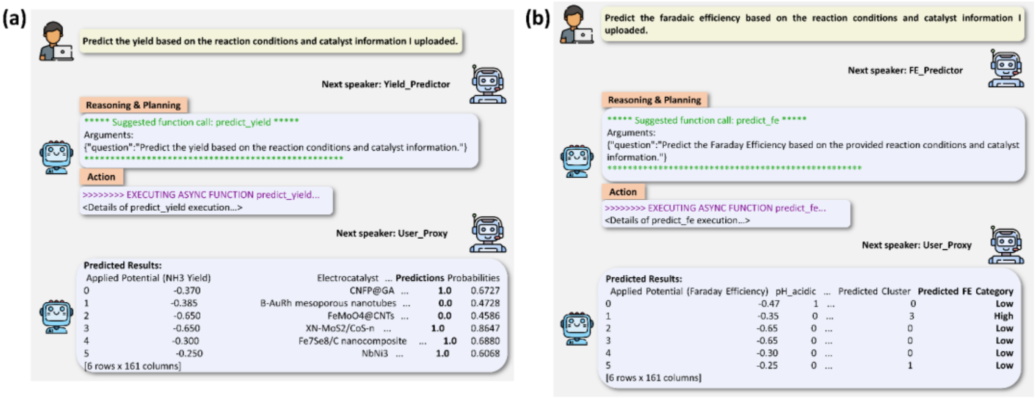

Figure S29. Examples demonstrating eNRRCrew's ability to predict eNRR yield and Faradaic efficiency based on user-provided information. (a) Dialogue illustrating the use of eNRRCrew to predict yield and (b) Faradaic efficiency based on uploaded reaction conditions and electrocatalyst information.

ENRRCrew demonstrates versatile prediction capability for both eNRR yield and FE based on user-provided information. To simulate real-world usage scenarios, we conducted a search on Google Scholar with the keyword "nitrogen reduction reaction" and sorted the results by date. This search yielded the six most recent publications related to eNRR as listed in Table S5. We extracted relevant electrocatalyst information and reaction condition data from these studies based on the workflow shown in **Figure 1** in the main text and input them into eNRRCrew to predict the yield and FE. As shown in Figure S29, the crew processes input data on the electrocatalyst composition and properties, along with reaction parameters. Leveraging its integrated machine learning predictors, eNRRCrew generates predictions for both eNRR yield and FE. Figure S29a illustrates the yield prediction functionality, where the system provides predicted yields along with associated probability for various electrocatalysts. The process begins with a question to predict the eNRR yield based on the provided reaction conditions and electrocatalyst information. This task is assigned to the Yield Predictor, which suggests using the predict_yield function. The system then executes this function asynchronously, automating the processes of data preprocessing, feature engineering, and predictive model execution. In this case, the prediction accuracy achieves 0.67, with the corresponding distribution of predicted yield likelihood and experimental yields

illustrated in Figure S30. Figure S29b showcases the FE prediction capability similar to the yield prediction process, it can be observed from Figure S31, that the median FE of data points predicted as high is 22.5, which is significantly higher than the median FE of data points predicted as low (16.14). These functionalities not only streamline yield and FE prediction but also empower researchers to evaluate and optimize catalyst designs more effectively.

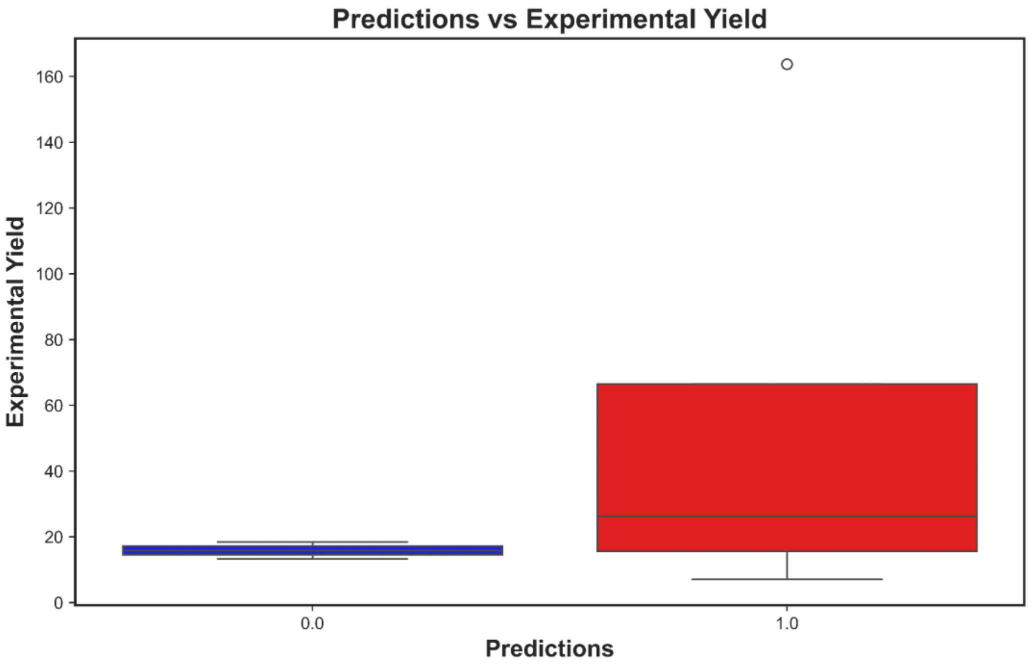

Figure S30. The predictions versus experimental eNRR yield correspond to Figure S29a in the manuscript. 0 for low yield and 1 for high yield.

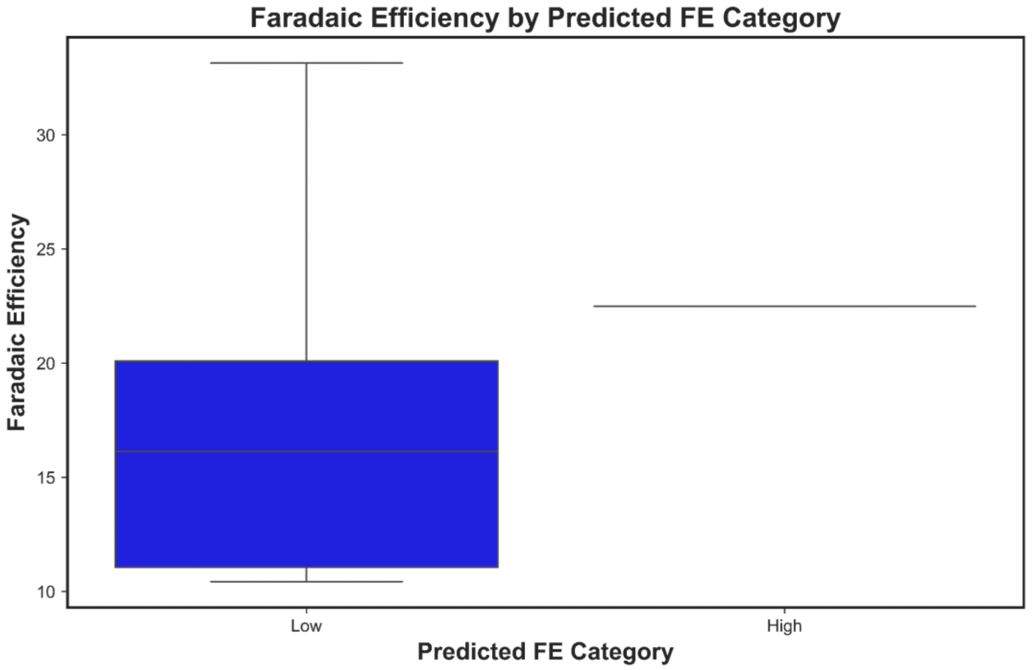

Figure S31. The predictions versus experimental Faradaic efficiency correspond to Figure S29b in the manuscript. Low for low Faradaic efficiency and high for high Faradaic efficiency.

7. Impact of Dataset Size on Model Performance

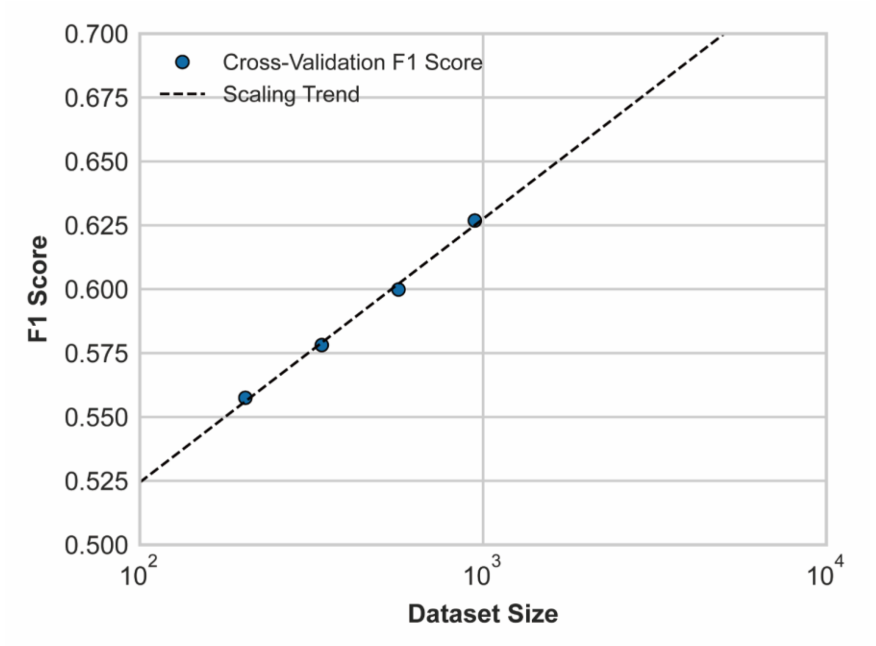

Figure S32. Scaling analysis of model performance. Cross-validation F1 score for the eNRR yield prediction model as a function of the training dataset size. The dashed line represents the scaling trend, indicating that model performance consistently improves with the availability of more data. This demonstrates both the utility of the current dataset and the potential for further enhancement as more high-quality data becomes available in the field.

To address the question of data sufficiency and to understand the potential for future improvements, we investigated how the performance of our machine learning model scales with the size of the training dataset. We trained our random forest classifier for eNRR yield prediction on progressively larger, randomly sampled subsets of our full dataset (specifically, at sizes of 203, 339, 567, and 946 data points). For each subset size, we performed 10-fold cross-validation and recorded the average F1 score. The results are plotted in Figure S32. A clear and positive correlation is observed between the dataset size and the model's F1 score. The performance steadily increases from 0.56 with 203 data points to over 0.62 with 946 data points. This analysis leads to two important conclusions: The current dataset, despite being derived from a specialized field, is sufficiently large and diverse to train a model with meaningful predictive power, as demonstrated by the reasonable F1 scores achieved. There is significant potential for further performance gains. As the field of electrochemical nitrogen reduction continues to grow and more high-quality experimental data is published, our automated framework can facilitate dataset expansion, which, according to this trend, will lead to more accurate and reliable predictive models. This underscores the importance of community-wide efforts in data sharing and standardization to accelerate data-driven catalyst design.

8. Supporting tables

Table S1. Top 25 words for each cluster in eNRR corpus.

Cluster Label	Top 25 Words (with frequency counts of each word)
0	('efficiency', 313), ('high', 312), ('mu', 282), ('yield', 268), ('g', 214), ('V', 211), ('active', 208), ('h(-1)', 208), ('rate', 200), ('activity', 196), ('efficient', 190), ('surface', 186), ('hydrogen', 164), ('performance', 160), ('oxygen', 154), ('sites', 153), ('carbon', 140), ('electrode', 129), ('fixation', 127), ('adsorption', 122), ('Faradaic', 119), ('metal', 118), ('energy', 116), ('work', 115), ('nitrate', 113)
1	('high', 323), ('synthesis', 312), ('efficiency', 300), ('production', 287), ('energy', 271), ('ambient', 254), ('yield', 252), ('Haber-Bosch', 240), ('hydrogen', 231), ('V', 209), ('process', 205), ('mu', 183), ('h(-1)', 177), ('rate', 172), ('efficient', 166), ('g', 160), ('also', 148), ('Faradaic', 143), ('active', 139), ('alternative', 136), ('promising', 129), ('activity', 129), ('conditions.', 124), ('using', 121), ('sustainable', 120)
2	('energy', 117), ('metal', 116), ('hydrogen', 98), ('high', 92), ('evolution', 83), ('activity', 72), ('active', 71), ('carbon', 69), ('structure', 68), ('oxygen', 66), ('performance', 64), ('potential', 62), ('also', 60), ('new', 59), ('materials', 59), ('photocatalytic', 56), ('electron', 55), ('electronic', 54), ('surface', 54), ('sites', 49), ('atoms', 47), ('density', 46), ('strategy', 45), ('work', 44), ('V', 44)
3	('high', 239), ('activity', 220), ('efficiency', 191), ('Mo', 188), ('hydrogen', 181), ('efficient', 176), ('yield', 175), ('V', 171), ('active', 167), ('MoS2', 160), ('mu', 148), ('performance', 143), ('energy', 141), ('ambient', 138), ('g', 124), ('potential', 122), ('sites', 121), ('selectivity', 115), ('rate', 115), ('functional', 113), ('metal', 112), ('adsorption', 110), ('h(-1)', 106), ('low', 106), ('theory', 104)
4	('energy', 97), ('fixation', 78), ('synthesis', 73), ('potential', 60), ('production', 54), ('electrolyte', 50), ('process', 47), ('method', 46), ('water', 44), ('also', 44), ('electron', 44), ('using', 43), ('high', 43), ('current', 42), ('system', 41), ('activity', 40), ('electrode', 40), ('However,', 39), ('chemical', 39), ('used', 39), ('proton', 39), ('efficiency', 36), ('study', 35), ('nitrogenase', 35), ('reactions', 34)
5	('activity', 424), ('potential', 332), ('high', 329), ('metal', 298), ('efficient', 255), ('energy', 253), ('atoms', 245), ('density', 243), ('adsorption', 235), ('functional', 234), ('atom', 233), ('limiting', 228), ('promising', 215), ('theory', 210), ('selectivity', 204), ('hydrogen', 199), ('low', 196), ('transition', 194), ('active', 194), ('single', 190), ('performance', 188), ('work', 176), ('V', 173), ('design', 164), ('=', 162)
6	('high', 491), ('efficiency', 438), ('yield', 422), ('mu', 355), ('hydrogen', 341), ('activity', 335), ('g', 314), ('V', 313), ('ambient', 298), ('h(-1)', 297), ('active', 293), ('rate', 285), ('efficient', 271), ('Faradaic', 228), ('promising', 226), ('energy', 222), ('performance', 215), ('selectivity', 211), ('low', 210), ('However,', 199), ('process', 190), ('synthesis', 190), ('production', 188), ('Haber-Bosch', 188), ('sites', 183)

Cluster 0 emphasizes the efficiency and performance metrics of eNRR. The frequent mention of terms like “efficiency”, “yield”, and “mu” indicates a focus on optimizing the electrocatalytic efficiency and performance of eNRR systems. This cluster mainly includes research on improving the efficiency and performance of eNRR.

Cluster 1 appears to focus on the synthesis processes and production efficiency of NRR. The

frequent mention of "synthesis," "production," and "Haber-Bosch" suggests a comparison with traditional ammonia synthesis methods. The interest in "ambient" conditions indicates research aimed at more sustainable and energy-efficient production methods. This cluster is likely concerned with developing new synthesis routes and optimizing existing processes for better efficiency and lower energy consumption.

Cluster 2 focuses on the material properties and their catalytic activities. Keywords like "metal," "hydrogen," "evolution," and "carbon" suggest a detailed examination of different materials (e.g., metals, and carbon-based materials) and their capabilities in eNRR. The mention of "structure," "surface," and "density" indicates a focus on the structural properties of catalysts and their impact on catalytic performance. This cluster is likely involved in material characterization and the development of new materials for enhanced eNRR activity.

Cluster 3 is centered around molybdenum-based catalysts, as indicated by the frequent mention of "Mo" and "MoS_2." The focus is on the activity and efficiency of these catalysts in eNRR. The presence of terms like "yield," "active," and "performance" suggests an emphasis on optimizing the catalytic effectiveness of molybdenum-based materials. This cluster likely investigates the properties and performance of Mo-based catalysts and explores ways to enhance their efficiency in eNRR.

Cluster 4 highlights experimental methods and processes used in eNRR. The frequent occurrence of "fixation," "synthesis," and "process" indicates research on different experimental techniques and methodologies. The mention of "electrolyte," "water," and "electron" suggests studies on the role of electrolytes and electron transfer processes in eNRR. This cluster likely focuses on the development and optimization of experimental setups and processes for efficient eNRR.

Cluster 5 is likely focused on theoretical and computational studies of eNRR. The frequent mention of "potential," "atoms," and "adsorption" suggests a focus on understanding the fundamental mechanisms and interactions at the atomic level. The presence of terms like "density," "theory" and "functional" indicates the use of theoretical models and computational simulations to study electrocatalytic processes. This cluster is probably involved in predicting and explaining the behavior of electrocatalysts using computational methods.

Cluster 6 encompasses a broad range of studies focused on general performance metrics and promising approaches in eNRR. The high frequencies of "high," "efficiency," "yield," and "activity" indicate a strong emphasis on overall performance improvements. The mention of "ambient" and "Faradaic" suggests research on conditions and efficiencies under different operational settings. This cluster likely includes comprehensive studies aiming to enhance the overall effectiveness and practicality of eNRR processes.

Each cluster represents a distinct aspect of research in the field of electrocatalytic NRR, ranging from efficiency optimization, synthesis processes, material properties, molybdenum-based catalysts, experimental methods, and theoretical studies, to general performance enhancements. Understanding these clusters can help guide future research directions and identify key areas for further investigation and development in eNRR technology.

Table S2. The descriptors in the dataset for machine learning with brief descriptions.

Variables	Descriptions
Applied Potential (NH3 Yield)	The applied electrode potential when measuring the eNRR yield
Applied Potential (Faradaic efficiency)	The applied electrode potential when measuring the faradaic efficiency
pH_acidic	Acidic environment
pH_alkaline	Alkaline environment
pH_neutral	Neutral environment
pH_weak acid	Weak acidic environment
Electrolyte without concentration_h2so4	Use of sulfuric acid as an electrolyte without specifying concentration
Electrolyte without concentration_hcl	Use of hydrochloric acid as an electrolyte without specifying concentration
Electrolyte without concentration_k2so4	Use of potassium sulfate as an electrolyte without specifying concentration
Electrolyte without concentration_koh	Use of potassium hydroxide as an electrolyte without specifying concentration
Electrolyte without concentration_li2so4	Use of lithium sulfate as an electrolyte without specifying concentration
Electrolyte without concentration_liclo4	Use of lithium perchlorate as an electrolyte without specifying concentration
Electrolyte without concentration_na2so4	Use of sodium sulfate as an electrolyte without specifying concentration
Electrolyte without concentration_pbs	Use of phosphate-buffered saline as an electrolyte without specifying concentration
N-15 labeling_mentioned	Indication of whether nitrogen-15 isotope labeling is mentioned
MagpieData minimum Number	The minimum value of the atomic number from Magpie
MagpieData maximum Number	The maximum value of the atomic number from Magpie
MagpieData range Number	The range of the atomic number from Magpie
MagpieData mean Number	The average value of the atomic number from Magpie
MagpieData avg_dev Number	The average deviation of the atomic number from Magpie
MagpieData mode Number	The most common value of the atomic number from Magpie
MagpieData minimum MendeleevNumber	The minimum value of Mendeleev Number from Magpie
MagpieData maximum MendeleevNumber	The maximum value of Mendeleev Number from Magpie
MagpieData range MendeleevNumber	The range of Mendeleev Number from Magpie
MagpieData mean MendeleevNumber	The average value of Mendeleev Number from Magpie
MagpieData avg_dev MendeleevNumber	The average deviation of Mendeleev Number from Magpie
MagpieData mode MendeleevNumber	The most common value of Mendeleev Number from Magpie
MagpieData minimum AtomicWeight	The minimum value of Atomic Weight from Magpie
MagpieData maximum AtomicWeight	The maximum value of Atomic Weight from Magpie
MagpieData range AtomicWeight	The range of Atomic Weight from Magpie
MagpieData mean	The average value of Atomic Weight from Magpie

AtomicWeight	
MagpieData avg_dev AtomicWeight	The average deviation of Atomic Weight from Magpie
MagpieData mode AtomicWeight	The most common value of Atomic Weight from Magpie
MagpieData minimum MeltingT	The minimum value of Melting Temperature from Magpie
MagpieData maximum MeltingT	The maximum value of Melting Temperature from Magpie
MagpieData range MeltingT	The range of Melting Temperature from Magpie
MagpieData mean MeltingT	The average value of Melting Temperature from Magpie
MagpieData avg_dev MeltingT	The average deviation of Melting Temperature from Magpie
MagpieData mode MeltingT	The most common value of Melting Temperature from Magpie
MagpieData minimum Column	The minimum value of Column in the periodic table from Magpie
MagpieData maximum Column	The maximum value of Column in the periodic table from Magpie
MagpieData range Column	The range of Column in the periodic table from Magpie
MagpieData mean Column	The average value of Column in the periodic table from Magpie
MagpieData avg_dev Column	The average deviation of Column in the periodic table from Magpie
MagpieData mode Column	The most common value of Column in the periodic table from Magpie
MagpieData minimum Row	The minimum value of Row in the periodic table from Magpie
MagpieData maximum Row	The maximum value of Row in the periodic table from Magpie
MagpieData range Row	The range of Row in the periodic table from Magpie
MagpieData mean Row	The average value of Row in the periodic table from Magpie
MagpieData avg_dev Row	The average deviation of Row in the periodic table from Magpie
MagpieData mode Row	The most common value of Row in the periodic table from Magpie
MagpieData minimum CovalentRadius	The minimum value of Covalent Radius from Magpie
MagpieData maximum CovalentRadius	The maximum value of Covalent Radius from Magpie
MagpieData range CovalentRadius	The range of Covalent Radius from Magpie
MagpieData mean CovalentRadius	The average value of Covalent Radius from Magpie
MagpieData avg_dev CovalentRadius	The average deviation of Covalent Radius from Magpie
MagpieData mode CovalentRadius	The most common value of Covalent Radius from Magpie
MagpieData minimum Electronegativity	The minimum value of Electronegativity from Magpie
MagpieData maximum Electronegativity	The maximum value of Electronegativity from Magpie
MagpieData range Electronegativity	The range of Electronegativity from Magpie
MagpieData mean Electronegativity	The average value of Electronegativity from Magpie

MagpieData avg_dev Electronegativity	The average deviation of Electronegativity from Magpie
MagpieData mode Electronegativity	The most common value of Electronegativity from Magpie
MagpieData minimum NsValence	The minimum value the number of s Valence Electrons from Magpie
MagpieData maximum NsValence	The maximum value of the number of s Valence Electrons from Magpie
MagpieData range NsValence	The range of the number of s Valence Electrons from Magpie
MagpieData mean NsValence	The average value of the number of s Valence Electrons from Magpie
MagpieData avg_dev NsValence	The average deviation of the number of s Valence Electrons from Magpie
MagpieData mode NsValence	The most common value of the number of s Valence Electrons from Magpie
MagpieData minimum NpValence	The minimum value of the number of p Valence Electrons from Magpie
MagpieData maximum NpValence	The maximum value of the number of p Valence Electrons from Magpie
MagpieData range NpValence	The range of the number of p Valence Electrons from Magpie
MagpieData mean NpValence	The average value of the number of p Valence Electrons from Magpie
MagpieData avg_dev NpValence	The average deviation of the number of p Valence Electrons from Magpie
MagpieData mode NpValence	The most common value of the number of p Valence Electrons from Magpie
MagpieData minimum NdValence	The minimum value of the number of d Valence Electrons from Magpie
MagpieData maximum NdValence	The maximum value of the number of d Valence Electrons from Magpie
MagpieData range NdValence	The range of the number of d Valence Electrons from Magpie
MagpieData mean NdValence	The average value of the number of d Valence Electrons from Magpie
MagpieData avg_dev NdValence	The average deviation of the number of d Valence Electrons from Magpie
MagpieData mode NdValence	The most common value of the number of d Valence Electrons from Magpie
MagpieData minimum NfValence	The minimum value of the number of f Valence Electrons from Magpie
MagpieData maximum NfValence	The maximum value of the number of f Valence Electrons from Magpie
MagpieData range NfValence	The range of the number of f Valence Electrons from Magpie
MagpieData mean NfValence	The average value of the number of f Valence Electrons from Magpie
MagpieData avg_dev NfValence	The average deviation of the number of f Valence Electrons from Magpie
MagpieData mode NfValence	The most common value of the number of f Valence Electrons from Magpie
MagpieData minimum NValence	The minimum value of the total number of Valence Electrons from Magpie
MagpieData maximum NValence	The maximum value of the total number of Valence Electrons from Magpie
MagpieData range NValence	The range of the total number of Valence Electrons from Magpie
MagpieData mean NValence	The average value of the total number of Valence Electrons from Magpie

MagpieData avg_dev NValence	The average deviation of the total number of Valence Electrons from Magpie
MagpieData mode NValence	The most common value of the total number of Valence Electrons from Magpie
MagpieData minimum NsUnfilled	The minimum value of the number of unfilled s states from Magpie
MagpieData maximum NsUnfilled	The maximum value of the number of unfilled s states from Magpie
MagpieData range NsUnfilled	The range of the number of unfilled s states from Magpie
MagpieData mean NsUnfilled	The average value of the number of unfilled s states from Magpie
MagpieData avg_dev NsUnfilled	The average deviation of the number of unfilled s states from Magpie
MagpieData mode NsUnfilled	The most common value of the number of unfilled s states from Magpie
MagpieData minimum NpUnfilled	The minimum value of the number of unfilled p states from Magpie
MagpieData maximum NpUnfilled	The maximum value of the number of unfilled p states from Magpie
MagpieData range NpUnfilled	The range of the number of unfilled p states from Magpie
MagpieData mean NpUnfilled	The average value of the number of unfilled p states from Magpie
MagpieData avg_dev NpUnfilled	The average deviation of the number of unfilled p states from Magpie
MagpieData mode NpUnfilled	The most common value of the number of unfilled p states from Magpie
MagpieData minimum NdUnfilled	The minimum value of the number of unfilled d states from Magpie
MagpieData maximum NdUnfilled	The maximum value of the number of unfilled d states from Magpie
MagpieData range NdUnfilled	The range of the number of unfilled d states from Magpie
MagpieData mean NdUnfilled	The average value of the number of unfilled d states from Magpie
MagpieData avg_dev NdUnfilled	The average deviation of the number of unfilled d states from Magpie
MagpieData mode NdUnfilled	The most common value of the number of unfilled d states from Magpie
MagpieData minimum NfUnfilled	The minimum value of the number of unfilled f states from Magpie
MagpieData maximum NfUnfilled	The maximum value of the number of unfilled f states from Magpie
MagpieData range NfUnfilled	The range of the number of unfilled f states from Magpie
MagpieData mean NfUnfilled	The average value of the number of unfilled f states from Magpie
MagpieData avg_dev NfUnfilled	The average deviation of the number of unfilled f states from Magpie
MagpieData mode NfUnfilled	The most common value of the number of unfilled f states from Magpie
MagpieData minimum NUnfilled	The minimum value of the total number of unfilled states from Magpie
MagpieData maximum NUnfilled	The maximum value of the total number of unfilled states from Magpie
MagpieData range NUnfilled	The range of the total number of unfilled states from Magpie
MagpieData mean NUnfilled	The average value of the total number of unfilled states from

	Magpie
MagpieData avg_dev NUnfilled	The average deviation of the total number of unfilled states from Magpie
MagpieData mode NUnfilled	The most common value of the total number of unfilled states from Magpie
MagpieData minimum GSvolume_pa	The minimum value of the specific volume of 0 K ground state from Magpie
MagpieData maximum GSvolume_pa	The maximum value of the specific volume of 0 K ground state from Magpie
MagpieData range GSvolume_pa	The range of the specific volume of 0 K ground state from Magpie
MagpieData mean GSvolume_pa	The average value of the specific volume of 0 K ground state from Magpie
MagpieData avg_dev GSvolume_pa	The average deviation of the specific volume of 0 K ground state from Magpie
MagpieData mode GSvolume_pa	The most common value of the specific volume of 0 K ground state from Magpie
MagpieData minimum GSbandgap	The minimum value of the band gap energy of 0 K ground state from Magpie
MagpieData maximum GSbandgap	The maximum value of the band gap energy of 0 K ground state from Magpie
MagpieData range GSbandgap	The range of the band gap energy of 0 K ground state from Magpie
MagpieData mean GSbandgap	The average value of the band gap energy of 0 K ground state from Magpie
MagpieData avg_dev GSbandgap	The average deviation of the band gap energy of 0 K ground state from Magpie
MagpieData mode GSbandgap	The most common value of the band gap energy of 0 K ground state from Magpie
MagpieData minimum GSmagmom	The minimum value of the magnetic moment (per atom) of 0 K ground state from Magpie
MagpieData maximum GSmagmom	The maximum value of the magnetic moment (per atom) of 0 K ground state from Magpie
MagpieData range GSmagmom	The range of the magnetic moment (per atom) of 0 K ground state from Magpie
MagpieData mean GSmagmom	The average value of the magnetic moment (per atom) of 0 K ground state from Magpie
MagpieData avg_dev GSmagmom	The average deviation of the magnetic moment (per atom) of 0 K ground state from Magpie
MagpieData mode GSmagmom	The most common value of the magnetic moment (per atom) of 0 K ground state from Magpie
MagpieData minimum SpaceGroupNumber	The minimum value of the space group number of 0 K ground state from Magpie
MagpieData maximum SpaceGroupNumber	The maximum value of the space group number of 0 K ground state from Magpie
MagpieData range SpaceGroupNumber	The range of the space group number of 0 K ground state from Magpie
MagpieData mean SpaceGroupNumber	The average value of the space group number of 0 K ground state from Magpie
MagpieData avg_dev SpaceGroupNumber	The average deviation of the space group number of 0 K ground state from Magpie
MagpieData mode SpaceGroupNumber	The most common value of the space group number of 0 K ground state from Magpie
Structure Type_Hollow Structures	Structures characterized by hollow interiors
Structure Type_Hybrids and	Structures made from a combination of different materials

Composites	
Structure Type_Nanocubes	Cube-shaped nanostructures
Structure Type_Nanofibers	Fiber-like nanostructures
Structure Type_Nanoparticles	Small particles at the nanoscale
Structure Type_Nanorods	Rod-shaped nanostructures
Structure Type_Nanosheets	Sheet-like nanostructures
Structure Type_Nanotubes	Tube-shaped nanostructures
Structure Type_Nanowalls	Wall-like nanostructures
Structure Type_Nanowires	Wire-like nanostructures
Structure Type_Other	Other types of structures not specifically categorized
Structure Type_Porous Structures	Structures characterized by a porous nature

Note: Applied potentials, NH_3 yields, and Faradaic efficiencies were extracted from the source abstracts. Electrolyte pH and type were classified with a post-processing script as shown in Section 11. Elemental descriptors were generated with Magpie, and structural descriptors were automatically parsed from the abstracts with ChatGPT, as shown in Section 12.

Table S3. The performance metrics on test set of various machine learning models for predicting eNRR yield after optimal hyperparameters finding by Optuna.

Machine learning models	Precision	Recall	F1-score	Accuracy
Random forest classifier	0.63	0.63	0.63	0.63
Support vector classifier	0.59	0.59	0.59	0.59
XGBoost classifier	0.60	0.60	0.60	0.60
Artificial neural networks	0.60	0.60	0.60	0.60

Table S4. The optimal hyperparameters for the random forest classifier predicting eNRR yield were identified using Optuna.

Parameter	Value
n_estimators	116
max_depth	14
min_samples_split	3
min_samples_leaf	14
bootstrap	False

Table S5. The collected recent articles about eNRR from Google Scholar using the query “nitrogen reduction reaction”.

Index	Article DOI
1	https://papers.ssrn.com/sol3/papers.cfm?abstract_id=5047126
2	https://iopscience.iop.org/article/10.1088/1361-6528/ad9aad/meta
3	https://www.sciencedirect.com/science/article/pii/S0360319924049012
4	https://www.sciencedirect.com/science/article/pii/S0360319924050213
5	https://pubs.rsc.org/en/content/articlelanding/2024/cc/d4cc04683h/unauth
6	https://onlinelibrary.wiley.com/doi/abs/10.1002/ange.202418035

9. The prompt used in automated structure-activity relationships extraction workflow

```
for context in context_list:
    print("Start to analyze paper " + str(column1_value) )
    user_heading = """Q: Can you summarize the following details from an abstract of a electrocatalytic nitrogen reduction readtion
    in a table. The table, includes information about the method for N2 reduction (Electrochemical, Phoelectrochemical or N/A),
    the reactant (specified using molecular or ion formulas such as N2, NO2-, or NO3-), the product (specified using molecular or ion
    formulas like NH3, NO3-, or NO2-), the Faradaic efficiency, NH3 yield, applied potential, electrolyte (e.g., 0.1M H2SO4, 0.1M HCl,
    or Na2SO4), the electrocatalyst (like Ru, Pt, Fe2O3/C, etc. And Please include the full name if possible, like 2D HsGDY (Hydrogen
    -substituted graphdiyne)), the elements of electrocatalyst(like N, O, Fe, etc, only use element symbol not full name), the morphology
    of electrocatalyst like nanorod, nanosheet, etc. The synthesis method, stability test, N-15 labeling (it should be indicated as
    "Mentioned" if it is referenced in the content, such as in the case of experiments involving 15N isotopic labeling or 15N2/14N2 isotope
    labeling, for cases where there is no mention of N-15 labeling, "N/A" should be used).

    And some judge cretiria about the strucutre of the catalyst, synthesis methods of the catlayst,
    Judge_Nanoparticles:
    Nanoparticles typically refer to very small particles that are in the nanoscale range. These particles can be in the form of "assembl" or "dot", as well as shapes like "polyhedra", "bowl", "microsphere", "thorn", "decahedr", and more. Additionally, nanoparticles can also be described as "nano crystals", "nano particles", "nano spheres", "nano clusters", "cluster", "nano prisms", "nano cones", "nano triangles", "nano cages", "nano cubes", "nano frameworks", "nano bones", "nano dumbbells", or simply "dumbbells". Please provide the text you want to evaluate. If the text contains information related to nanoparticles, set Judge_Nanoparticles to 1. Otherwise, set it to 0.
    Judge_3D:
    Materials with three-dimensional structures have unique characteristics vital for various applications. If your text describes materials such as "hydrogel", "xerogel", "aerogel", structures like "nanostructure" or "superstructure", or terms like "monolith", "sponge", "three-dimensional", "foam", or indicates they have a "3D" configuration or are "self-supporting", then please set judge_3d to 1. Otherwise, keep it at 0.
    Judge_2D:
```

Two-dimensional materials are celebrated for their flat yet versatile nature, often found in nanotechnology. If the text mentions "nanoleaf", "lamellar", "flake", "microsheet", "two-dimensional", "nanosheet", "nanoplate", or explicitly states a "2D" nature of materials or includes "metallene", set judge_2d to 1. For all other cases, set it to 0.

Judge_1D:

One-dimensional materials, such as "tubular", "coaxial", "pillar", "nanowall", "nanoworm", "nanochain", "rod", "one-dimensional", or specifies "1D", including types like "nanotube", "nanowire", "nanorod", "nanofiber", "nanoneedle", or simple forms like "needle" or "nanobelt", are significant for their linear features. Identify these 1D materials by setting judge_1d to 1, or 0 if not mentioned.

Judge_Urchin:

Urchin structures are noted for their complex and intricate designs. Look for references to a "forest", "dendritic", "coral", "nano dendrite", "rosette", "octopus", "nano branch", "urchin", "nano flower", "flower", "nano star" within your text. Find any? Then, judge_urchin should be 1. If absent, set it to 0.

Judge_SAC:

Single-atom catalysts (SACs) represent a frontier in catalysis due to their atomic efficiency. If the text includes "single atom", "single site", mentions atomically dispersed materials, or simply refers to "SAC", you should mark judge_sac as 1. Otherwise, it stays at 0.

Judge_QD:

Quantum dots are nanoscale semiconductor particles that have quantum mechanical properties. Keywords like "0D", "quantum dot", "carbon dot", or "QD" signal the presence of these nanomaterials. Encountered these? Set judge_qd to 1, or else leave it at 0.

Judge_Hollow:

Hollow structures are interesting for their unique properties and applications in encapsulation and delivery systems. The presence of terms like "network", "interconnect", "mesoporous", "hollow", "honeycomb", "porous", or "channel" in your text points to such structures. Identify any? Then, set judge_hollow to 1. Otherwise, it remains at 0.

Judge_Core_Shell:

Core-shell structures, where one material forms a 'core' coated by another 'shell' material, are crucial in various fields, including drug delivery and sensor design. References to an "onion", "capsule", "shell", "encapsulation", "core-shell", or "yolk-shell" suggest core-shell materials are discussed. Upon finding these, judge_core_shell should be marked as 1, if not, mark it 0.

Judge_Template:

The use of a template in material science is a method for creating structured materials. If your text specifically mentions the term "template" outside contexts like "template-free", "non-template", "without template", or "no need for template", this indicates template usage. Assign judge_template as 1 in such cases, or keep it at 0 otherwise.

Judge_alloy:

Alloys and their derivatives like "metallene" or "intermetallic compounds" play a critical role in material engineering. If your text includes "alloy", "metallene", or mentions "intermetallic" compounds, it addresses alloys. Spot these terms? Set judge_alloy to 1; if not found, keep it at 0.

Judge_Functionalized:

Functionalized materials, modified or grafted for specific properties or applications, are crucial in materials science. Keywords such as "animated", "functionalized", "graft", or "modified" indicate these materials' presence. Encountered such indicators? Then assign judge_functionalized as 1. Otherwise, set it to 0.

Judge_heterostructures:

Heterostructures are systems composed of layers or regions of different materials. They are identified by terms like "nanocomposite", "hierarchical", "interface", "junction", "heterostructure", or "hybrid". Spot any of these? Indicate so by marking judge_heterostructures as 1, or leave it at 0 otherwise.

Judge_Polymer_Complex_Chelate:

Polymers, complexes, and chelates are fundamental in creating sophisticated materials with targeted functionalities. Keywords such as "Polyaniline", "Polydopamine", "polymer", "complex", "chelate" signal these materials. Found any? Move judge_polymer_complex_chelate to 1. If not, it's 0.

Judge_Biomass_Slag:

Biomass, slag, and other bio-inspired materials are gaining attention for sustainable material development. If your text discusses "bio-inspired", "natural", "vitamin", "hemin", "bacterial cellulose", "slag", "biomass", "biowaste", "waste", or "biosource", it indicates the use or study of these materials. Find such references? Then, judge_biomass_slag should be set to 1. If absent, set to 0.

Judge_MOF:

Metal-Organic Frameworks (MOFs) are versatile materials known for their exceptional porosity and customizable structures. Terms to look for include "metal-organic framework", "MOF", "zeolitic imidazolate framework", or "ZIF". Spotting any of these suggests MOFs are discussed, warranting judge_mof to be 1. Otherwise, it remains at 0.

Judge_COF:

Covalent Organic Frameworks (COFs) are crystalline porous materials with strong covalent bonds. Look for "Triazine framework", "Triazine organic framework", "COF", "covalent organic framework", or "organic framework" within the text. Presence of these indicates COFs, setting judge_cof to 1. If not mentioned, keep it at 0.

Judge_Conductive_Polymer:

Conductive polymers are key for electronic and optoelectronic applications. Relevant keywords include "Polyphenylacetylene", "Polyphenylene", "Polyaniline", "Polythiophene", "Polyacetylene", "polypyrrole". These signal conductive polymers are part of the discussion. Confirm by setting judge_conductive_polymer to 1, otherwise, it's 0.

Judge_Polymetal:

Polymetals and polyoxometalates play critical roles in catalysis and materials science. Keywords like "polyoxo", "polyoxometalates", "polymetallic", or "POMs" suggest these materials. Encountering such terms? Mark judge_polymetal as 1, or else 0.

Judge_Hydroxide_Oxyhydroxide:

Materials classified as hydroxides or oxyhydroxides are important for various chemical processes. Search for "hydroxide", "LDH", "oxyhydroxide" for their mention. Found them? Then, judge_hydroxide_oxyhydroxide must be 1. If not, it remains at 0.

Judge_oxalate:

Oxalates can play significant roles in organic and inorganic chemistry. If "oxalate" is mentioned, suggesting the presence of oxalate compounds, set judge_oxalate to 1. Otherwise, keep it at 0.

Judge_Spinel:

Spinels are a class of minerals with distinctive crystal structures, important in various fields. The mere mention of "spinel" signals its relevance to your text. Spot "spinel"? Then judge_spinel is 1, if not mentioned, it's 0.

Judge_Perovskite:

Perovskites are known for their use in solar cells and other electronic devices. If "perovskite" appears in the text, indicating discussion or analysis of these materials, judge_perovskite should be 1. Otherwise, assign it 0.

Judge_Pyrochlore:

Pyrochlore structures are notable for their application in catalysis, superconducting materials, and more. If "pyrochlore" is mentioned, it points to a focus on these structures. Identify "pyrochlore"? Set judge_pyrochlore to 1. If not, it's 0.

Judge_Prussian_Blue:

Prussian Blue and its analogs have applications in dyeing, electrochemistry, and as antidotes. If the text refers to "prussian blue", suggesting study or use of this compound, then judge_prussian_blue must be 1. Absent? Then, it's 0.

Judge_Amorphous:

Amorphous materials, lacking a clear crystalline structure, are crucial in various applications. Look for "Amorphous" or "Amorphic" to determine their discussion. Presence of these words means judge_amorphous is set to 1, otherwise, it remains at 0.

Judge_Ultrasound:

Ultrasound techniques are pivotal in various scientific and industrial processes. If terms like "ultrasonic" or "ultrasound" are mentioned, suggesting the use of ultrasound in your text, mark judge_ultrasound as 1. If these terms are absent, it should be 0.

Judge_Plasma:

Plasma processing plays a significant role in materials science for surface treatment, coating, and more. The appearance of the word "plasma" indicates discussion or application of plasma processing. Detected "plasma"? Then, judge_plasma is set to 1; if not, it remains at 0.

Judge_Graphdiyne:

Graphdiyne, a novel carbon-based material, is noteworthy for its unique properties and potential applications. Terms like "graphdiyne" or "GDY" point to the inclusion of graphdiyne. If you find these, set judge_graphdiyne to 1. In their absence, maintain it at 0.

Judge_Graphene:

Graphene, known for its extraordinary strength and conductivity, has vast applications. The mention of "graphene" clearly indicates its relevance. Encounter "graphene"? This should lead you to set judge_graphene to 1. Otherwise, it remains at 0.

Judge_c3n4:

Graphitic carbon nitride (g-C3N4) is a metal-free, polymeric photocatalyst with promising applications. Keywords to look for include "g-C3N4", "g-carbon nitride". If present, judge_c3n4 merits a 1 for identification. If these keywords are not mentioned, set it to 0.

Judge_MXene:

MXenes are a family of 2D, transition metal carbides and nitrides known for their metallic conductivity and hydrophilicity. The term "mxene" signals a discussion involving these materials. Found "mxene"? Then, assign judge_mxene as 1. No mention? Set it to 0.

Judge_CNT:

Carbon nanotubes are renowned for their strength, electrical conductivity, and thermal conductivity. Watch for "CNT", "carbon nano-tube", "carbon nano-wire", or "carbon nano-fiber". These terms confirm the presence of CNTs, warranting a judge_cnt declaration of 1. If absent, it's 0.

Judge_IL:

Ionic liquids, with their low melting point and high ionic conductivity, have diverse applications. Look for "IL", "ionic liquid" to indicate their study or application. Detect these terms? judge_il should be 1. Otherwise, it's 0.

Judge_Organic_Inorganic:

The synergy between organic and inorganic materials can lead to hybrids with enhanced properties. If the text bridges "Organic" with "Inorganic" materials, suggesting hybrid materials, then judge_organic_inorganic requires a 1. If this synergy isn't indicated, leave it at 0.

Judge_Steel:

Steel, an alloy of iron and carbon, is fundamental in construction and manufacturing. The simple inclusion of "steel" in your text signals its relevance. Is "steel" mentioned? Then, judge_steel should be set at 1. If not, it stays at 0.

Judge_Carbon_Cloth:

Carbon cloth and carbon fabric are versatile materials used in various applications from composites to filtration. Mention of "carbon cloth" or "carbon fabric" implies their discussion. Notice these? Mark judge_carbon_cloth as 1. Otherwise, it's 0.

Judge_Film:

Films, especially thin films, are crucial in electronics, coatings, and more. If the text elaborates on "film" formation or characteristics, set judge_film to 1 to acknowledge their significance. If "film" is not specifically discussed, keep it at 0.

Judge_Molten:

The molten state of materials, often critical in casting, smelting, or glass production, has unique properties. When "molten" is referenced, suggesting processes or materials in their liquid phase at high temperatures, judge_molten should be set to 1. Absent mention? It's 0.

Judge_Co_Precipitation:

Co-precipitation, a method for synthesizing nanoparticles among other applications, is indicated by terms like "co-precipitation", "impregnation", "dipping". Identify these processes or methods? Then, judge_co_precipitation is 1. Otherwise, it's 0.

Judge_Electrospin:

Electrospinning is key for producing fine, continuous fibers from polymer solutions or melts. If "electrospun" documents the fiber production process, go ahead and assign judge_electrospin as 1. If not, it stays at 0.

Judge_Hydrothermal:

Hydrothermal synthesis, commonly used in crystal growth and material fabrication, is noted with "hydrothermal", "polyol", "one-pot". Seeing these points towards hydrothermal processes involved, prompting judge_hydrothermal to be 1. No mention? Then, it's 0.

Judge_Microwave:

Microwave-assisted synthesis and processing enhance reaction rates and product yields. Keywords like "microwave" highlight these advanced methods. Found "microwave"? It's time to mark judge_microwave as 1; otherwise, the default is 0.

Judge_Spray:

Spray techniques, valuable in coating, drug delivery, and more, are captured with "spray". Its usage points to these methods being discussed. Is "spray" mentioned? Let judge_spray reflect a 1. If it's not, keep it at 0.

Judge_Phys_Chem_Deposition:

Techniques like MBE, ALD, PLD, CVD, PVD, and their variations pertain to physical or chemical vapor deposition processes crucial for thin film fabrication. If these acronyms or related terms are present, judge_phys_chem_deposition deserves a 1. Absent? Then, it's 0.

Judge_Light_Deposition:

Photochemical and light-driven deposition methods allow controlled material synthesis and processing. References to "photo", "light", or "photochemical" deposition or fabrication processes indicate their role, warranting judge_light_deposition as 1. If not discussed, it's 0.

Judge_Electrochemical_Deposition:

Electrochemical deposition is paramount in plating, coating, and material fabrication. If terms related to electrochemical processes are used, especially in the context of deposition or fabrication, then judge_electrochemical_deposition is set to 1. Otherwise, the value should be 0.

Judge_Electrochemical_Corrosion:

Electrochemical corrosion, a concern for material durability and longevity, requires attention. The presence of terms indicating corrosion or dealloying in an electrochemical context sets judge_electrochemical_corrosion to 1. If such topics are not mentioned, it remains at 0.

Judge_Etching:

Etching, whether by chemical or physical means, is integral for material patterning and fabrication. Terms like "corrosion", "etching", or "acid wash" – excluding electrochemical contexts – hint at these processes. Identified? Then judge_etching is 1. No signs of etching? It's 0.

Judge_Sol_Gel:

The sol-gel process is a versatile method for producing solid materials from small molecules. If "sol-gel" is indicated as part of the synthesis or material processing, this signifies its examination, prompting judge_sol_gel to be 1. Otherwise, you should set it to 0.

Additionally, any pertinent comments regarding the reasons for the electrocatalyst's superior performance, such as crystal facet engineering, heteroatom doping, introducing vacancies, amorphization engineering, enhanced N2 activation, or optimization of electron strucutre, etc. Answer me with several concise words that you summarized.

If there are multiple test conditions for the same catalyst,

such as different applied potential or electrolytes, and there are multiple results for faradic efficiency or NH3 yield, they

should be represented using multiple rows and keep the same columns unchanged. The table consists of 66 columns, all in lowercase.

|Catalysis Type|Reactant|Product| Faradaic efficiency | NH3 yield | Applied Potential (Faradaic efficiency) | Applied Potential (NH3 Yield) | Electrolyte | Electrocatalyst |Elements of electrocatalyst| Morphology of electrocatalyst | Synthesis Process | stability test |N-15 labeling|Judge_Nanoparticles | Judge_3D | Judge_2D | Judge_1D | Judge_Urchin | Judge_SAC | Judge_QD | Judge_Hollow | Judge_Core_Shell | Judge_Template | Judge_alloy | Judge_Functionalized | Judge_heterostructures | Judge_COF | Judge_Polymer_Complex_Chelate | Judge_Biomass_Slag | Judge_MOF | Judge_Conductive_Polymer | Judge_Polymetal | Judge_Hydroxide_Oxyhydroxide | Judge_oxalate | Judge_Spinel | Judge_Perovskite | Judge_Pyrochlore | Judge_Prussian_Blue | Judge_Amorphous | Judge_Ultrasound | Judge_Plasma | Judge_Graphdiyne | Judge_Graphene | Judge_c3n4 | Judge_MXene | Judge_CNT | Judge_IL | Judge_Organic_Inorganic | Judge_Steel | Judge_Carbon_Cloth | Judge_Film | Judge_Molten | Judge_Co_Precipitation | Judge_Electrospin | Judge_Hydrothermal | Judge_Microwave | Judge_Spray | Judge_Phys_Chem_Deposition | Judge_Light_Deposition | Judge_Electrochemical_Deposition | Judge_Electrochemical_Corrosion | Judge_Etching | Judge_Sol_Gel | Comments |

Example1:

Content: Herein, we design and synthesize two metal-porphyrin based 3D COFs bearing
metal-N4 catalytic sites with different 3D spatial arrangements (scu and flu topologies) for this purpose. The anchoring
of metal ions (Fe, Cu) at the center of the planar conjugated-porphyrin ring enhances the activity and selectivity of
electrocatalytic NRR. Significantly, the 3D COFs with Fe-N4 catalytic sites feature higher NH3 yield and Faradaic
efficiency (94.26 +/- 4.9 mu g h(-1) mg(-1) and 18.37 +/- 0.96 % at-0.5 V vs RHE, reversible hydrogen electrode) than those
with Cu-N-4 centers, making them promising candidates for NRR electrocatalysts. 15N isotopic labeling experiment confirms that nitrogen in produced
ammonia originates from N2 in the electrolyte. This work not only enriches the topological library
of 3D COFs, but also provides an idea for the rational design of metal porphyrin-based COFs for NH3 production.

Output:

|Catalysis Type |Reactant|Product| Faradaic efficiency | NH3 yield | Applied Potential (Faradaic efficiency) | Applied Potential (NH3 Yield) | Electrolyte | Electrocatalyst |Elements of electrocatalyst| Morphology of electrocatalyst | Synthesis Process | stability test |N-15 labeling| Judge_Nanoparticles | Judge_3D | Judge_2D | Judge_1D | Judge_Urchin | Judge_SAC | Judge_QD | Judge_Hollow | Judge_Core_Shell | Judge_Template | Judge_alloy | Judge_Functionalized | Judge_heterostructures | Judge_COF | Judge_Polymer_Complex_Chelate | Judge_Biomass_Slag | Judge_MOF | Judge_Conductive_Polymer | Judge_Polymetal | Judge_Hydroxide_Oxyhydroxide | Judge_oxalate | Judge_Spinel | Judge_Perovskite | Judge_Pyrochlore | Judge_Prussian_Blue | Judge_Amorphous | Judge_Ultrasound | Judge_Plasma | Judge_Graphdiyne | Judge_Graphene | Judge_c3n4 | Judge_MXene | Judge_CNT | Judge_IL | Judge_Organic_Inorganic | Judge_Steel | Judge_Carbon_Cloth | Judge_Film | Judge_Molten | Judge_Co_Precipitation | Judge_Electrospin | Judge_Hydrothermal | Judge_Microwave | Judge_Spray | Judge_Phys_Chem_Deposition | Judge_Light_Deposition | Judge_Electrochemical_Deposition | Judge_Electrochemical_Corrosion | Judge_Etching | Judge_Sol_Gel | Comments |

|Electrochemical|N2 |NH3 |18.37 +/- 0.96 % |94.26 +/- 4.9 mu g h(-1) mg(-1)|-0.5 V vs RHE |-0.5 V vs RHE |N/A |3D COFs with Fe-N4 catalytic sites|Fe, N, C |3D spatial arrangements (scu and flu topologies)|N/A |N/A |Mentioned |0 | 1 | 0 | 0 | 0 | 0 | 0 | 0 | 0 | 0 | 0 | 1 | 0 | 1 | 0 | 0 | 0 | 0 | 0 | 0 | 0 | 0 | 0 | 0 | 0 | 0 | 0 | 0 | 0 | 0 | 0 | 0 | 0 | 0 | 0 | 0 | 0 | 0 | 0 | 0 | 0 | 0 | 0 | 0 | 0 | 0 | 0 | 0 | 0 | 0 | 0 | Metal-N4 sites, 3D spatial arrangement, Fe vs. Cu activity, enhanced NRR efficiency, rational COF design|

Example2:

Content: Herein, a unique dinitrogen desorption mechanism for the electrochemical nitrogen reduction reaction is detected on the bismuth nanocrystals,
which may explain some intriguing phenomena reported in previous works, including (1) why hydrazine is still generated in some

nitrogen reduction reactions that do not follow the alternating pathway, and (2) a minor change in bismuth particle size can

lead to a dramatic change in NRR performance. In addition, the experimental and theoretical calculation results reveal that the

dinitrogen desorption pathway involving the transfer of two electrons usually shows lower energy barriers and faster reaction

kinetics, resulting in a dramatic enhancement in NRR performance.

Output:

|Catalysis Type |Reactant|Product| Faradaic efficiency | NH3 yield | Applied Potential (Faradaic efficiency) | Applied Potential (NH3 Yield) | Electrolyte | Electrocatalyst |Elements of electrocatalyst| Morphology of electrocatalyst | Synthesis Process | stability test |N-15 labeling|Judge_Nanoparticles | Judge_3D | Judge_2D | Judge_1D | Judge_Urchin | Judge_SAC | Judge_QD | Judge_Hollow | Judge_Core_Shell | Judge_Template | Judge_alloy | Judge_Functionalized | Judge_heterostructures | Judge_COF | Judge_Polymer_Complex_Chelate | Judge_Biomass_Slag | Judge_MOF | Judge_Conductive_Polymer | Judge_Polymetal | Judge_Hydroxide_Oxyhydroxide | Judge_oxalate | Judge_Spinel | Judge_Perovskite | Judge_Pyrochlore | Judge_Prussian_Blue | Judge_Amorphous | Judge_Ultrasound | Judge_Plasma | Judge_Graphdiyne | Judge_Graphene | Judge_c3n4 | Judge_MXene | Judge_CNT | Judge_IL | Judge_Organic_Inorganic | Judge_Steel | Judge_Carbon_Cloth | Judge_Film | Judge_Molten | Judge_Co_Precipitation | Judge_Electrospin | Judge_Hydrothermal | Judge_Microwave | Judge_Spray | Judge_Phys_Chem_Deposition | Judge_Light_Deposition | Judge_Electrochemical_Deposition | Judge_Electrochemical_Corrosion | Judge_Etching | Judge_Sol_Gel | Comments|

|Electrochemical|N2 |NH3 |N/A |N/A |N/A |N/A
|N/A |bismuth |Bi |nanocrystals |N/A |N/A |N/A |1
| 0 | 0 | 0 | 0 | 0 | 0 | 0 | 0 | 0 | 0 | 0 | 0
| 0 | 0 | 0 | 0 | 0 | 0 | 0 | 0 | 0
| 0 | 0 | 0 | 0 | 0 | 0 | 0 | 0 | 0 | 0 |
0 | 0 | 0 | 0 | 0 | 0 | 0 | 0 | 0 | 0
| 0 | 0 | 0 | 0 | 0 | 0 | 0 | 0
| particle size impact, two-electron transfer, enhanced NRR kinetics|

Example3:

Content: In this study, an innovative strategy is proposed to protect fresh black

phosphorene (BPene) against oxidation by adsorbing or weakly bonding aluminum ions (Al3+) onto its surface, while

facilitating their self-removal under operating conditions. By leveraging Al3+-protected black phosphorene (Al-BPene)

as a catalyst for the electrocatalytic reduction of nitrogen to ammonia (NH3), the effectiveness of this removable

Al3+-protected strategy is demonstrated. Quantitative analysis reveals that Al-BPene maintains structural and

compositional stability for over 7 days under ambient conditions, while the Al3+ can be readily eliminated through

self-desorption or dissolution in the electrolyte. With highly active sites pre-protected, Al-BPene exhibits a striking

NH3 yield rate of 55.1 mu g h-1 mg cat-1 and a Faradaic efficiency (FE) of 66.5% in alkaline media, as exemplified by the

representative application example. Moreover, it is found that the prepared Al-BPene can tolerate a thermal environment,

and a higher NH3 yield rate of 75 mu g h-1 mg cat-1 at 60 degrees C is recorded. Undoubtedly, these findings pave the way

for broader applications of BPene. A removable aluminum ion protection strategy is proposed to protect black phosphorene

against oxidation under ambient conditions, while facilitating the self-removal of aluminum to regenerate fresh black

phosphorene with enhanced reactivity under operating conditions. As exemplified by a representative application, this

approach enables black phosphorene to exhibit an impressive Faradaic efficiency of 66.5% for ammonia electrosynthesis.

The 15N2/14N2 isotope labeling experiments and density functional theory (DFT) calculations showed that N2 can be adsorbed and

converted to NH3 on the surface of BCN.

Output:

|Catalysis Type |Reactant|Product| Faradaic efficiency | NH3 yield | Applied Potential (Faradaic efficiency) | Applied Potential (NH3 Yield) | Electrolyte | Electrocatalyst |Elements of electrocatalyst| Morphology of electrocatalyst | Synthesis Process | stability test |N-15 labeling| Judge_Nanoparticles | Judge_3D | Judge_2D | Judge_1D | Judge_Urchin | Judge_SAC | Judge_QD | Judge_Hollow | Judge_Core_Shell | Judge_Template | Judge_alloy | Judge_Functionalized | Judge_heterostructures | Judge_COF | Judge_Polymer_Complex_Chelate | Judge_Biomass_Slag | Judge_MOF | Judge_Conductive_Polymer | Judge_Polymetal | Judge_Hydroxide_Oxyhydroxide | Judge_oxalate | Judge_Spinel | Judge_Perovskite | Judge_Pyrochlore | Judge_Prussian_Blue | Judge_Amorphous | Judge_Ultrasound | Judge_Plasma | Judge_Graphdiyne | Judge_Graphene | Judge_c3n4 | Judge_MXene | Judge_CNT | Judge_IL | Judge_Organic_Inorganic | Judge_Steel | Judge_Carbon_Cloth | Judge_Film | Judge_Molten | Judge_Co_Precipitation | Judge_Electrospin | Judge_Hydrothermal | Judge_Microwave | Judge_Spray | Judge_Phys_Chem_Deposition | Judge_Light_Deposition | Judge_Electrochemical_Deposition | Judge_Electrochemical_Corrosion | Judge_Etching | Judge_Sol_Gel | Comments |

|Electrochemical|N2 |NH3 |66.5% |55.1 mu g h-1 mg cat-1|N/A |N/A |alkaline media|Al3+-protected black phosphorene (Al-BPene)|Al, P |nanocrystals |N/A |over 7 days |Mentioned | 0 | 0 | 1 | 0 | 0 | 0 | 0 | 0 | 0 | 0 | 0 | 1 | 0 | 0 | 0 | 0 | 0 | 0 | 0 | 0 | 0 | 0 | 0 | 0 | 0 | 0 | 0 | 0 | 0 | 0 | 0 | 0 | 0 | 0 | 0 | 0 | 0 | 0 | 0 | 0 | 0 | 0 | 0 | 0 | 0 | 0 | 0 | 0 | 0 | 0 | Al3+ protection, black phosphorene stability, enhanced NH3 yield, thermal tolerance, self-desorption mechanism|

|Electrochemical|N2 |NH3 |N/A |75 mu g h-1 mg cat-1 at 60 degrees C|N/A |N/A |N/A |Al3+-protected black phosphorene (Al-BPene)|Al, P |nanocrystals |N/A |N/A |Mentioned |0 | 0 | 1 | 0 | 0 | 0 | 0 | 0 | 0 | 0 | 0 | 0 | 1 | 0 | 0 | 0 | 0 | 0 | 0

| 0 | 0 | 0 | 0 | 0 | 0 | 0 | 0 | 0
| 0 | 0 | 0 | 0 | 0 | 0 | 0 | 0 | 0 | 0 | 0
| 0 | 0 | 0 | 0 | 0 | 0 | 0 | 0 | 0
| 0 | 0 | 0 | Al3+ protection, black phosphorene stability, enhanced NH3 yield, thermal tolerance, self-desorption mechanism|

Example4:

Content: Here, we report an efficient electrocatalyst synthesized via a simple self-assembly process from supramolecular cucurbit[6]uril (Q[6])

and a heteropolyacid for nitrogen oxidation. The as-prepared self-assembly material Q[6]-PMA with high stability and specific surface

area shows a prominent activity in the electrocatalytic nitrogen oxidation reaction (NOR), affording a yield of nitrate (NO3-) of up to

271.5 +/- 5.6 mu g h-1 mgcat-1 with a Faradaic efficiency (FE) of 71.2 +/- 3.8%. The 15N isotope-labeling experiment confirmed that the

nitrogen source of nitrate formation is derived from N2. The results of the in situ XPS technique and in situ FITR experiments demonstrate

that the molybdenum center of Q[6]-PMA is the main active site of the NOR and the corresponding cucurbit[6]uril unit is conducive to the

stability of key intermediates. A supramolecular self-assembler of cucurbit[6]uril-phosphomolybdic acid with a multi-valence state is

constructed to realize an efficient nitrogen oxidation reaction.

Output:

|Catalysis Type |Reactant|Product| Faradaic efficiency | NH3 yield | Applied Potential (Faradaic efficiency) | Applied Potential (NH3 Yield) | Electrolyte | Electrocatalyst |Elements of electrocatalyst| Morphology of electrocatalyst | Synthesis Process | stability test |N-15 labeling| Judge_Nanoparticles | Judge_3D | Judge_2D | Judge_1D | Judge_Urchin | Judge_SAC | Judge_QD | Judge_Hollow | Judge_Core_Shell | Judge_Template | Judge_alloy | Judge_Functionalized | Judge_heterostructures | Judge_COF | Judge_Polymer_Complex_Chelate | Judge_Biomass_Slag | Judge_MOF | Judge_Conductive_Polymer | Judge_Polymetal | Judge_Hydroxide_Oxyhydroxide | Judge_oxalate | Judge_Spinel | Judge_Perovskite | Judge_Pyrochlore | Judge_Prussian_Blue | Judge_Amorphous | Judge_Ultrasound | Judge_Plasma | Judge_Graphdiyne | Judge_Graphene | Judge_c3n4 | Judge_MXene | Judge_CNT | Judge_IL | Judge_Organic_Inorganic | Judge_Steel | Judge_Carbon_Cloth | Judge_Film | Judge_Molten | Judge_Co_Precipitation | Judge_Electrospin | Judge_Hydrothermal | Judge_Microwave | Judge_Spray | Judge_Phys_Chem_Deposition | Judge_Light_Deposition | Judge_Electrochemical_Deposition | Judge_Electrochemical_Corrosion | Judge_Etching | Judge_Sol_Gel |Comments |

|Electrochemical|N2 |NO3- |71.2 +/- 3.8% |271.5 +/- 5.6 mu g h-1 mgcat-1|N/A |N/A |N/A |Q[6]-PMA |Mo |N/A |a simple self-assembly process|N/A |Mentioned | 0 | 0 | 0 | 0 | 0 | 0 | 0 | 0
| 0 | 1 | 0 | 1 | 0 | 0 | 0 | 0 | 0 | 0 | 1 | 0 | 0 | 0 | 0 | 0 | 0 | 0
| 0 | 0 | 0 | 0 | 0 | 0 | 0 | 0 | 0 | 0 | 0
| 0 | 0 | 0 | 0 | 0 | 0 | 0 | 0 | 0
| 0 | 0 | 0 | 0 | N/A |

Example5:

Content: This paper reports the synthesis of a NiO loaded KNbO3 nanocomposite via a photodeposition method and its application in photocatalytic N-2 fixation for the first time.

Results indicated that the NiO/KNbO3 composite displayed superior photocatalytic efficiency in NH3 production under simulated sunlight illumination. The optimal NiO/KNbO3 sample

showed an NH3-generation rate of 470.6 mu mol g(-1) h(-1), which reaches 4.8 times that of pristine KNbO3. Various characterization techniques including powder X-ray diffraction (XRD),

Raman, X-ray photoelectron spectroscopy (XPS), UV-visible diffuse reflection spectroscopy (DRS), transient photocurrent, electrochemical impedance spectroscopy (EIS), and linear sweep

voltammetry (LSV) were applied to reveal the mechanism behind the high photoactivity of NiO/KNbO3. The addition of NiO nanoparticles showed a slight effect on the specific surface area

and the optical properties of KNbO3, but elevated the charge separation efficiency which was verified to be the key role in affecting the photocatalytic reaction. The introduced NiO

fabricated a p-n heterojunction structure with KNbO3 and hence achieved enhanced efficiency in separating electrons and holes via a type-II mechanism. The cycling test further demonstrated

that the NiO/KNbO3 sample has high photocatalytic stability, indicating its good potential in photocatalytic nitrogen fixation.

Output:

|Catalysis Type |Reactant|Product| Faradaic efficiency | NH3 yield | Applied Potential (Faradaic efficiency) | Applied Potential (NH3 Yield) | Electrolyte | Electrocatalyst |Elements of electrocatalyst| Morphology of electrocatalyst | Synthesis Process | stability test | N-15 labeling|Judge_Nanoparticles | Judge_3D | Judge_2D | Judge_1D | Judge_Urchin | Judge_SAC | Judge_QD | Judge_Hollow | Judge_Core_Shell | Judge_Template | Judge_alloy | Judge_Functionalized | Judge_heterostructures | Judge_COF | Judge_Polymer_Complex_Chelate | Judge_Biomass_Slag | Judge_MOF | Judge_Conductive_Polymer | Judge_Polymetal | Judge_Hydroxide_Oxyhydroxide | Judge_oxalate | Judge_Spinel | Judge_Perovskite | Judge_Pyrochlore | Judge_Prussian_Blue | Judge_Amorphous | Judge_Ultrasound | Judge_Plasma | Judge_Graphdiyne | Judge_Graphene | Judge_c3n4 | Judge_MXene | Judge_CNT | Judge_IL | Judge_Organic_Inorganic | Judge_Steel | Judge_Carbon_Cloth | Judge_Film | Judge_Molten | Judge_Co_Precipitation | Judge_Electrospin | Judge_Hydrothermal | Judge_Microwave | Judge_Spray | Judge_Phys_Chem_Deposition | Judge_Light_Deposition | Judge_Electrochemical_Deposition | Judge_Electrochemical_Corrosion | Judge_Etching | Judge_Sol_Gel |Comments |

|Phoelectrochemical|N2 |NH3 |N/A |470.6 mu mol g(-1) h(-1)|N/A |N/A |N/A |NiO/KNbO3 |Ni, O, K, Nb |N/A |Photodeposition method|High photocatalytic stability|N/A | 1 | 1 | 0 | 0 | 0 | 0 | 0 | 0 | 0 | 0 | 0 | 1 | 1 | 0 | 0 | 0 | 0 | 0 | 0 | 0 | 0 | 0 | 0 | 0 | 0 | 0 | 0 | 0 | 0 | 0 | 0 | 0 | 0 | 0 | 0 | 0 | 0 | 0 | 0 | 0 | 0 | 0 | 0 | 0 | 1 | 0 | 0 | 0 | 0 | 0 |enhanced charge separation|

Example6:

Content: Control synthesis of self-standing porous bimetallic nanosheet array is of significant importance for electrocatalytic applications. Herein, we report an in-situ approach for directly

fabricating interweaved porous palladium-ruthenium nanosheets on Ni foam (PdRu NS-NF) via a micelle-assisted galvanic replacement reaction using Ni foam as both the reductant and substrate.

These nanosheets possess interconnected porosity and bimetallic compositions, which provide abundant active sites and strong electronic effect. The binder-free PdRu NS-NF shows outstanding

activity and stability for electrocatalytic nitrogen reduction to ammonia. Different from traditional complex methods for the fabrication of metallic nanosheets, the proposed approach is

very feasible to directly synthesize metallic array with tunable morphology and composition on three-dimensional metallic substrates.

Output:

|Catalysis Type |Reactant|Product| Faradaic efficiency | NH3 yield | Applied Potential (Faradaic efficiency) | Applied Potential (NH3 Yield) | Electrolyte | Electrocatalyst |Elements of electrocatalyst| Morphology of electrocatalyst | Synthesis Process | stability test |N-15 labeling| Judge_Nanoparticles | Judge_3D | Judge_2D | Judge_1D | Judge_Urchin | Judge_SAC | Judge_QD | Judge_Hollow | Judge_Core_Shell | Judge_Template | Judge_alloy | Judge_Functionalized | Judge_heterostructures | Judge_COF | Judge_Polymer_Complex_Chelate | Judge_Biomass_Slag | Judge_MOF | Judge_Conductive_Polymer | Judge_Polymetal | Judge_Hydroxide_Oxyhydroxide | Judge_oxalate | Judge_Spinel | Judge_Perovskite | Judge_Pyrochlore | Judge_Prussian_Blue | Judge_Amorphous | Judge_Ultrasound | Judge_Plasma | Judge_Graphdiyne | Judge_Graphene | Judge_c3n4 | Judge_MXene | Judge_CNT | Judge_IL | Judge_Organic_Inorganic | Judge_Steel | Judge_Carbon_Cloth | Judge_Film | Judge_Molten | Judge_Co_Precipitation | Judge_Electrospin | Judge_Hydrothermal | Judge_Microwave | Judge_Spray | Judge_Phys_Chem_Deposition | Judge_Light_Deposition | Judge_Electrochemical_Deposition | Judge_Electrochemical_Corrosion | Judge_Etching | Judge_Sol_Gel |Comments |

|Electrochemical|N2 |NH3 |N/A |N/A |N/A |N/A |N/A |PdRu NS-NF |Pd, Ru, Ni |interweaved porous palladium-ruthenium nanosheets|micelle-assisted galvanic replacement reaction|outstanding activity and stability |N/A |0 | 0 | 1 | 0 | 0 | 0 | 0 | 1 | 0 | 0 | 1 | 1 | 0 | 0 | 0 | 0 | 0 | 0 | 0 | 0 | 0 | 0 | 0 | 0 | 0 | 0 | 0 | 0 | 0 | 0 | 0 | 0 | 0 | 0 | 0 | 0 | 0 | 0 | 0 | 0 | 0 | 0 | 0 | 0 | 0 | 0 | 0 | 0 | 0 | 1 | 0 | 0 | 0 | interconnected porosity, electronic effect|

Example7:

Content: The electrochemical nitrogen reduction reaction (NRR) is an attractive process for next-generation ammonia synthesis; therefore,

identifying a suitable catalyst for this reaction is critical. In recent years, transition-metal dichalcogenides (TMDs) and their

Janus structures have gained significant attention because of their outstanding catalytic properties. However, the synthesis of

Janus TMDs remains challenging, and exposing their active sites is difficult when using a low-dimensional structure to improve the

catalytic activity. To date, relatively little research has been conducted in this area. Herein, emerging Janus WSeS/WSe2 heterostructure

```
nanowalls are systematically explored. These nanowalls are used as a nitrogen fixation catalyst in electrolytes. The nanowalls demonstrate
a significant NH3 yield rate and Faradaic efficiency of 13.97 mu g h-mgcat(-1 )and 35.24% at -0.3 V in 0.1 m HCl, as well as
15.96 mu g h-mgcat(-1) and 40.2% in 0.1 M Na2SO4. This study presents an in-depth analysis of the properties of Janus WSeS/WSe2 heterostructure
nanowalls and a conceptual framework for linking TMD-based catalysts and the NRR.
Output:
|Catalysis Type |Reactant|Product| Faradaic efficiency | NH3 yield | Applied Potential (Faradaic efficiency) | Applied Potential (NH3 Yield) | Electrolyte | Electrocatalyst |Elements of electrocatalyst| Morphology of electrocatalyst | Synthesis Process | stability test |N-15 labeling| Judge_Nanoparticles | Judge_3D | Judge_2D | Judge_1D | Judge_Urchin | Judge_SAC | Judge_QD | Judge_Hollow | Judge_Core_Shell | Judge_Template | Judge_alloy | Judge_Functionalized | Judge_heterostructures | Judge_COF | Judge_Polymer_Complex_Chelate | Judge_Biomass_Slag | Judge_MOF | Judge_Conductive_Polymer | Judge_Polymetal | Judge_Hydroxide_Oxyhydroxide | Judge_oxalate | Judge_Spinel | Judge_Perovskite | Judge_Pyrochlore | Judge_Prussian_Blue | Judge_Amorphous | Judge_Ultrasound | Judge_Plasma | Judge_Graphdiyne | Judge_Graphene | Judge_c3n4 | Judge_MXene | Judge_CNT | Judge_IL | Judge_Organic_Inorganic | Judge_Steel | Judge_Carbon_Cloth | Judge_Film | Judge_Molten | Judge_Co_Precipitation | Judge_Electrospin | Judge_Hydrothermal | Judge_Microwave | Judge_Spray | Judge_Phys_Chem_Deposition | Judge_Light_Deposition | Judge_Electrochemical_Deposition | Judge_Electrochemical_Corrosion | Judge_Etching | Judge_Sol_Gel |Comments |
|Electrochemical|N2 |NH3 |35.24% |13.97 mu g h-mgcat(-1)|-0.3 V |-0.3 V |0.1 m HCl |Janus WSeS/WSe2 heterostructure |W, Se, S |nanowalls
|N/A |N/A |N/A | 1 | 0 | 1 | 0 | 0 | 0 | 0 | 0 | 0
| 0 | 0 | 1 | 1 | 0 | 0 | 0 | 0 | 0
| 0 | 0 | 0 | 0 | 0 | 0 | 0 | 0 | 0
| 0 | 0 | 0 | 0 | 0 | 0 | 0 | 0 | 0 | 0 | 0
| 0 | 0 | 0 | 0 | 0 | 0 | 0 | 0 | 0
| 0 | 0 | 0 | N/A |
|Electrochemical|N2 |NH3 |40.2% |15.96 mu g h-mgcat(-1)|N/A |N/A
|0.1 M Na2SO4 |Janus WSeS/WSe2 heterostructure |W, Se, S | nanowalls |N/A
|N/A |N/A | 1 | 0 | 1 | 0 | 0 | 0 | 0 | 0 | 0 | 0
| 0 | 1 | 1 | 0 | 0 | 0 | 0 | 0 | 0
| 0 | 0 | 0 | 0 | 0 | 0 | 0 | 0 | 0 |
0 | 0 | 0 | 0 | 0 | 0 | 0 | 0 | 0 | 0 | 0 |
0 | 0 | 0 | 0 | 0 | 0 | 0 | 0
| 0 | 0 | 0 | N/A |
"""
user_ending = f"Please provide the extracted information for the following paper: {column1_value} \n\nContext:\n{context}, If any information is not provided or you are unsure, use 'N/A'."
```

10. **The script for transforming ChatGPT output into a clean data frame**

```
def tabulate_condition(df, column_name):
    """This function converts the text from a ChatGPT conversation into a DataFrame.
    It also cleans the DataFrame by dropping additional headers and empty lines.    """

    table_text = df[column_name].str.cat(sep='\n')

    # Remove leading and trailing whitespace
    table_text = table_text.strip()

    # Split the table into rows
    rows = table_text.split('\n')

    # Extract the header row and the divider row
    header_row, *data_rows = rows

    # Extract column names from the header row

    column_names = ['Catalysis Type', 'Reactant', 'Product', 'Faradaic efficiency', 'NH3 yield', 'Applied Potential
(Faradaic efficiency)', 'Applied Potential (NH3 Yield)',
                    'Electrolyte', 'Electrocatalyst', 'Elements of electrocatalyst', 'Morphology of electrocatalyst', 'Synthesis
Process', 'stability test', 'N-15 labeling',
                    'Judge_Nanoparticles', 'Judge_3D', 'Judge_2D', 'Judge_1D', 'Judge_Urchin', 'Judge_SAC', 'Judge_QD',
'Judge_Hollow', 'Judge_Core_Shell', 'Judge_Template',
                    'Judge_alloy', 'Judge_Functionalized', 'Judge_heterostructures', 'Judge_COF',
'Judge_Polymer_Complex_Chelate', 'Judge_Biomass_Slag', 'Judge_MOF', 'Judge_Conductive_Polymer',
                    'Judge_Polymetal', 'Judge_Hydroxide_Oxyhydroxide', 'Judge_oxalate', 'Judge_Spinel',
'Judge_Perovskite', 'Judge_Pyrochlore', 'Judge_Prussian_Blue', 'Judge_Amorphous',
                    'Judge_Ultrasound', 'Judge_Plasma', 'Judge_Graphdiyne', 'Judge_Graphene', 'Judge_c3n4',
'Judge_MXene', 'Judge_CNT', 'Judge_IL', 'Judge_Organic_Inorganic', 'Judge_Steel',
                    'Judge_Carbon_Cloth', 'Judge_Film', 'Judge_Molten', 'Judge_Co_Precipitation', 'Judge_Electrospin',
'Judge_Hydrothermal', 'Judge_Microwave', 'Judge_Spray',
                    'Judge_Phys_Chem_Deposition', 'Judge_Light_Deposition', 'Judge_Electrochemical_Deposition',
'Judge_Electrochemical_Corrosion', 'Judge_Etching', 'Judge_Sol_Gel', 'Comments']

    # Create a list of dictionaries to store the table data
    data = []

    # Process each data row
    for row in data_rows:

        # Split the row into columns
        columns = [col.strip() for col in row.split('|') if col.strip()]
```

```
    # Create a dictionary to store the row data
    row_data = {col_name: col_value for col_name, col_value in zip(column_names, columns)}

    # Append the dictionary to the data list
    data.append(row_data)

df = pd.DataFrame(data)



"""Make df clean by drop additional header and empty lines """
def contains_pattern(s, patterns):
    return any(re.search(p, s) for p in patterns)

def drop_rows_with_patterns(df, column_name):
    #empty cells, N/A cells and header cells
    patterns = [r'^\s*$', r'--',r'-\s-', r'electrocatalyst', r'Electrocatalyst',r'Catalyst', r'catalyst',
            r'NaN',r'N/A',r'n/a',r'\nN/A', r'note', r'Note']

    mask = df[column_name].apply(lambda x: not contains_pattern(str(x), patterns))
    filtered_df = df[mask]

    return filtered_df

#drop the repeated header
df = drop_rows_with_patterns(df, 'Electrocatalyst')

# drop the N/A rows
filtered_df = df.dropna(subset=['Electrocatalyst'])

return filtered_df
```

11. The script for automated classification of electrolyte pH levels

```
def classify_electrolyte(row):
    electrolyte = row['Electrolyte']
    ph = row['pH']

    if pd.isnull(electrolyte):
        return ph

    if any(keyword in electrolyte.lower() for keyword in ['liclo4', 'neutral', 'pbs', 'licl']):
        return 'neutral'
    elif any(keyword in electrolyte.lower() for keyword in ['na2so4', 'k2so4', 'li2so4']):
        return 'weak acid'
    elif any(keyword in electrolyte.lower() for keyword in ['koh', 'naoh', 'alkaline']):
        return 'alkaline'
    elif 'khco3' in electrolyte.lower():
        return 'weak alkaline'
    elif any(keyword in electrolyte.lower() for keyword in ['hcl', 'h2so4', 'acidic']):
        return 'acidic'
    else:
        return electrolyte

table['pH'] = table.apply(classify_electrolyte, axis=1)
```

12. Automatically construct catalyst structure descriptors using ChatGPT

System: You are a helpful assistant. You can help me by answering my questions. You can also ask me questions.

User: If you are an expert familiar with material structures, based on the following descriptions of the structures I provide, could you roughly categorize them? Please assign each structure to a category and provide the reason, in a format like: A type1 Reason.

Assistant: Of course, I'd be happy to help categorize the material structures based on your descriptions. Please provide the descriptions of the structures, and I will categorize them accordingly.

User: My data:
No values
distributed on graphite paper
nanodots
nanodots
Nanoparticles
No values
carbon mat
nanosheet-like
hollow carbon spheres …

Assistant: Based on the provided data and descriptions of the structures, I will categorize them

into general groups such as nanoparticles, nanorods, nanowires, nanosheets, nanofibers, nanocubes, hollow structures, porous structures, and hybrids. Here are the categorizations:

Nanoparticles:

Nanoparticles

Nanoparticles/C

…

Reason: These descriptions specifically mention "nanoparticles" or equivalent terms such as "nanodots" or "quantum dots."

Nanorods:

Nanorods

Nanorod array

…

Reason: These descriptions specifically mention "nanorods" or equivalent terms.

Nanowires:

Nanowires

Nanowire arrays

…

Reason: These descriptions specifically mention "nanowires" or equivalent terms.

…

User: please give me a Python script, I can add a "structure type" column according to "Morphology of electrocatalyst" column

Assistant: Sure! Below is a Python script that uses the pandas library to add a "Structure Type" column to a DataFrame based on the "Morphology of electrocatalyst" column. This script includes a mapping of various morphology descriptors to their corresponding structure types.

You need to have pandas installed. You can install it using pip if you haven't already: pip install pandas. Here is the Python script:

```
import pandas as pd

# Sample data
data = {
    'Morphology of electrocatalyst': [
        'nanoparticles', 'nanorods', 'nanowires', 'nanosheets', 'nanofibers',
        'nanocubes', 'hollow carbon spheres', 'porous hybrid', 'nanoparticle-reduced graphene oxide hybrid',
        'nanotubes wrapped by 2d graphene sheets', 'nanowalls'
    ]
}

# Create DataFrame
df = pd.DataFrame(data)

# Function to categorize morphology
def categorize_structure(morphology):
    if 'nanoparticle' in morphology.lower() or 'quantum dots' in morphology.lower() or 'nanodots' in
```

```
morphology.lower():
        return 'Nanoparticles'
    elif 'nanorod' in morphology.lower():
        return 'Nanorods'
    elif 'nanowire' in morphology.lower():
        return 'Nanowires'
    elif 'nanosheet' in morphology.lower() or '2d' in morphology.lower():
        return 'Nanosheets'
    elif 'nanofiber' in morphology.lower():
        return 'Nanofibers'
    elif 'nanocube' in morphology.lower():
        return 'Nanocubes'
    elif 'hollow' in morphology.lower():
        return 'Hollow Structures'
    elif 'porous' in morphology.lower():
        return 'Porous Structures'
    elif 'hybrid' in morphology.lower() or 'composite' in morphology.lower():
        return 'Hybrids and Composites'
    elif 'nanotube' in morphology.lower():
        return 'Nanotubes'
    elif 'nanowall' in morphology.lower():
        return 'Nanowalls'
    else:
        return 'Other'

# Add 'Structure Type' column
df['Structure Type'] = df['Morphology of electrocatalyst'].apply(categorize_structure)

# Display the DataFrame
print(df)
```

13. Methodology for Knowledge Graph Construction with GraphRAG

This section provides a detailed methodology for the construction of the knowledge graph used by the GraphRAG retriever agent in eNRRCrew. The process transforms structured tabular data (CSV) into a rich, interconnected graph, enabling sophisticated querying of the eNRR literature and catalyst system recommendations. The pipeline is orchestrated by a central configuration file (*settings.yaml*) and guided by a series of structured prompts.

1. Graph Transformation from Tabular Data

Input Configuration: The pipeline is configured via *settings.yaml* to process CSV files. It identifies a *text_column* (e.g., "Abstract") containing the natural language text to be analyzed and a *source_column* (e.g., "DOI") that serves as a

unique identifier for each data record.

Text Chunking: For each row in the table, the text from the *text_column* is read and segmented into smaller, overlapping chunks. This ensures that semantic context is preserved at the boundaries of each chunk, which is critical for accurate relationship extraction.

Entity and Relationship Extraction: This is the core transformation step, where a Large Language Model (LLM) is prompted with the instructions in *prompts/entity_extraction.txt*. For each text chunk, the LLM performs two key tasks: It identifies and extracts named entities that conform to the predefined schema (see Section S13.2). It analyzes the text to identify and describe the relationships connecting these entities, assigning a confidence score to each relationship.

Graph Assembly: The entities and relationships extracted from all text chunks are aggregated to form a single, unified knowledge graph. This graph is then stored as a *.graphml* file, a standard and portable format, as specified by the *snapshots: graphml: true* setting in the *settings.yaml*.

2. Graph Schema: Entities and Relationships

The schema defines the types of information stored in the graph and is explicitly defined in the *prompts/entity_extraction.txt* file.

Entity Types: The LLM is instructed to extract entities belonging to a specific list of types highly relevant to eNRR research: *chemical element*, *electrocatalyst*, *synthesis method*, *morphology*, *reaction condition*, *applied potential*, *pH level*, *electrolyte type*, *NH_3 yield*, *faradaic efficiency*.

Relationship Types: Relationships are not defined by a fixed list but are generated dynamically by the LLM. The prompt instructs the model to identify pairs of "clearly related" entities and generate a *relationship_description* in natural language that explains the connection, along with a *relationship_strength* score from 1 to 10. For example, a relationship might be described as: "The applied potential of -0.5 V vs RHE is used for the electrochemical nitrogen reduction reaction catalyzed by 3D COFs with $Fe-N_4$." This dynamic approach allows for the capture of nuanced and complex interactions directly from the source text.

3. Data Storage Architecture

The pipeline employs a sophisticated storage strategy to manage the graph and its associated data efficiently.

Graph Structure Storage: The primary graph structure, containing all nodes and edges, is saved in GraphML format within the *output/<timestamp>/artifacts/ directory*.

Vector Embeddings Storage (LanceDB): After entities are extracted, their descriptions are summarized and converted into high-dimensional vector embeddings using a sentence-transformer model (*text-embedding-3-small* in our

case). These vectors are stored in LanceDB, a high-performance vector database. This enables efficient semantic search and similarity lookups, which are fundamental to the GraphRAG retrieval process.

4. Entity Management: Naming, Disambiguation, and Updates

The pipeline includes robust mechanisms to ensure the consistency and quality of the entity data.

Entity Naming: Entity naming is handled by the LLM, which is instructed to capitalize entity names for standardization.

Entity Disambiguation and Summarization: This critical step is managed by the *summarize_descriptions* workflow. Often, the same entity is mentioned multiple times across the dataset, resulting in several descriptive snippets. The pipeline tasks the LLM (using *prompts/summarize_descriptions.txt*) to synthesize these varied descriptions into a single, coherent summary. Crucially, the prompt instructs the model: "If the provided descriptions are contradictory, please resolve the contradictions and provide a single, coherent summary." This process effectively disambiguates entities and creates a canonical, consolidated description for each node in the graph.

Incremental Updates: The GraphRAG pipeline supports efficient incremental updates, allowing users to append new data without rebuilding the entire graph. By configuring input and update storage paths and placing new data into the input folder, the system efficiently updates entities, relationships, and communities with minimal re-computation. This ensures the knowledge graph can be maintained and scaled efficiently as new research emerges.

14. 13 recommended catalysts, reaction conditions, and performance evaluation by eNRRCrew

14.1 A novel ammonia electrocatalyst system may be designed based on a hetero-diatomic catalyst incorporating vanadium (V) and chromium (Cr) atoms anchored on a two-dimensional porous graphitic carbon nitride (g-CN) nanosheet substrate to maximize active site exposure and facilitate mass transport. The catalyst shall operate at an electrode potential around -0.2 V vs RHE in a neutral pH electrolyte (~7), such as phosphate-buffered saline with 0.1 M K_2SO_4, to balance nitrogen reduction and suppress competing hydrogen evolution reactions while maintaining catalyst stability. The synthesis could involve chemical vapor deposition to ensure uniform dispersion of V and Cr atoms, leveraging the synergistic effects of dual metal sites and transition metal doping to enhance nitrogen adsorption and activation, aiming for high ammonia yield and faradaic efficiency. This system differs from previously reported catalysts by combining V-Cr dual-atom sites on g-CN with optimized reaction conditions, offering a promising new approach for efficient electrochemical nitrogen reduction. *Predictions: Yield: high; FE: low.*

14.2 A novel ammonia electrocatalyst system may be designed based on a bimetallic Mo-Co dimer structure atomically dispersed on a porous nitrogen-doped graphene matrix. The catalyst morphology shall consist of atomically dispersed Mo-Co dimers embedded within the conductive, defect-rich graphene to maximize nitrogen adsorption and reaction kinetics. The reaction conditions may involve an acidic electrolyte to maintain proton availability and suppress competing hydrogen evolution reactions, with an applied potential near 0.27 V vs RHE to optimize ammonia yield and Faradaic efficiency. *Predictions: Yield: high; FE: low.*

14.3 A dual-metal single-atom catalyst incorporating Fe and Mo sites on boron-doped carbon nitride nanosheets could be employed, operating at around -0.3 V vs RHE in neutral pH 6 using a 0.1 M Na_2SO_4 electrolyte to balance catalyst stability and nitrogen adsorption. *Predictions: Yield: high; FE: low.*

14.4 Another promising design involves Mo and W single atoms dispersed on a Ti_2NO_2 MXene substrate with defect engineering to enhance nitrogen activation, operating at approximately -0.27 V vs RHE in neutral conditions (pH 6) with buffered electrolytes such as 0.1 M HCl. These systems leverage synergistic bimetallic or dual-metal active sites, conductive 2D supports, and optimized reaction environments to achieve high catalytic performance and selectivity for nitrogen reduction, representing innovative configurations not previously reported. *Predictions: Yield: high; FE: high.*

14.5 A novel ammonia electrocatalyst system could be designed based on a bimetallic Mo-Co dimer structure atomically dispersed on a high surface area nitrogen-doped graphene (N-doped graphene) substrate, combining the high catalytic activity of Mo-Co dimers with the excellent electron transfer and stability of N-doped graphene. This catalyst would operate under acidic conditions to enhance proton availability, with an optimized electrode potential around -0.35 V vs RHE to balance nitrogen reduction and suppress competing reactions. The morphology involves atomically dispersed Mo-Co dimers anchored on the 2D N-doped graphene to maximize active sites and facilitate nitrogen adsorption and activation, while controlling structural defects such as vacancies in the support could further improve performance. *Predictions: Yield: high; FE: low.*

14.6 A hetero-diatomic catalyst comprising vanadium and chromium atoms embedded in a two-dimensional graphyne matrix could be employed, operating at neutral pH (~7) and an electrode potential near -0.2 V vs RHE in an aqueous 0.1 M Na_2SO_4 electrolyte to maintain catalyst stability and activity. *Predictions: Yield: low; FE: low.*

14.7 An original ammonia electrocatalyst system for nitrogen reduction reaction (NRR) could be designed using a bimetallic single-atom catalyst composed of cobalt (Co) and molybdenum (Mo) atoms dispersed on a nitrogen-doped porous carbon nanofiber (PCNF) matrix. This morphology features high surface area porous nanofibers to maximize active site exposure and facilitate nitrogen adsorption and activation, leveraging the synergistic effect of Co and Mo to enhance NRR activity and selectivity while suppressing competing hydrogen evolution reaction (HER). The catalyst should operate at a mild applied potential around -0.3 V vs. reversible hydrogen electrode (RHE), balancing sufficient driving force for nitrogen reduction with minimized side reactions. The

electrolyte environment may be mildly acidic (pH 5) to optimize proton availability and suppress HER, using a buffered aqueous solution such as 0.1 M phosphate buffer. *Predictions: Yield: high; FE: low.*

14.8 An original ammonia electrocatalyst system may be designed as a heterostructured composite featuring single-atom molybdenum (Mo) doped on a nitrogen-doped porous carbon matrix integrated with ultrathin vanadium nitride (VN) nanosheets. This configuration leverages Mo single atoms for strong nitrogen adsorption and activation, while VN nanosheets provide abundant active sites and excellent electrical conductivity; the nitrogen-doped porous carbon enhances surface area and electron transfer, forming a 3D interconnected porous network that maximizes active site exposure and mass transport. The system shall operate under ambient conditions (room temperature and atmospheric pressure) in a mildly acidic phosphate-buffered electrolyte with pH around 5 to balance proton availability and suppress competing hydrogen evolution reaction (HER). The applied electrode potential should be near -0.25 V, to maximize nitrogen reduction efficiency while minimizing HER. *Predictions: Yield: high; FE: low.*

14.9 An original ammonia electrocatalyst system for nitrogen reduction reaction (NRR) may be designed as a heterostructured composite combining single-atom dispersed vanadium (V) and boron-doped graphitic carbon nitride (g-C_3N_4) nanosheets, where vanadium atoms act as active sites for nitrogen adsorption and activation, and boron doping enhances electronic conductivity and introduces additional active sites. The morphology shall consist of ultrathin, porous nanosheets to maximize surface area and facilitate mass transport of reactants and products. The catalyst may operate optimally under near-neutral pH conditions (around pH 7) using a 0.1 M phosphate buffer electrolyte to balance catalyst stability and nitrogen reduction efficiency. The applied electrode potential shall be approximately -0.2 V versus the reversible hydrogen electrode (RHE), which promotes nitrogen reduction while minimizing competing hydrogen evolution reactions (HER). *Predictions: Yield: high; FE: low.*

14.10 An original ammonia electrocatalyst system may be designed as a heterostructured composite featuring single-atom molybdenum (Mo) doped on a three-dimensional nitrogen-doped porous carbon matrix integrated with vertically aligned vanadium oxide (V_2O_5) nanorods. The Mo single atoms serve as highly active sites for nitrogen adsorption and activation, while the V_2O_5 nanorods enhance electron transfer and structural stability, and the nitrogen-doped porous carbon provides abundant defect sites and high surface area to facilitate nitrogen diffusion and adsorption. This hierarchical morphology maximizes active site exposure and efficient mass transport. The catalyst should operate at an optimized applied potential around -0.35 V versus the reversible hydrogen electrode (RHE), balancing ammonia yield and Faradaic efficiency by minimizing competing hydrogen evolution reaction (HER) and maximizing nitrogen reduction kinetics. The electrolyte pH shall be mildly acidic to neutral (approximately pH 6), using a 0.1 M phosphate buffer solution to maintain stable pH and ionic conductivity, which supports catalyst stability and favorable proton availability without excessive HER. *Predictions: Yield: high; FE: low.*

14.11 An original ammonia electrocatalyst system may be designed as a bimetallic single-atom catalyst composed of molybdenum (Mo) and manganese (Mn) atoms co-anchored on a nitrogen-doped graphene substrate, leveraging Mo's strong nitrogen activation and Mn's tunable electronic properties to synergistically enhance nitrogen reduction reaction (NRR) activity. The morphology shall be a two-dimensional layered structure with atomically dispersed Mo and Mn sites uniformly distributed to maximize active site exposure, supported by the high surface area and conductivity of nitrogen-doped graphene. The catalyst may operate under mildly acidic conditions (pH ~5) using a 0.1 M phosphate buffer solution to balance proton availability and suppress competing hydrogen evolution reaction (HER). The applied electrode potential shall be carefully controlled around -0.35 V versus the reversible hydrogen electrode (RHE) to provide sufficient driving force for nitrogen activation while minimizing HER. Additional structural features such as defect engineering with heteroatoms like boron or sulfur near the active sites may further modulate the electronic environment and enhance catalytic activity and selectivity. *Predictions: Yield: high; FE: low.*

14.12 An original ammonia electrocatalyst system for nitrogen reduction reaction (NRR) may be designed using a composite of single-atom molybdenum (Mo) and iron (Fe) atoms anchored on a nitrogen-doped porous carbon matrix derived from biomass, combining the strong nitrogen activation ability of Mo with the synergistic catalytic effects of Fe. The morphology shall be a 3D hierarchical porous structure or porous nanofiber network to maximize active site exposure, enhance mass transport, and facilitate efficient nitrogen adsorption and activation. Incorporation of heteroatom doping such as boron or sulfur and engineered vacancies (e.g., nitrogen or oxygen vacancies) in the carbon support may further modulate the electronic environment to improve catalytic activity. The electrocatalyst shall operate under neutral pH conditions (around pH 6), using a phosphate buffer electrolyte (e.g., 0.1 M) to maintain stable pH and ionic conductivity, balancing proton availability and suppressing competing hydrogen evolution reaction (HER). The applied electrode potential is proposed to be -0.3 V versus the reversible hydrogen electrode (RHE), optimizing nitrogen reduction kinetics while minimizing side reactions and energy consumption. *Predictions: Yield: high; FE: low.*

14.13 An original ammonia electrocatalyst system may be designed as a heterostructured composite combining vanadium-doped molybdenum phosphide (V-MoP) nanoparticles anchored on nitrogen-doped porous carbon nanosheets, forming a flower-like nanostructure to maximize active site exposure and mass transport. The vanadium doping introduces abundant oxygen vacancies and active sites for nitrogen adsorption, while molybdenum phosphide provides high intrinsic catalytic activity; the nitrogen-doped carbon ensures high surface area and excellent conductivity for efficient electron transfer. Alternatively, a boron-doped high-entropy alloy (HEA) nanostructure integrated with nitrogen-rich porous carbon nanotubes (CNTs) could be employed, where boron doping creates active vacancies and acts as an electron acceptor, and the HEA offers multiple metal active sites, with the porous CNT network suppressing competing hydrogen evolution reaction (HER) and facilitating electron transfer. The catalyst morphology should be engineered as ultrathin nanosheets or interconnected porous nanosponges to

enhance surface area and mass transport. The system shall operate at a neutral pH (around 6) using a 0.1 M phosphate buffer solution to balance proton availability and suppress HER, with an applied electrode potential optimized at -0.3 V versus the reversible hydrogen electrode (RHE) to drive nitrogen reduction efficiently while minimizing side reactions. *Predictions: Yield: high; FE: low.*

15. Validation and Feasibility of the Predicted Mo-W/Ti_2NO_2 Catalyst

15.1 Computational Methodology

All spin-polarized Density Functional Theory (DFT) calculations were performed with the Vienna Ab initio Simulation Package (VASP)[11]. Electron exchange-correlation effects were described using the Perdew-Burke-Ernzerhof (PBE) functional within the generalized gradient approximation (GGA)[12]. A plane-wave cutoff energy of 450 eV was employed. The Brillouin zone was sampled using a Gamma-centered Monkhorst–Pack k-point mesh of 12×12×1 for structural optimizations and 1×1×1 for the *ab initio* molecular dynamics (AIMD) simulations[13]. A vacuum layer of approximately 15 Å was included in the z-direction to prevent spurious interactions between periodic images. The convergence criteria for the electronic self-consistent field (SCF) loop and ionic relaxations were set to 10^{-5} eV and 0.05 eV/Å, respectively. Van der Waals (vdW) interactions were accounted for using Grimme's DFT-D3 correction with zero damping[14]. The AIMD simulation was conducted within the canonical (NVT) ensemble for 10 ps with a time step of 2 fs. The temperature was maintained at 500 K with a Nosé-Hoover thermostat[15-17]. Structural visualizations were generated with the Atomic Simulation Environment (ASE)[18].

15.2 AIMD Stability Analysis and Experimental Feasibility

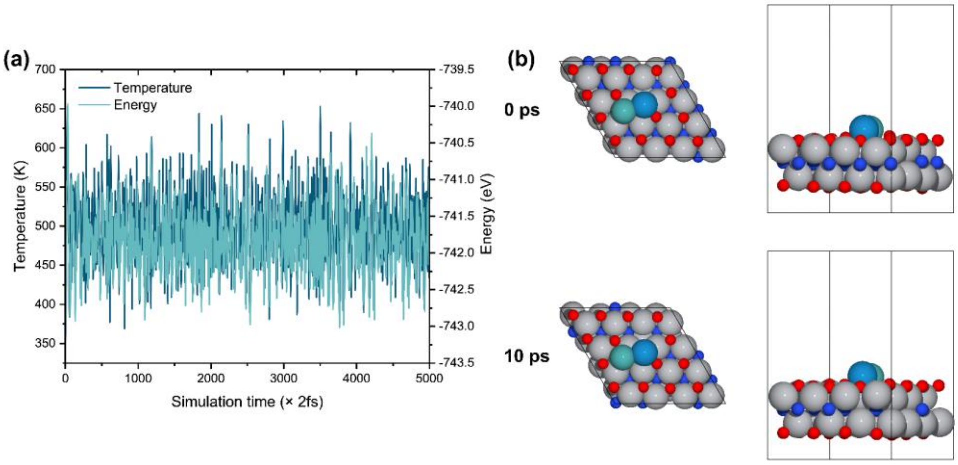

Figure S33. Thermodynamic Stability of the Mo-W Dimer on Ti_2NO_2 MXene from AIMD Simulation. (a) Evolution of system temperature and potential energy over a 10 ps simulation at 500 K, demonstrating thermodynamic stability. (b) Top and side views of the atomic structure at the beginning (0 ps) and end (10 ps) of the simulation. The Mo-W dimer remains intact without dissociation or agglomeration. Atom colors: Ti (gray), O (red), N (small blue), Mo (light cyan), W (dark blue).

To validate our model's top prediction, we performed AIMD simulations to assess the thermodynamic stability of the Mo-W dimer on the Ti_2NO_2 MXene surface. As shown in Figure S33a, the system's potential energy and temperature remain stable throughout the 10 ps simulation at 500 K, indicating that the structure is not undergoing any major reconstruction and is dynamically stable. The structural snapshots in Figure S33b confirm this stability, showing that the Mo-W dimer maintains its integrity without dissociating or agglomerating. This result is particularly compelling when contrasted with literature reports showing that Mo-Mo homodimers on Ti_2NO_2 support are unstable, tending to disperse into isolated atoms within 3 ps[19]. The ability of our model to correctly identify the stable heterodimer, powerfully validates its capacity to discern subtle, composition-dependent effects critical for catalyst design.

Having computationally established its stability, we considered its experimental feasibility. Though direct synthesis has not yet been reported, a plausible route exists based on established methods. This could involve a multi-step process: (1) selective etching of the MAX phase precursor via a molten salt route to produce Cl-terminated MXenes[20], (2) *in-situ* functionalization to introduce the desired -O surface groups, and (3) precise loading of the Mo-W dimer with a technique like Atomic Layer Deposition (ALD)[21], which offers atomic-level control.

16. OERCrew

To demonstrate the scalability of our designed multi-agent framework, we have developed a system called OERCrew, designed for studying the structure-activity relationships in the oxygen evolution reaction (OER). As is well known, the OER is a crucial half-reaction in water splitting and metal-air batteries, playing a significant role in the conversion and storage of renewable energy. However, the sluggish kinetics of the OER pose significant challenges like high overpotentials and substantial energy losses, which necessitates innovative research methods to advance the rational design of OER electrocatalysts. By leveraging the advanced multi-agent framework, the OERCrew is capable of predicting the reaction overpotential for the OER under test conditions of a current density of 10 $mA\,cm^{-2}$. It provides evidence-based OER-related responses and allows interaction with our curated OER data through natural language, offering both qualitative and quantitative insights. By facilitating seamless access to structured data and predictive models, OERCrew empowers researchers to uncover trends and correlations that cannot be immediately apparent through traditional analysis methods.

We constructed a literature corpus related to OER, using the query, "Oxygen Evolution Reaction" OR OER OR "Electrocatalytic Oxygen Evolution" OR "Oxygen Production" OR "Water Oxidation" (Title) AND "overpotential" (Topic). And we restricted the publication years from 2020 to 2024, focused on articles, and included only highly cited papers, resulting in the retrieval of 172 articles. Utilizing the text-mining workflow shown in Figure 1 in the manuscript, we extracted 186 pieces of information about the structure-activity relationships of the OER. This dataset included details such as reaction types, products, reactants, elemental composition and structures of OER electrocatalysts, pH types, overpotentials, and corresponding current densities. We then eliminated data unrelated to OER or lacking overpotential information. We found that entries with a current density of 10 $mA\,cm^{-2}$ accounted for 63.1% of the dataset. To facilitate the subsequent machine learning model training, we selected data corresponding to this test condition,

resulting in 111 entries related to OER structure-activity relationships. Following the training procedures of the eNRR yield and Faradaic efficiency predictive models as detailed in the manuscript, we conducted OER overpotential predictions under the condition of a current density of 10 mA cm^{-2}. The distribution of the OER overpotential with the database is shown in Figure S34a, the majority of the data points cluster around the 200-300 mV range. The correlation matrix of features after recursive feature elimination is shown in Figure S34b.

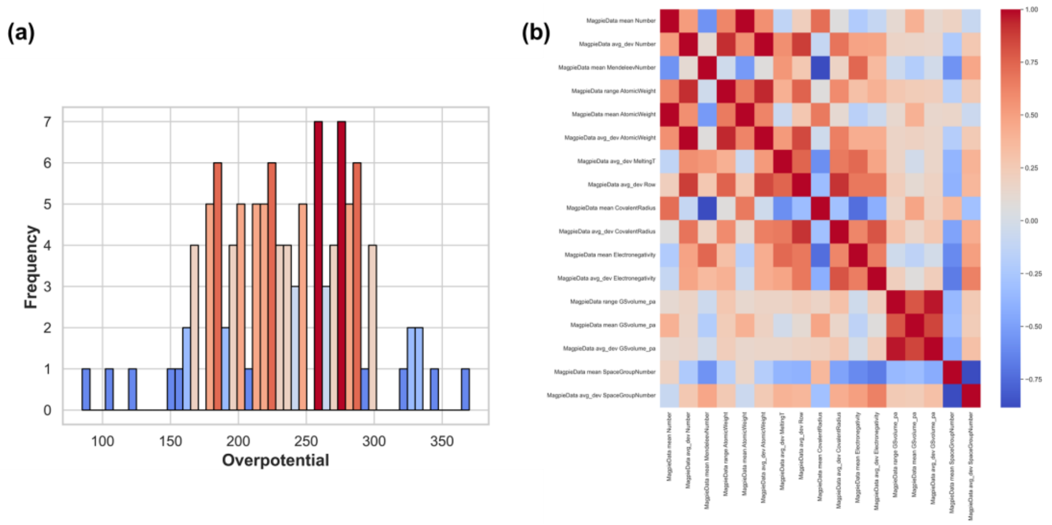

Figure S34. (a) Histograms illustrating the distribution of OER overpotential at a current density of 10 mA cm^{-2}. (b) The correlation coefficient matrix of the features retained after recursive feature elimination for predicting OER overpotential at a current density of 10 mA cm^{-2}.

We developed a random forest classification model to predict the overpotential, categorizing results equal to or above the median as high overpotential. Figure S35c displays the mean test scores across iterations during hyperparameter tuning with Optuna. After approximately 400 iterations, the scores stabilized, indicating that Optuna successfully identified optimal hyperparameters. The confusion matrices shown in Figure S35a illustrate the model's performance on both the training and test sets. The classifier achieved an accuracy of 0.82 and 0.55 for training and test sets, respectively. The ROC curves in Figure S35b provide further insight into the model's performance. The training set achieved an AUC of 0.88 and the test set AUC was 0.52.

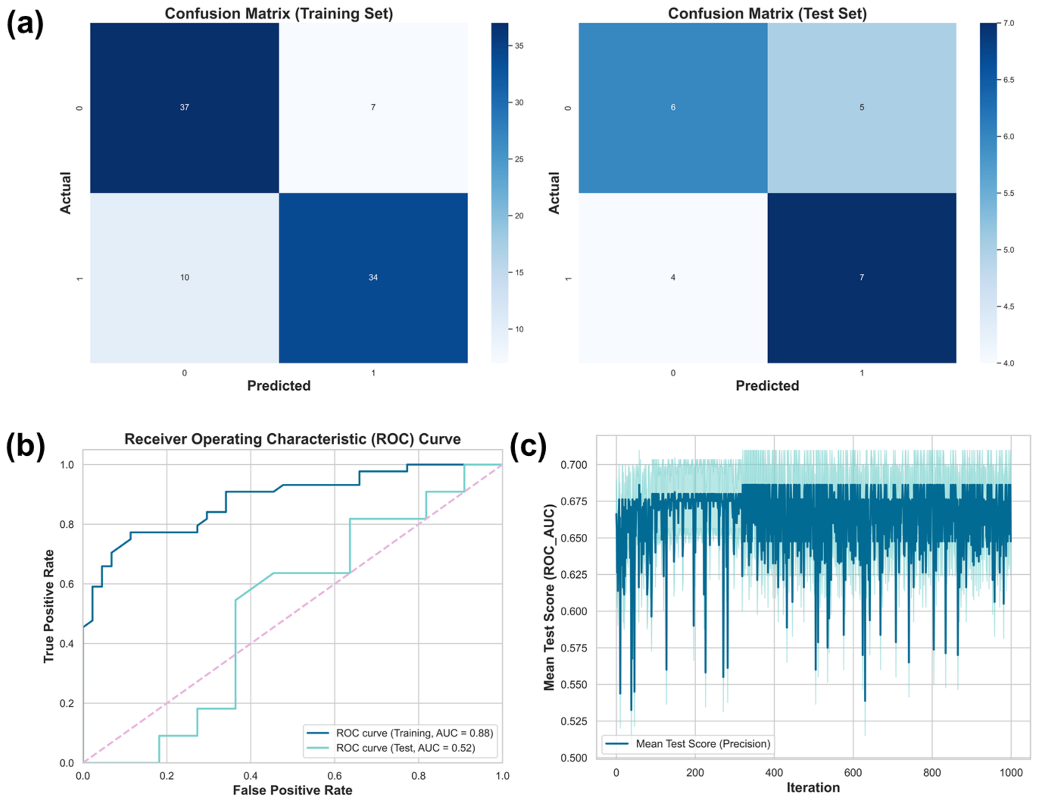

Figure S35. (a) Confusion matrices for the random forest classifier on the training set (left) and test set (right) for OER overpotential. (b) ROC curve, showing the true positive rate against the false positive rate, with AUC values indicating model effectiveness. (c) Mean test scores (ROC AUC) over iterations during hyperparameter tuning using Optuna.

Based on the OER corpus we obtained from the text-mining workflow, the curated database, and the random forest classifier for predicting the overpotential at a current density of 10 mA cm^{-2}, we also developed the OERCrew as depicted in Figure S36. The OERCrew consists of four agents, an orchestrator; a GraphRAG retriever, which enhances responses by retrieving information from our curated databases containing OER abstracts as illustrated in Figure S37; a CSV file handler shown in Figure S38a, which writes and executes code to interact with CSV files obtained from text-mining workflow in response to user queries; and a overpotential predictor, which predicts the likelihood of high overpotential (corresponding to 1) as shown in Figure S38b and Figure S39. The clear distinction between the two categories suggests that the predictive model effectively differentiates between high and low overpotential scenarios.

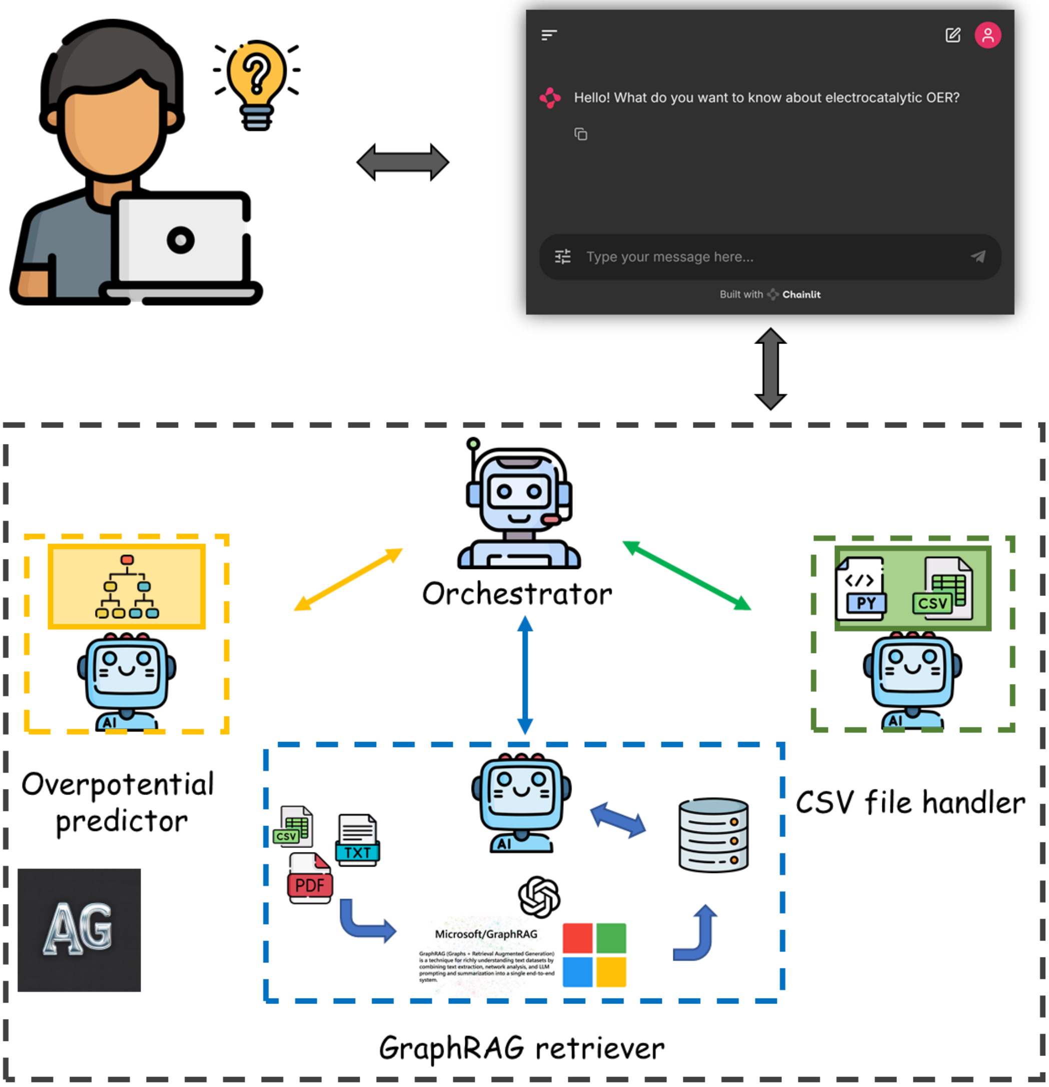

Figure S36. The schematic image of OERCrew. The OERCrew comprises four agents, an orchestrator, an overpotential predictor, a GraphRAG retriever, and a CSV file handler. Users interact with OERCrew through the user interface provided by the Chainlit library to perform retrieval and prediction of structure-activity relationships in OER. This figure has been designed using resources from Flaticon.com.

```
Question: How to reduce the overpotential of the OER through catalyst design?
--------------------------------------------------------------------------------
Next speaker: Retriever

Retriever (to chat_manager):

***** Suggested function call: query_graphRAG *****
Arguments:
{"question":"How to reduce the overpotential of the oxygen evolution reaction (OER) through catalyst design?"}
****************************************************
--------------------------------------------------------------------------------
Next speaker: User_Proxy

>>>>>>>> NO HUMAN INPUT RECEIVED.

>>>>>>>> USING AUTO REPLY...

>>>>>>>> EXECUTING ASYNC FUNCTION query_graphRAG...
<Details of query_graphRAG execution...>

User_Proxy (to chat_manager):

***** Response from calling function (query_graphRAG) *****
  To reduce the overpotential of the oxygen evolution reaction (OER) through catalyst design, several strategies may be employed. Firstly, the careful selection of electrocatalyst compositions, such as Co-Cu-Fe-Mo (oxy)hydroxide, has demonstrated a low overpotential of 199 mV at a current density of 10 mA cm(-2), indicating that specific metal combinations can significantly enhance OER activity [Data: Reports (7, 4, 5)]. Additionally, utilizing hybrid materials and composite structures can lead to improved performance metrics, as recent advancements focus on optimizing these materials for better efficiency in the OER [Data: Reports (6, 9, 1, 18, 2, +more)]. Incorporating transition metals like Fe, Co, and Ni into high-entropy oxides (HEOs) and bimetallic systems has also shown promise, with catalysts such as Fe2Ni MIL-88 achieving low overpotentials [Data: Reports (16, 8)]. Furthermore, designing catalysts with unique structural characteristics, such as the NCO-NNs@NFO-NFs/NF heterostructure, which maintains stable performance over extended periods, highlights the importance of structural design in reducing overpotential [Data: Reports (16, 8)]. Doping existing electrocatalysts with elements like nitrogen or rhodium can significantly improve their reaction pathways and overall efficiency, thereby reducing the required overpotential for the OER [Data: Reports (9, 1, 2, 0, 3, +more)]. Lastly, the choice of electrolyte, whether alkaline or acidic, plays a crucial role in influencing the electrochemical environment, which may lead to reduced overpotentials during the OER [Data: Reports (1, 2, 0, 3, 18, +more)].
************************************************************
--------------------------------------------------------------------------------
Next speaker: User_Proxy
```

Figure S37. Dialogue showing the OERCrew providing the answer to how to reduce the overpotential of OER.

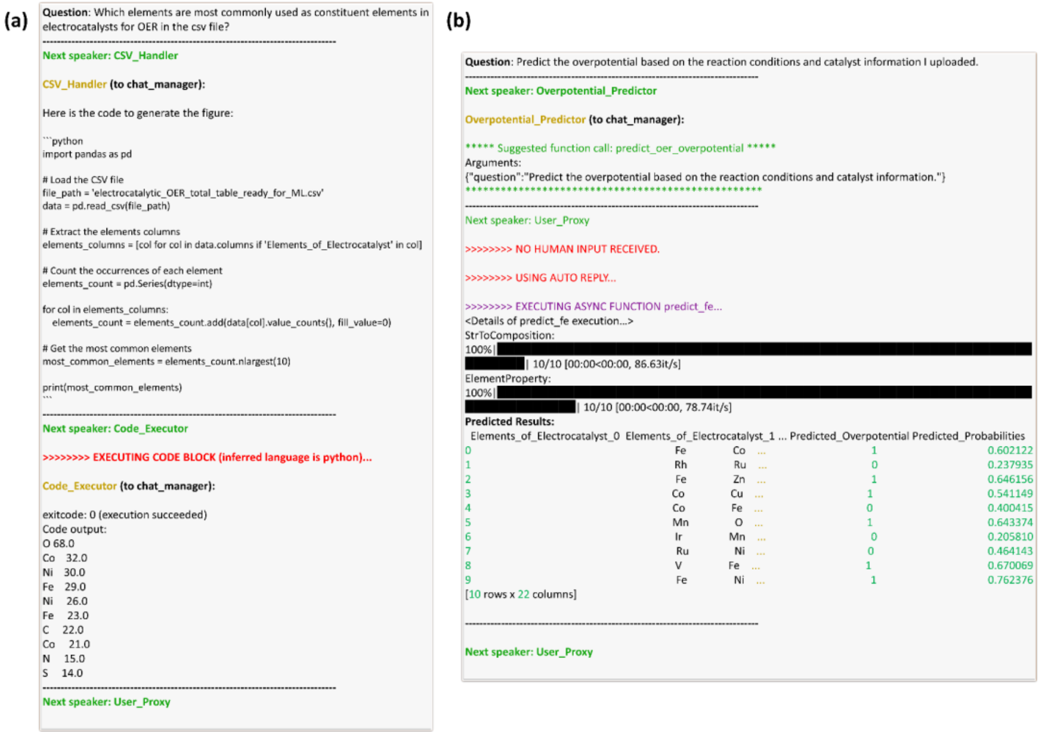

Figure S38. (a) Dialogue showing the OERCrew providing Python code to answer which elements are most commonly used as constituent elements in electrocatalysts for OER. (b) Dialogue illustrating the use of the OERCrew to predict overpotential.

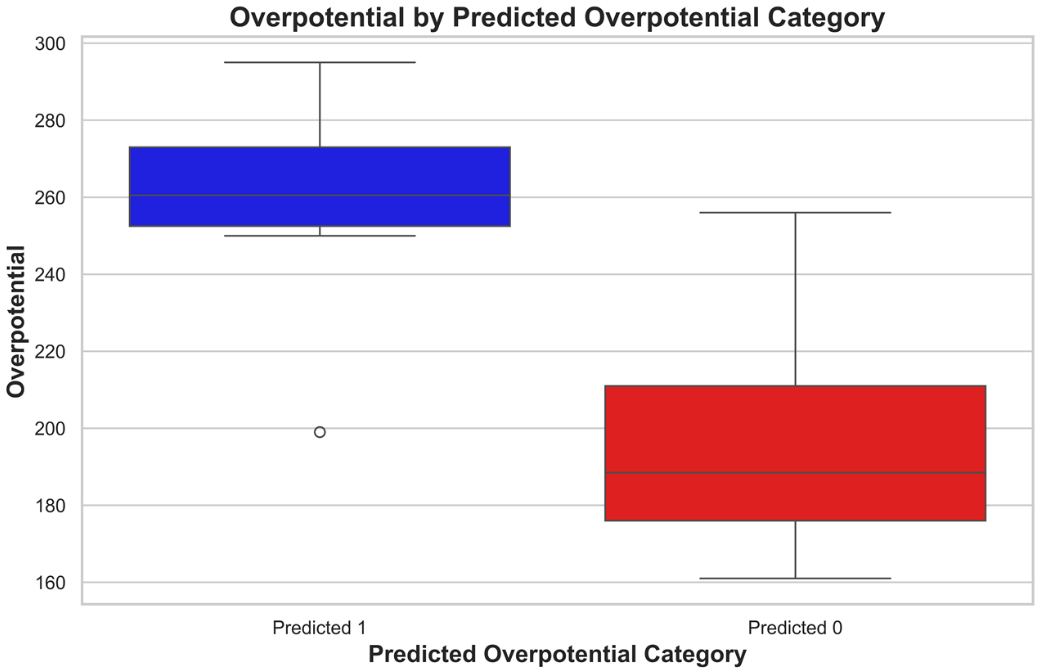

Figure S39. The predictions versus experimental OER overpotential at 10 mA cm^{-2} correspond to Figure S38b.

References

1. Liu R, Fei H, Wang J *et al.* Unveiling the synergistic effect between the metallic phase and bridging S species over MoS_2 for highly efficient nitrogen fixation. *Appl Catal B-environ* 2024; **343**: 123469-123477.
2. Liu B, Ma C, Liu D *et al.* Sulfur-vacancy defective MoS_2 as a promising electrocatalyst for nitrogen reduction reaction under mild conditions. *ChemElectroChem* 2021; **8**: 3030-3039.
3. Liu Z, Moroz Y S, and Isayev O. The challenge of balancing model sensitivity and robustness in predicting yields: a benchmarking study of amide coupling reactions. *Chem Sci* 2023; **14**: 10835-10846.
4. Voinarovska V, Kabeshov M, Dudenko D *et al.* When yield prediction does not yield prediction: an overview of the current challenges. *J Chem Inf Model* 2024; **64**: 42-56.
5. Dalmau D and Alegre-Requena J V. ROBERT: Bridging the gap between machine learning and chemistry. *WIREs Computational Molecular Science* 2024; **14**: e1733.
6. Xin H, Vojvodic A, Voss J *et al.* Effects of *d*-band shape on the surface reactivity of transition-metal alloys. *Phys Rev B* 2014; **89**: 115114.
7. Zhang Y, Ma N, Wang Y *et al.* Theoretical design toward highly efficient single-atom catalysts for nitrogen reduction by regulating the “acceptance-donation” mechanism. *Appl Surf Sci* 2023; **623**: 156827.
8. Yuan M, Bai Y, Zhang J *et al.* Work function regulation of nitrogen-doped carbon nanotubes triggered by metal nanoparticles for efficient electrocatalytic nitrogen fixation. *J Mater Chem A* 2020; **8**: 26066-26074.
9. Yang C, Huang B, Bai S *et al.* A generalized surface chalcogenation strategy for boosting the electrochemical N_2 fixation of metal nanocrystals. *Adv Mater* 2020; **32**: e2001267.
10. Wu T, Melander M M, and Honkala K. Coadsorption of NRR and HER intermediates determines the performance of Ru-N_4 toward electrocatalytic N_2 reduction. *ACS Catal* 2022; **12**: 2505-2512.
11. Kresse G and Furthmüller J. Efficiency of ab-initio total energy calculations for metals and semiconductors using a plane-wave basis set. *Comp Mater Sci* 1996; **6**: 15-50.
12. Perdew J P, Burke K, and Ernzerhof M. Generalized gradient approximation made simple. *Phys Rev Lett* 1996; **77**: 3865-3868.
13. Monkhorst H J and Pack J D. Special points for Brillouin-zone integrations. *Phys Rev B* 1976; **13**: 5188-5192.
14. Grimme S, Antony J, Ehrlich S *et al.* A consistent and accurate ab initio parametrization of density functional dispersion correction (DFT-D) for the 94 elements H-Pu. *J Chem Phys* 2010; **132**: 154104.
15. Nosé S. A unified formulation of the constant temperature molecular dynamics methods. *J Chem Phys* 1984; **81**: 511-519.
16. Shuichi N. Constant temperature molecular dynamics methods. *Prog Theor Phys Supp* 1991; **103**: 1-46.
17. Hoover W G. Canonical dynamics: Equilibrium phase-space distributions. *Phys Rev A* 1985; **31**: 1695-1697.
18. Hjorth Larsen A, Jørgen Mortensen J, Blomqvist J *et al.* The atomic simulation environment—a Python library for working with atoms. *J Phys Condens Mat* 2017; **29**: 273002.
19. Cheng Y, Dai J, Song Y *et al.* Single molybdenum atom anchored on 2D Ti_2NO_2 MXene as a

promising electrocatalyst for N_2 fixation. *Nanoscale* 2019; **11**: 18132-18141.

20. Anasori B, Lukatskaya M R, and Gogotsi Y. 2D metal carbides and nitrides (MXenes) for energy storage. *Nat Rev Mater* 2017; **2**: 16098.

21. Yan H, Lin Y, Wu H *et al*. Bottom-up precise synthesis of stable platinum dimers on graphene. *Nat Commun* 2017; **8**: 1070.
